# Prise en charge de la multimorbidité cœur–cerveau : un guide de pratique clinique

**DOI:** 10.1503/cmaj.251137-f

**Published:** 2026-05-25

**Authors:** Jodi D. Edwards, Zhe Li, Philip McFarlane, Doreen M. Rabi, Jeremy Gilbert, Harpreet S. Bajaj, Bradley J. MacIntosh, Jesse Bittman, Ross D. Feldman, George Dresser, Kristin Terenzi, Rick Swartz, Jonathan Gabor, Glen J. Pearson, Peter Selby, Sean Wharton, Darren E.R. Warburton, Smita Pakhalé, Rima Styra, Brian Baker, Karen Tu, Michael Hawkins, James A. Stone, Tracy Vaillancourt, Stephanie Poon, Sean A. Virani, Rahul Jain, Peter Hayward Jones, Roopinder K. Sandhu, Aravind Ganesh, Jason G. Andrade, Sol Stern, Jeffrey Habert, Léna Rivard, Paul Roumeliotis, Jacob A. Udell, Tavis Campbell, Simon L. Bacon, Luc Trudeau, Karim Keshavjee, Thuy Pham, Gemma Cheng, Krystina B. Lewis, Marion Maar, Dawn Stacey, Brian Oldenburg, Abida R. Dhukai, Sachin V. Pasricha, Diana Sherifali, Megan Racey, Diane Hua-Stewart, Christopher A. Gravel, Meghan Lewis, Sumali Mehta, Michael D. Hill, Nazia Haider, Patrice Lindsay, Peter Liu, Sheldon W. Tobe

**Affiliations:** Institut de cardiologie de l’Université d’Ottawa (Edwards, Li, Vaillancourt, K.B. Lewis, Stacey, Liu); École d’épidémiologie et de santé publique (Edwards, Pakhalé, Roumeliotis, Gravel, M. Lewis, Mehta, Liu), Université d’Ottawa, Ottawa, Ont.; Département de médecine (McFarlane, Gilbert, Selby, Tu, Poon, Udell, Dhukai, Pasricha, Tobe), Temerty Faculty of Medicine, University of Toronto, Toronto, Ont.; Sunnybrook Health Sciences Centre (Gilbert, MacIntosh, Swartz, Poon, Jain, Pham, Dhukai, Hua-Stewart, Tobe), Toronto, Ont.; LMC Diabetes & Endocrinology (Bajaj), Brampton, Ont.; Département de médecine (Bittman, Virani), University of British Columbia, Vancouver, C.-B.; Western University (Feldman); Département de médecine (Dresser), Western University, London, Ont.; Humber River Hospital (Terenzi), Toronto, Ont.; Département de médecine (Gabor), Concordia General Hospital, Winnipeg, Man.; Département de cardiologie (Gabor), Selkirk Regional Health Centre, Selkirk, Man.; Département de médecine (Pearson), division de cardiologie, University of Alberta, Calgary, Alb.; Faculté des sciences de la santé (Wharton), McMaster University, Hamilton, Ont.; Faculté d’éducation (Warburton), Programme de santé et d’activité physique autochtone, University of British Columbia, Vancouver, C.-B.; Département de psychiatrie (Styra, Baker, Hawkins), University of Toronto; division cardiovasculaire (Stone, Udell), Women’s College Hospital; Département de médecine familiale et communautaire (Jain, Habert), Temerty Faculty of Medicine, University of Toronto; OCAD University (Jones), Toronto, Ont.; School of Architecture, Art and Design (Jones), Tecnológico de Monterrey, Mexico City, Mexique; Calgary Stroke Program, Départements de neurosciences cliniques (Ganesh, Rabi, Campbell, Hill), de radiologie et de sciences de la santé communautaire (Ganesh), Hotchkiss Brain Institute (Ganesh, Rabi, Campbell, Hill), Département des sciences cardiaques, Libin Cardiovascular Institute (Sandhu), O’Brien Institute for Public Health (Ganesh), Cumming School of Medicine, University of Calgary, Calgary, Alb.; Vancouver General Hospital (Andrade), Vancouver, C.-B.; médecin de famille (Stern), Oakville, Ont.; Département de médecine et Centre de recherche (Rivard), Institut de cardiologie de Montréal, Université de Montréal, Montréal, Qc; Peter Munk Cardiac Centre (Udell), University Health Network, Toronto, Ont.; Département de médecine (Gabor), Département de santé, de kinésiologie et de physiologie appliquée (Bacon), Université Concordia, Montréal, Qc; Hôpital général juif (Trudeau), Université McGill, Montréal, Qc; Institute of Health Policy, Management and Evaluation (Keshavjee), Dalla Lana School of Public Health, University of Toronto, Toronto, Ont.; Département de médecine (Cheng), Université McGill, Montréal, Qc; École des sciences infirmières (K.B. Lewis), Faculté des sciences de la santé, Université d’Ottawa, Ottawa, Ont.; Université de l’École de médecine du Nord de l’Ontario (Maar, Tobe), Sudbury, Ont.; Centre de recherche sur la mise en œuvre (Stacey), Institut de recherche de l’Hôpital d’Ottawa, Ottawa, Ont.; Département de santé publique et des sciences de la mise en œuvre (Oldenburg), La Trobe University et Baker Heart and Diabetes Institute, Melbourne, Australie; École de sciences infirmières (Sherifali, Racey), McMaster University, Hamilton, Ont.; Département de mathématiques et de statistiques (Gravel), et Institut de recherche en littératie des données (Gravel), Université d’Ottawa, Ottawa, Ont.; Informatique de la santé (Haider), University of Toronto, Toronto, Ont.; MarcLind Health Systems and Engagement Consulting (Lindsay), Toronto, Ont.

## Abstract

**Contexte:**

Les maladies du cœur et les maladies du cerveau partagent divers facteurs de risque et elles coexistent souvent; pourtant, les guides de pratique clinique en cardiologie et en neurologie sont le plus souvent produits en silos disciplinaires. Élaboré par un groupe de personnes expertes du projet C-CHANGE (Canadian Cardiovascular Harmonized National Guideline Endeavour), le présent guide de pratique clinique vise à élargir les recommandations actuelles sur les affections cardiovasculaires pour y intégrer des données probantes provenant de la documentation en neurologie et en santé mentale, ainsi que des recommandations spécifiquement destinées aux prestataires de soins qui prennent en charge des affections concomitantes du cœur et du cerveau.

**Méthodes:**

Le comité de rédaction qui a élaboré le présent guide de pratique clinique comprenait un comité directeur; 10 sous-comités de personnes expertes chargées de formuler des questions de recherche et de rédiger une première version des recommandations portant sur des affections cœur–cerveau particulières; une équipe d’analyse des données probantes pour garantir la rigueur de la méthodologie et son application cohérente; et un comité de mise en œuvre pour faciliter l’adoption des recommandations du guide par les cliniciennes et cliniciens ainsi que l’intégration de ces recommandations aux dossiers médicaux électroniques. La McMaster Evidence Review and Synthesis Team a apporté son soutien aux recherches documentaires et à l’évaluation critique de la documentation. Un groupe de personnes qui ont une expérience vécue (PEV) des affections concernées, y compris des personnes proches aidantes, a apporté son éclairage sur les valeurs et les points de vue de la patientèle durant tout le processus d’élaboration du présent guide. Notre processus d’atteinte du consensus a respecté les principes de l’outil Appraisal of Guidelines for Research and Evaluation II. Nous avons utilisé une méthode reconnue d’évaluation des données probantes pour déterminer le degré de certitude des données probantes et la force de chaque recommandation. Enfin, nous avons adopté les principes du Guidelines International Network pour la gestion des intérêts concurrents.

**Recommandations:**

Nous avons formulé 11 recommandations sur la prise en charge de diverses maladies cœur–cerveau concomitantes. Les principales recommandations comprennent le dépistage du déclin cognitif en cas de fibrillation auriculaire et de la dépression en cas de coronaropathie; le traitement de la dépression en cas de coronaropathie, de troubles cognitifs en cas d’hypertension et de la dyslipidémie en cas d’accident vasculaire cérébral (AVC); ainsi que la vaccination pour prévenir l’AVC, l’infarctus du myocarde et la démence. Nous recommandons également la prise de décision partagée, ce qui comprend le recours à des outils d’aide à la décision fondés sur des données probantes, afin de soutenir la patientèle aux prises avec des maladies cœur–cerveau.

**Interprétation:**

Nous avons cherché à produire un guide de pratique clinique applicable et pratique pour le dépistage et la prise en charge de la patientèle aux prises avec des maladies cœur–cerveau. Notre guide s’adresse principalement aux prestataires de soins primaires, mais il est également pertinent pour faciliter la prestation et l’individualisation des soins de surspécialités et pour aider les équipes interprofessionnelles qui prennent en charge des personnes atteintes de maladies cœur–cerveau concomitantes.

Les maladies du cœur et les maladies du cerveau touchent des millions de personnes et sont les principales causes de mortalité et de morbidité à l’échelle mondiale. La cardiopathie ischémique cause quelque 16 % des décès dans le monde^1^,^2^; les accidents vasculaires cérébraux (AVC) et la démence figurent parmi les principales causes de pertes d’années de vie en bonne santé et d’incapacité, et coûtent des milliards de dollars chaque année aux systèmes de santé^3^,^4^. Bien que les maladies du cœur et les maladies du cerveau aient des facteurs de risque en commun et qu’elles coexistent souvent^5^,^6^, les guides de pratique clinique en cardiologie, en neurologie et en santé mentale sont généralement produits en silos disciplinaires^7^–^9^.

Lancée en 2008, l’initiative C-CHANGE (Canadian Cardiovascular Harmonized National Guideline Endeavour)^10^ visait à fournir aux prestataires de soins du Canada un ensemble harmonisé de recommandations sur les affections cardiovasculaires provenant des principaux organismes canadiens responsables de guides de pratique clinique en médecine cardiovasculaire, de manière à aider les équipes de soins à élaborer des plans de traitement complets pour la patientèle touchée par la multimorbidité^11^.

L’élargissement du processus C-CHANGE résulte de notre compréhension croissante de l’importance des liens entre les maladies du cœur et les maladies du cerveau, et du rôle crucial de ces liens dans la multimorbidité chez les populations vieillissantes. Il existe en effet une interaction étroite entre les maladies du cœur et les maladies du cerveau, plusieurs maladies concomitantes ayant en commun des facteurs de risque^12^, des mécanismes physiopathologiques ainsi que de potentiels liens génétiques et phénotypiques^13^,^14^. La coexistence d’affections du cœur et du cerveau est donc fréquente, et elle accroît les risques réciproques. Le présent guide de pratique clinique élargit la portée des recommandations cardiovasculaires actuelles en intégrant les données probantes issues de la documentation en neurologie et en santé mentale.

Le processus C-CHANGE utilise le cadre SMART (spécifique, mesurable, applicable, réaliste et situé dans le temps)^15^ (annexe 1, tableau supplémentaire 1, accessible en anglais au www.cmaj.ca/lookup/doi/10.1503/cmaj.251137/tab-related-content) pour élaborer des recommandations facilement mesurables, prêtes à mettre en œuvre, faciles à intégrer aux dossiers médicaux informatisés et praticables pour les cliniciennes et cliniciens en soins primaires dans les délais spécifiés. Les recommandations du présent guide sont structurées de manière à assurer et à individualiser le processus de diagnostic et de prise en charge de la patientèle touchée par diverses maladies cœur–cerveau. Les guides C-CHANGE adoptent une approche axée sur le patient ou la patiente et tiennent compte des valeurs et préférences exprimées par la personne atteinte^16^.

L’objectif du présent guide de pratique clinique consiste à élargir les recommandations cardiovasculaires actuelles pour y intégrer des données probantes issues de la documentation en neurologie et en santé mentale, et à formuler des recommandations précises à l’intention des prestataires de soins qui prennent en charge des affections cœur–cerveau concomitantes.

## Portée

Le présent guide vise à fournir des recommandations fondées sur des données probantes, à jour et axées sur la personne atteinte pour la prévention et la prise en charge des affections cœur–cerveau, et cela, en intégrant les principes de la prise de décision partagée, des déterminants sociaux de la santé et de l’engagement de la patientèle. Le présent guide s’adresse aux prestataires de soins primaires, mais il sera aussi utile et pertinent pour les surspécialistes en cardiologie, en neurologie et en santé mentale, ainsi que pour les membres des équipes interprofessionnelles qui prennent en charge les maladies cœur–cerveau.

Les tableaux cliniques de la multimorbidité cœur–cerveau sont multiples. Toutefois, dans le présent guide de pratique clinique, le comité de rédaction s’est concentré sur les affections concomitantes pour lesquelles nous estimions que les données probantes étaient les plus robustes, et que notre groupe de PEV (incluant des personnes proches aidantes) jugeait prioritaires. Les thèmes retenus sont donc les liens entre les troubles cognitifs d’une part et, d’autre part, la fibrillation auriculaire et l’hypertension; les liens entre la coronaropathie et la dépression; les liens entre la dyslipidémie d’une part et, d’autre part, l’AVC et le risque global de maladie cardiovasculaire; et, finalement, la vaccination pour prévenir l’AVC, l’infarctus du myocarde et la démence.

La plupart des recommandations seront surtout utiles aux prestataires de soins qui prennent en charge des adultes d’âge moyen et des personnes âgées présentant des facteurs de risque de maladie cardiovasculaire, mais certaines recommandations (p. ex., celles portant sur la dépression) peuvent s’étendre à des populations plus larges. Pour chaque recommandation, nous proposons également des considérations relatives au sexe et au genre, le sexe désignant le sexe biologique, et le genre renvoyant à la construction sociale, psychologique et culturelle de l’identité de genre. Toutefois, si la plupart des études ont recueilli ou présenté les données selon le sexe, peu d’entre elles comportaient des informations sur l’identité de genre.

## Recommandations

Les recommandations ont été élaborées par 10 sous-comités composés de personnes expertes (annexe 2, figure supplémentaire 1, accessible en anglais au www.cmaj.ca/lookup/doi/10.1503/cmaj.251137/tab-related-content), examinées parallèlement par notre groupe de PEV et approuvées par le comité de rédaction du guide. Le processus de consensus (décrit dans la section « Méthodes » et dans l’annexe 2, figure supplémentaire 2) a permis de parvenir à un total de 11 recommandations distinctes pour les maladies cœur–cerveau concomitantes. Les recommandations sont divisées en recommandations sur le dépistage et recommandations sur le traitement (tableau 1). Une dernière recommandation reflète les données probantes actuelles portant sur la prise de décision partagée et sur l’utilisation d’outils d’aide à la décision destinés à la patientèle; cette recommandation s’applique à l’ensemble des recommandations sur la multimorbidité cœur–cerveau.

**Tableau 1:** Recommandations pour le dépistage, le traitement et la prise de décision partagée, relativement aux maladies cœur–cerveau concomitantes

Thème	Recommandation	Degré de certitude des données probantes; force de la recommandation
**Dépistage**		
Fibrillation auriculaire et risque de troubles cognitifs	Nous suggérons de soumettre les personnes atteintes de fibrillation auriculaire à un dépistage au moyen d’un instrument validé afin de repérer celles qui sont exposées à un risque de troubles cognitifs.	Degré de certitude des données probantes 2A; force de la recommandation B
Coronaropathie et dépression	Nous suggérons de soumettre les personnes atteintes de coronaropathie à un dépistage de la dépression au moyen d’un instrument validé.	Degré de certitude des données probantes 1B; force de la recommandation B
**Traitement**		
Coronaropathie et dépression	Chez les personnes atteintes de coronaropathie dont le diagnostic de dépression est confirmé, nous recommandons d’entreprendre un traitement par ISRS; ou, selon la gravité de la maladie, nous suggérons un autre traitement fondé sur des données probantes, comme la thérapie cognitivo-comportementale.	Degré de certitude des données probantes 1A; force de la recommandation A Degré de certitude des données probantes 1B; force de la recommandation C
Hypertension et risque de troubles cognitifs	Nous recommandons que les personnes dont la PA systolique se situe entre 130 mm Hg et 180 mm Hg et qui présentent un risque cardiovasculaire accru (déterminé par la présence d’une maladie cardiovasculaire clinique ou subclinique, d’une maladie rénale chronique, d’un score de Framingham ≥ 15 %, ou d’un âge ≥ 75 ans) reçoivent un traitement intensif visant une PA systolique inférieure à 120 mm Hg afin de réduire le risque de troubles cognitifs.	Degré de certitude des données probantes 1B; force de la recommandation A
*Dyslipidémie et risque d’AVC ou de maladie cardiovasculaire*		
Dyslipidémie et risque d’AVC ou de maladie cardiovasculaire	Nous recommandons que les personnes atteintes de coronaropathie dont le taux de cholestérol LDL dépasse le seuil de 1,8 mmol/L reçoivent un traitement intensifié, de manière à abaisser le taux de cholestérol LDL en deçà de la cible, afin de prévenir l’AVC et de réduire le risque cardiovasculaire.	Degré de certitude des données probantes 1A; force de la recommandation A
	Nous suggérons que les victimes d’AVC dont le taux de cholestérol LDL dépasse le seuil de 1,8 mmol/L reçoivent un traitement intensifié, de manière à abaisser le taux de cholestérol LDL en deçà de la cible, afin de réduire le risque d’événements cardiovasculaires majeurs.	Degré de certitude des données probantes 1B; force de la recommandation B
*Vaccination et risque d’AVC, de décès d’origine cardiovasculaire, d’infarctus du myocarde ou de démence*		
Vaccination contre la grippe	Nous suggérons d’offrir systématiquement à toutes les personnes, et en particulier à celles de plus de 65 ans, la vaccination contre la grippe afin de réduire le risque de décès d’origine cardiovasculaire, d’AVC et, possiblement, de démence.	Décès d’origine cardiovasculaire et AVC : Degré de certitude des données probantes 2A; force de la recommandation C Démence : Degré de certitude des données probantes 3; force de la recommandation C
Vaccination contre les infections à pneumocoque	Nous suggérons d’offrir systématiquement à toutes les personnes, et en particulier à celles de plus de 65 ans, la vaccination contre les infections à pneumocoque afin de réduire le risque d’infarctus du myocarde et d’AVC.	Degré de certitude des données probantes 2A; force de la recommandation B
Vaccination contre le zona	Nous suggérons d’offrir systématiquement à toutes les personnes, et en particulier à celles de plus de 65 ans, la vaccination contre le zona afin de réduire le risque d’infarctus du myocarde et d’AVC, ainsi que, possiblement, de démence.	Infarctus du myocarde et AVC : Degré de certitude des données probantes 2A; force de la recommandation B Démence : Degré de certitude des données probantes 3; force de la recommandation C
**Prise de décision partagée**		
Prise de décision partagée et santé cœur–cerveau	Nous recommandons que les cliniciennes et cliniciens encouragent les personnes atteintes d’affections cœur–cerveau, ou qui risquent d’en être atteintes, à participer activement à la prise de décision concernant leurs soins, et cela en utilisant avant et pendant les consultations des outils d’aide à la décision fondés sur des données probantes, afin de faciliter la prise de décision.	Degré de certitude des données probantes 1A; force de la recommandation B

Remarque : AVC = accident vasculaire cérébrale; ISRS = inhibiteur sélectif de la recapture de la sérotonine; LDL = lipoprotéines de basse densité; PA = pression artérielle.

Comme le préconisent Woolf et ses collègues^17^, nous fournissons pour chaque recommandation les fondements de notre décision, soit un résumé des données probantes et une évaluation de la qualité (du degré de certitude) de ces dernières. Nous fournissons aussi des renseignements complémentaires sur chaque recommandation afin d’aider les prestataires de soins à l’intégrer dans leur pratique (annexe 3, accessible en anglais au www.cmaj.ca/lookup/doi/10.1503/cmaj.251137/tab-related-content). Nous avons également élaboré 2 considérations cliniques, l’une portant sur l’insuffisance cardiaque et les troubles cognitifs, l’autre, sur le dépistage des déterminants sociaux de la santé défavorables (annexe 3).

Dans l’annexe 4 (accessible en anglais au www.cmaj.ca/lookup/doi/10.1503/cmaj.251137/tab-related-content), les considérations relatives au sexe et au genre pour les recommandations se trouvent au tableau supplémentaire 2; les valeurs et préférences de la patientèle issues du groupe de PEV, au tableau supplémentaire 3; et, finalement, les avantages et les préjudices ainsi que les implications en matière de ressources et de coûts, au tableau supplémentaire 4 (même si aucune analyse en règle des avantages et des préjudices n’a été réalisée).

Le tableau 2 présente le système de classification que nous avons utilisé pour les recommandations. Les recommandations fortes (A) reposent directement sur des données probantes d’un degré de certitude élevé (1A et 1B), et leur formulation stipule « nous recommandons […] ». Les recommandations plus faibles (de B à D) soit reposent sur des données probantes d’un degré de certitude moindre (2 à 4), soit ont nécessité une extrapolation à partir de données probantes d’un degré de certitude élevé, mais qui sont connexes; dans les 2 cas, leur formulation spécifie « nous suggérons […] ». La plupart des données probantes disponibles n’étaient pas explicitement conçues pour évaluer directement des résultats relatifs aux multimorbidités cœur–cerveau, et le degré de certitude des données probantes de la plupart des recommandations a été déclassé en raison de ce manque de données probantes directes^18^.

**Tableau 2:** Classification des données probantes et force de la recommandation, relativement à la prévention ou au traitement^17^

Classification des données probantes	Critères
Degré de certitude 1A	Revue systématique ou méta-analyses d’ECR de grande qualité Recherche exhaustive de données probantes Faible risque de biais dans la sélection des articles inclus Évaluation individuelle de chaque article inclus Conclusions claires appuyées sur des données et des analyses appropriées
Degré de certitude 1B	ECR bien conçus et d’une puissance statistique suffisante pour répondre à la question de recherche Répartition aléatoire de la patientèle dans les divers groupes de traitement Au moins 80 % de suivi Administration des traitements à l’insu de la patientèle et de l’équipe de recherche Analyse des patientes et patients dans les groupes de traitement qu’on leur a assignés Taille de l’échantillon suffisamment grande pour détecter le résultat d’intérêt
Degré de certitude 2A	Données probantes issues d’au moins 1 étude non randomisée, mais contrôlée
Degré de certitude 2B	Données probantes issues d’au moins 1 autre type d’étude quasi expérimentale
Degré de certitude 3	Données probantes issues d’études descriptives non expérimentales, comme les études comparatives, les études de corrélation et les études cas–témoins
Degré de certitude 4	Données probantes issues soit de rapports de comités d’experts, soit d’avis ou d’expériences cliniques provenant d’autorités reconnues, ou les deux
**Force et formulation de la recommandation**	
A — Nous recommandons	Recommandation directement basée sur des données probantes d’un degré de certitude 1
B — Nous suggérons	Recommandation directement basée sur des données probantes d’un degré de certitude 2 ou extrapolée d’une recommandation basée sur des données probantes d’un degré de certitude 1
C — Nous suggérons	Recommandation directement basée sur des données probantes d’un degré de certitude 3 ou extrapolée d’une recommandation basée sur des données probantes d’un degré de certitude 1 ou 2
D — Nous suggérons	Recommandation directement basée sur des données probantes d’un degré de certitude 4 ou extrapolée d’une recommandation basée sur des données probantes d’un degré de certitude 1, 2, ou 3

Remarque : ECR = essai randomisé contrôlé.

### Dépistage

#### Fibrillation auriculaire et risque de troubles cognitifs

*Nous suggérons de soumettre les personnes atteintes de fibrillation auriculaire à un dépistage au moyen d’un instrument validé afin de déceler celles qui sont exposées à un risque de troubles cognitifs (degré de certitude des données probantes 2A, force de la recommandation B).*

De nombreuses études prospectives et rétrospectives ont examiné la relation entre la fibrillation auriculaire d’une part et, d’autre part, les troubles cognitifs et la démence^19^–^22^. Une revue systématique et méta-analyse d’études prospectives (15 études; 2 822 974 personnes) a montré que la fibrillation auriculaire était associée à une augmentation de 39 % du risque de troubles cognitifs dans la population générale (suivi de 3,8 à 25 ans : rapport de risques [HR, pour hazard ratio] de 1,39, intervalle de confiance [IC] à 95 % de 1,25 à 1,53; *I*^2^ = 90,3 %), et à un risque 2,70 fois plus élevé de troubles cognitifs (après un suivi de 0,25 à 3,78 ans : rapport de cotes [RC] corrigé de 2,70, IC à 95 % de 1,66 à 3,74; *I*^2^ = 0,0 %) dans une cohorte post-AVC (6 études; 2009 personnes), après correction en fonction des facteurs de risque de maladie cardiovasculaire dans des analyses multivariées^21^.

Des données évoquent une association entre la fibrillation auriculaire et toutes les formes de troubles cognitifs d’origine vasculaire (toutes causes, maladie d’Alzheimer et démence vasculaire) 23. Fait important, la fibrillation auriculaire peut augmenter le risque de démence à début précoce. Une analyse des données groupées de 6 études a révélé une augmentation du risque de démence à début précoce chez les personnes atteintes de fibrillation auriculaire, avec un risque relatif (RR) global de 1,50 (IC à 95 % de 1,00 à 2,26; *I*^2^ = 57 %) chez les personnes de moins de 70 ans, et de 1,06 (IC à 95 % de 0,55 à 2,06; *I*^2^ = 99,6 %) chez celles de moins de 65 ans^23^. Dans une étude de cohorte prospective populationnelle menée à partir des données de la UK Biobank, un jeune âge au moment du diagnostic de fibrillation auriculaire a été associé à un risque accru de démence toutes causes confondues (HR corrigé pour chaque diminution de 10 ans de 1,23, IC à 95 % de 1,16 à 1,32), de maladie d’Alzheimer (HR corrigé pour chaque diminution de 10 ans de 1,27, IC à 95 % de 1,13 à 1,42) et de démence vasculaire (HR corrigé pour chaque diminution de 10 ans de 1,35, IC à 95 % de 1,20 à 1,51)^24^. Après appariement selon le score de propension, les personnes qui avaient reçu un diagnostic de fibrillation auriculaire avant l’âge de 65 ans étaient exposées au risque le plus élevé de démence toutes causes confondues (HR corrigé de 1,82, IC à 95 % de 1,54 à 2,15), suivies de celles ayant reçu ce diagnostic entre 65 et 74 ans (HR corrigé de 1,47, IC à 95 % de 1,31 à 1,65), et, finalement, de celles ayant reçu le diagnostic à 75 ans ou plus (HR corrigé de 1,11, IC à 95 % de 0,96 à 1,28). Des résultats similaires ont été observés dans le cas de la maladie d’Alzheimer et de la démence vasculaire.

La qualité des données probantes à l’appui de cette recommandation allait de modérée à élevée. Les résultats des grandes études de cohorte prospectives et des méta-analyses étaient cohérents, avec un biais minimal. Dans la méta-analyse portant sur 2,8 millions de personnes^21^, les auteurs spécifiaient que toutes les études incluses répondaient aux critères de l’échelle de Newcastle–Ottawa quant à la représentativité de la cohorte étudiée et à la taille de l’échantillon, et que toutes présentaient un faible risque de biais de détection et de biais d’information. De plus, les graphiques en entonnoir et le test d’Egger n’ont montré aucun biais de publication significatif dans les analyses statistiques. Toutefois, dans l’analyse groupée du risque de démence à début précoce chez les personnes atteintes de fibrillation auriculaire^23^, les auteurs ont signalé une forte hétérogénéité entre les études. Parmi les limites des données existantes, on mentionnait des méthodes et des définitions très variables pour l’établissement des diagnostics de fibrillation auriculaire et de troubles cognitifs. Malgré cela, le sous-comité a souligné l’existence d’un lien fort et indépendant entre la fibrillation auriculaire et les troubles cognitifs^19^–^22^, notamment le fait qu’ils aient en commun les facteurs de risque vasculaires et l’AVC^25^,^26^. Il existe également d’importantes différences selon le sexe, en ce qui concerne le risque de fibrillation auriculaire et son traitement^27^ (annexe 4, tableau supplémentaire 2).

Le dépistage est un moyen simple et sûr de déceler les personnes exposées à un risque élevé, mais la valeur accordée au dépistage des troubles cognitifs varie selon les individus (tableau 1 et annexe 4, tableau supplémentaire 3)^28^,^29^. Les risques associés à ce dépistage comprennent notamment la stigmatisation^28^,^29^, et on ne dispose d’aucun instrument de dépistage spécifique et validé pour la multimorbidité cœur–cerveau.

Même en l’absence d’essais contrôlés randomisés (ECR) démontrant que le dépistage améliore l’issue, le comité de rédaction du guide a estimé que la robustesse et la cohérence des données observationnelles justifiaient la recommandation d’un dépistage systématique des troubles cognitifs chez les personnes atteintes de fibrillation auriculaire, surtout pour permettre une intervention comportementale et médicale précoce. Les données actuelles restent insuffisantes pour déterminer si les traitements recommandés dans les guides de pratique clinique sur la fibrillation auriculaire réduisent le risque de déclin cognitif. La longue durée de suivi nécessaire et la grande taille d’échantillon requise rendent peu probable la réalisation d’ECR pour certains traitements (p. ex., les médicaments pour la maîtrise du rythme cardiaque) visant à démontrer les effets sur le déclin cognitif chez les personnes atteintes de fibrillation auriculaire.

#### Coronaropathie et dépression

*Nous suggérons de soumettre les personnes atteintes de coronaropathie à un dépistage de la dépression au moyen d’un instrument validé (degré de certitude des données probantes 1B, force de la recommandation B).*

Plusieurs études observationnelles ont porté sur la précision des instruments de dépistage de la dépression chez les personnes présentant une coronaropathie. Une revue systématique de 6 études observationnelles incluant au total 1755 personnes atteintes d’un syndrome coronarien aigu a confirmé une sensibilité et une spécificité élevées pour le dépistage au moyen de différents instruments^30^. Selon les études évaluant le Beck Depression Inventory-II (BDI-II), avec un seuil de 14 ou plus, les estimations de sensibilité de cet instrument vont de 83 % à 91 %, et les estimations de spécificité, de 74 % à 88 %. Une méta-analyse de 4 de ces études utilisant ce seuil classique a montré une sensibilité globale de 90 % (IC à 95 % de 86 % à 92 %) et une spécificité de 80 % (IC à 95 % de 68 % à 88 %), avec des valeurs prédictives positives allant de 28 % à 46 % et des valeurs prédictives négatives de 98 % ou 99 %^30^. Selon les études sur l’Échelle d’anxiété et de dépression en milieu hospitalier (EHAD) et ses sous-échelles, aux seuils classiques et optimaux, la précision de cet instrument est plus faible : les estimations de sa sensibilité vont de 41 % à 86 %, et les estimations de sa spécificité, de 62 % à 90 %; ses valeurs prédictives positives se situent entre 23 % et 34 %, et ses valeurs prédictives négatives, entre 93 % et 98 %. Toutefois, l’utilisation de cet instrument était hétérogène, une seule étude ayant utilisé l’EHAD complète. Une dernière étude portant celle-là sur le Questionnaire sur la santé du patient (QSP) — dans ses versions à 2 questions (seuil > 0 point), à 9 questions (seuil > 4 points) et à 10 questions (seuil > 5 points) — a montré l’excellente sensibilité (96 % à 97 %) de cet outil, et une spécificité de 71 % à 78 %.

D’après une revue exploratoire de 51 études, incluant 21 790 personnes, l’EHAD, le BDI-II et le QSP sont les instruments les plus utilisés pour le dépistage de la dépression. Cependant, dans la même étude, on signalait aussi un manque d’uniformité quant à l’intégration de ces instruments dans les modèles de soins et les parcours cliniques en cardiologie, ainsi que dans les guides de pratique clinique sur le syndrome coronarien aigu, en ce qui concerne le dépistage de la dépression ou de l’anxiété et la détermination du meilleur instrument de dépistage. Cette revue a été déclassée, puisqu’il s’agissait d’une revue exploratoire avec synthèse narrative^31^

Des données probantes de qualité modérée à élevée appuient l’utilisation d’instruments de dépistage de la dépression. Dans l’ensemble, les études incluses dans la revue systématique sur le syndrome coronarien aigu^30^ présentaient un faible risque de biais, et démontraient une grande précision dans le dépistage de la dépression. Le QSP s’est révélé l’instrument de dépistage offrant la meilleure sensibilité et la meilleure spécificité, mais les données probantes sur la précision du BDI-II étaient plus abondantes. Même si la santé mentale est une composante importante de la réadaptation cardiovasculaire, le comité de rédaction du présent guide a reconnu l’absence de données probantes issues d’ECR démontrant que le dépistage améliore les résultats. Le groupe de PEV a souligné l’importance de la communication entre les prestataires de soins pour qu’un soutien en santé mentale soit fourni en temps opportun. Selon The Lancet Women and Cardiovascular Disease Commission, la dépression et l’anxiété sont des facteurs de risque de maladie cardiovasculaire sousreconnus chez les femmes (annexe 4, tableau supplémentaire 2)^32^. Le dépistage de la dépression est faisable, efficace et conforme à une approche axée sur la personne; cependant, certaines personnes peuvent craindre la stigmatisation associée au diagnostic de dépression et ne pas souhaiter se plier à un tel dépistage (annexe 4, tableau supplémentaire 3)^33^.

### Traitement

#### Coronaropathie et dépression

*Chez les personnes atteintes de coronaropathie dont le diagnostic de dépression est confirmé, nous recommandons d’entreprendre un traitement par inhibiteur sélectif de la recapture de la sérotonine (ISRS) (degré de certitude des données probantes 1A, force de la recommandation A) ou, selon la gravité de la maladie, nous suggérons un autre traitement fondé sur des données probantes, comme la thérapie cognitivo-comportementale (degré de certitude des données probantes 1B, force de la recommandation C).*

Les données probantes à l’appui de cette recommandation reposaient principalement sur une méta-analyse en réseau de 33 ECR comparant divers traitements de la dépression (pharmacothérapie, psychothérapie, association pharmacothérapie–psychothérapie, exercice, soins collaboratifs) à l’absence de soins ou aux soins usuels chez des personnes atteintes de coronaropathie et de symptômes dépressifs. De ces 33 ECR, 24 fournissaient des estimations de l’efficacité du traitement à l’étude sur la dépression après de 8 à 26 semaines^34^. La plupart des ECR inclus portaient sur des interventions associant la pharmacothérapie et la psychothérapie (p. ex., thérapie interpersonnelle, counseling, thérapie cognitivo-comportementale) ou sur des interventions axées sur l’exercice. En ce qui concerne les interventions pharmacologiques, la classe de médicaments la plus étudiée était celle des ISRS (14 études, 70 %); 10 essais (1935 personnes) comparaient la pharmacothérapie au placebo seul^35^.

Comme des différences moyennes normalisées (DMN) négatives indiquent un meilleur effet du traitement, les données probantes de la méta-analyse en réseau révèlent que les antidépresseurs (principalement des ISRS) sont plus efficaces que le placebo (DMN de −0,28, IC à 95 % de −0,52 à −0,03) et que les soins usuels (DMN de −0,78, IC à 95 % de −1,27 à −0,29), et, possiblement, qu’une combinaison de traitements témoins actifs et inactifs (DMN de −0,33, IC à 95 % de −0,69 à 0,40). À 26 semaines, les résultats de l’étude ont mis en évidence l’efficacité des antidépresseurs par rapport au placebo (DMN de −0,50, IC à 95 % de −0,69 à −0,32), aux soins usuels (DMN de −1,57, IC à 95 % de −2,57 à −0,58) et aux traitements témoins (DMN de −1,43, IC à 95 % de −2,48 à −0,39). Les résultats de la méta-analyse en réseau ont également révélé que la thérapie cognitivo-comportementale réduisait les scores de dépression (DMN de −0,48, IC à 95 % de −0,73 à −0,23). Pour ce qui est des résultats secondaires (variation des différences entre les groupes quant aux symptômes dépressifs, mortalité), les résultats étaient aussi principalement à l’avantage de la pharmacothérapie antidépressive.

Dans d’autres revues systématiques d’essais portant sur diverses combinaisons de traitements, les ISRS ont été jugés sûrs chez les personnes atteintes d’une cardiopathie et ont été associés à des effets bénéfiques sur la dépression, certains éléments évoquant un bienfait cardiaque chez les personnes atteintes de coronaropathie. Dans une revue systématique de 8 ECR (1148 personnes)^36^, les ISRS étaient associés à une réduction significative du risque d’infarctus du myocarde chez les personnes atteintes de coronaropathie et de dépression (RR de 0,54, IC à 95 % de 0,34 à 0,86), ainsi que chez les personnes présentant une dépression après un syndrome coronarien aigu (RR de 0,56, IC à 95 % de 0,35 à 0,90), sans différence statistiquement significative quant à la mortalité toutes causes confondues, à la mortalité cardiovasculaire, aux hospitalisations, à l’angor, à l’insuffisance cardiaque congestive ou à l’incidence des AVC. Une revue exploratoire a montré une absence de différence pour les événements cardiovasculaires entre une prise en charge flexible de la dépression pendant 6 mois par mirtazapine–citalopram et une prise en charge recommandée par un psychiatre à 18 mois (RC de 1,07, IC à 95 % de 0,57 à 2,00)^37^.

Des données probantes dont la qualité va de modérée à élevée montrent que les ISRS diminuent les symptômes dépressifs et des données probantes de qualité modérée appuient l’utilisation de la thérapie cognitivo-comportementale; toutefois, les données probantes démontrant une amélioration directe des résultats cardiovasculaires à la suite du traitement de la dépression sont moins robustes. Dans la méta-analyse en réseau, les évaluations modales du risque de biais étaient faibles pour la plupart des comparaisons^34^. On a décrit les comparaisons entre les différentes formes de psychothérapie et les soins usuels comme soulevant « certaines préoccupations », et on a estimé que les comparaisons entre groupes traités par antidépresseurs et groupes témoins présentaient un risque de biais élevé. Les auteurs ont également signalé une hétérogénéité importante pour l’efficacité à 8 semaines de la psychothérapie par rapport aux soins usuels. Il existe également des différences importantes selon le sexe, quant à l’utilisation et à l’efficacité des ISRS (annexe 4, tableau supplémentaire 2).

Les revues portant sur les bienfaits cardiaques directs ont été déclassées, en raison d’une synthèse narrative des résultats et de l’absence d’évaluation critique. Le sous-comité a souligné l’importance de la santé mentale dans la réadaptation cardiovasculaire. Même si les bienfaits cardiovasculaires directs du traitement de la dépression sont moins clairs, compte tenu de l’amélioration marquée de la qualité de vie associée au traitement de la dépression, à laquelle s’ajoute une tendance à la réduction des événements cardiaques indésirables, le comité de rédaction du guide a jugé que des recommandations fortes en faveur du dépistage et du traitement de la dépression après un syndrome coronarien aigu étaient justifiées. Notons que les patientes et patients diffèrent, quant à la valeur accordée aux diverses modalités de traitement de la dépression (annexe 4, tableau supplémentaire 3)^38^.

#### Hypertension et risque de troubles cognitifs

*Nous recommandons que les personnes dont la pression artérielle (PA) systolique se situe entre 130 mm Hg et 180 mm Hg et qui présentent un risque cardiovasculaire accru (déterminé par la présence d’une maladie cardiovasculaire clinique ou subclinique, d’une maladie rénale chronique, d’un score de Framingham ≥ 15 %, ou d’un âge ≥ 75 ans) reçoivent un traitement intensif visant une PA systolique inférieure à 120 mm Hg afin de réduire le risque de troubles cognitifs (degré de certitude des données probantes 1B, force de la recommandation A).*

Les données probantes à l’appui de cette recommandation reposent sur plusieurs méta-analyses, sur de vastes ECR de grande qualité et sur des études de cohorte prospectives. Les données probantes les plus robustes étaient présentées dans l’analyse groupée des données individuelles des participants et participantes de 5 ECR de la Dementia Risk Reduction (DIRECT) collaboration^39^, incluant 28 008 adultes de 20 pays qui présentaient une PA systolique supérieure à 160 mm Hg ou un facteur de risque (diabète de type 2 ou AVC). Une régression logistique multiniveau a montré un RC corrigé de 0,87 (IC à 95 % de 0,75 à 0,99) en faveur du traitement antihypertenseur pour réduire la probabilité de démence incidente; comme le soulignaient les auteurs, les résultats confirmaient que les données probantes du degré de certitude le plus élevé (degré 1) plaidaient en faveur du traitement antihypertenseur, comparé au placebo.

Dans une autre méta-analyse récente à effets aléatoires^40^ de 14 ECR incluant 96 158 personnes adultes hypertendues (PA moyenne initiale : systolique de 154 mm Hg, écart-type [ÉT] de 14,9 mm Hg; diastolique de 83,3 mm Hg, ÉT de 9,9 mm Hg), 12 de ces études faisaient état d’une démence incidente et 8, d’un déclin cognitif; la réduction de la PA par traitement antihypertenseur était associée de manière significative à une diminution du risque de démence incidente (RC de 0,93, IC à 95 % de 0,88 à 0,98; réduction du risque absolu de 0,39 %, IC à 95 % de 0,09 % à 0,68 %) et de troubles cognitifs (RC de 0,93, IC à 95 % de 0,88 à 0,99; réduction du risque absolu de 0,71 %, IC à 95 % de 0,19 % à 1,2 %), sur une durée moyenne de suivi de 4,1 ans.

Dans l’essai Systolic Blood Pressure Intervention Trial Memory and Cognition in Decreased Hypertension (SPRINT MIND)^41^, 9361 personnes adultes hypertendues se sont vu attribuer au hasard une cible de PA systolique inférieure soit à 120 mm Hg (traitement intensif), soit à 140 mm Hg (traitement usuel), le paramètre d’évaluation principal étant la démence probable confirmée. Sur la durée médiane de suivi (5,11 ans), les résultats n’ont pas montré d’augmentation du risque de démence probable dans les 2 groupes (HR de 0,83, IC à 95 % de 0,67 à 1,04); toutefois, ils ont révélé que la maîtrise intensive de la PA réduisait de manière significative le risque de survenue de troubles cognitifs légers, soit le critère d’évaluation secondaire (HR de 0,81, IC à 95 % de 0,69 à 0,95), ainsi que le taux combiné de troubles cognitifs légers et de démence probable (HR de 0,85, IC à 95 % de 0,74 à 0,97). Dans l’essai China Rural Hypertension Control Project Phase-3 (CRHCP-3)^42^, on a attribué aléatoirement à 14 541 adultes hypertendus soit une cible de PA systolique inférieure à 130 mm Hg et de PA diastolique inférieure à 80 mm Hg, soit le traitement usuel; les nouvelles données issues de cet essai ont aussi montré une réduction du risque de survenue du paramètre d’évaluation principal (démence toutes causes confondues) après un suivi moyen de 48 mois (RR de 0,85, IC à 95 % de 0,76 à 0,95; nombre de personnes à traiter : 123) (ces données n’ont pas été prises en considération pour la recommandation).

Dans l’étude Women’s Health Initiative Memory (WHIMS)^43^ portant sur une grande cohorte prospective de 7207 femmes âgées de 65 à 79 ans, l’incidence du paramètre d’évaluation principal, soit la perte cognitive (troubles cognitifs légers ou démence probable), était de 20,3 (IC à 95 % de 19,3 à 21,3) pour 1000 années-personnes pendant un suivi médian de 9 ans. Les résultats ont montré que les femmes atteintes d’hypertension traitées par antihypertenseurs étaient exposées à un risque accru de perte cognitive (HR de 1,27, IC à 95 % de 1,09 à 1,48) et de perte cognitive légère (HR de 1,35, IC à 95 % de 1,13 à 1,62), comparativement à celles qui avaient une PA normale, avec tests de tendance des HR significatifs (*p* < 0,01).

Des données probantes de qualité modérée à élevée corroborent une réduction du risque de troubles cognitifs chez les personnes recevant un traitement antihypertenseur. Bien que les méta-analyses en réseau aient fourni des données probantes robustes (degré 1), l’essai SPRINT MIND a été interrompu prématurément, et l’ampleur des effets méta-analytiques était cohérente, mais modeste. Le comité de rédaction du guide a tenu compte des données probantes modérées, mais constantes, de la forte préférence de la patientèle pour la préservation de la santé cérébrale et de la faible augmentation du risque absolu d’effets indésirables graves. Compte tenu des doubles bienfaits cardiovasculaires et cognitifs, le groupe a estimé qu’une cible de PA systolique inférieure à 120 mm Hg était justifiée chez les personnes âgées exposées à un risque cardiovasculaire élevé.

#### Dyslipidémie et risque d’AVC ou de maladie cardiovasculaire

*Nous recommandons que les personnes atteintes de coronaropathie dont le taux de cholestérol à lipoprotéines de basse densité (LDL) dépasse le seuil de 1,8 mmol/L reçoivent un traitement intensifié de manière à abaisser le taux de cholestérol LDL en deçà de la cible, afin de prévenir l’AVC et de réduire le risque cardiovasculaire (degré de certitude des données probantes 1A, force de la recommandation A); et nous suggérons que les victimes d’AVC dont le taux de cholestérol LDL dépasse le seuil de 1,8 mmol/L reçoivent un traitement intensifié de manière à abaisser le taux de cholestérol LDL en deçà de la cible, afin de réduire le risque d’événements cardiovasculaires majeurs (degré de certitude des données probantes 1B, force de la recommandation B).*

Plusieurs méta-analyses de grande qualité portant sur de vastes ECR forment un solide corpus de données probantes pour cette recommandation^44^,^45^. En ce qui concerne la réduction du risque d’AVC en présence d’une maladie cardiovasculaire, une métaanalyse de 5 ECR a évalué l’effet d’une pharmacothérapie hypolipidémiante par statines, intensive comparativement à classique, sur le risque d’AVC chez 39 612 personnes présentant un syndrome coronarien aigu; les résultats ont montré une réduction de 14 % du risque d’AVC chez les personnes qui recevaient le traitement par statines intensif, par rapport à celles qui recevaient le traitement classique (RR de 0,86, IC à 95 % de 0,76 à 0,98), en particulier chez celles qui prenaient de l’atorvastatine^44^.

Dans une méta-analyse de 11 essais incluant 24 732 personnes et portant sur l’efficacité et l’innocuité des inhibiteurs de la proprotéine convertase subtilisine/kexine de type 9 (iPCSK9) chez les personnes atteintes d’un syndrome coronarien aigu, les iPCSK9 étaient associés à une réduction significative du risque d’AVC (RC de 0,71, IC à 95 % de 0,52 à 0,98) ainsi qu’à une réduction du risque d’infarctus du myocarde (RC de 0,87, IC à 95 % de 0,78 à 0,97), ce qui a amené les auteurs de cette méta-analyse à recommander les iPCSK9 comme stratégie thérapeutique optimale^45^. Ces médicaments abaissent les taux de cholestérol LDL, de cholestérol total, de triglycérides, de lipoprotéine (a) et d’apolipoprotéine B.

Les essais ODYSSEY OUTCOMES et Further Cardiovascular Outcomes Research with PCSK9 Inhibition in Subjects with Elevated Risk (FOURIER)^46^,^47^, 2 des plus vastes essais sur les iPCSK9, ont démontré que le traitement par iPCSK9 réduisait significativement le risque d’AVC chez les personnes atteintes de maladie cardiovasculaire athéroscléreuse (FOURIER : HR pour l’AVC de 0,79, IC à 95 % de 0,66 à 0,95; ODYSSEY : HR pour l’AVC ischémique mortel ou non mortel de 0,73, IC à 95 % de 0,57 à 0,93). Une métaanalyse de 11 ECR incluant ces essais (la plupart étant des essais de prévention secondaire chez des personnes atteintes de coronaropathie) a montré que l’atteinte d’un taux de cholestérol LDL inférieur à 1,8 mmol/L était associée à une réduction significative du risque d’AVC ischémique (RR 0,77, IC à 95 % de 0,69 à 0,85)^48^.

En ce qui concerne la réduction du risque de maladie cardiovasculaire après un AVC, l’essai Stroke Prevention by Aggressive Reduction in Cholesterol Levels (SPARCL) a évalué les effets de l’atorvastatine à forte dose chez 4731 personnes ayant subi un AVC ou un accident ischémique transitoire de 1 à 6 mois auparavant et présentaient un taux de cholestérol LDL de 2,6 à 4,9 mmol/L, mais aucune coronaropathie connue^49^. On a observé des réductions significatives du risque cardiovasculaire, notamment du risque d’événements coronariens aigus (HR de 0,65, IC à 95 % de 0,50 à 0,84), de la nécessité d’une revascularisation (HR de 0,55, IC à 95 % de 0,43 à 0,72) et de l’infarctus du myocarde non mortel (HR de 0,51, IC à 95 % de 0,35 à 0,74). Le taux de cholestérol LDL moyen atteint dans le groupe traité par statines était d’environ 1,9 mmol/L. Une analyse a posteriori de l’essai SPARCL a démontré que les personnes qui avaient atteint un taux de cholestérol LDL inférieur à 1,8 mmol/L étaient exposées à un risque significativement plus faible d’événements coronariens majeurs que celles dont le taux demeurait à 2,6 mmol/L ou plus (HR de 0,64, IC à 95 % de 0,45 à 0,91)^50^.

Enfin, dans l’essai en parallèle Treat Stroke to Target, on a réparti au hasard 2860 personnes ayant subi un AVC ischémique dans les 3 mois précédents ou un accident ischémique transitoire dans les 15 jours précédents pour qu’elles atteignent un taux cible de cholestérol LDL soit inférieur à 1,8 mmol/L (groupe de la cible basse), soit de 2,3 à 2,8 mmol/L (groupe de la cible élevée)^51^. Le paramètre d’évaluation principal composite des événements cardiovasculaires majeurs comprenait l’AVC ischémique, l’infarctus du myocarde, l’apparition de nouveaux symptômes menant à une revascularisation coronarienne ou carotidienne urgente et le décès d’origine cardiovasculaire. Le taux de cholestérol LDL moyen initial était de 3,5 mmol/L; le taux de cholestérol LDL moyen atteint était de 1,7 mmol/L dans le groupe de la cible basse et de 2,5 mmol/L dans le groupe de la cible élevée. Les personnes à qui on avait attribué un taux cible de cholestérol LDL inférieur à 1,8 mmol/L étaient exposées à un risque moindre pour ce qui est du paramètre d’évaluation principal composite d’événements cardiovasculaires majeurs subséquents (HR de 0,78, IC à 95 % de 0,61 à 0,98)^51^.

Les données probantes en faveur du traitement hypolipidémiant pour réduire le risque d’AVC et de maladie cardiovasculaire sont robustes. De vastes méta-analyses et ECR corroborent des réductions significatives du risque dans divers groupes de personnes, une autre méta-analyse ne faisant état d’aucune hétérogénéité significative entre les essais inclus (*I*^2^ = 0 %; *p* = 0,74)^44^. Le comité de rédaction du guide a reconnu que le traitement hypolipidémiant procure une double protection manifeste contre les événements cardiaques et cérébraux. La robustesse des données des essais randomisés montrant une réduction des AVC et des événements coronariens lorsque le taux de cholestérol LDL est inférieur à 1,8 mmol/L justifie l’harmonisation de la prise en charge de la maladie cardiovasculaire et de la maladie cérébrovasculaire. Ce bienfait est particulièrement important compte tenu des conséquences dévastatrices de l’AVC sur les plans personnel et sociétal. Notons que, dans l’ensemble des essais inclus dans la méta-analyse de Xie et ses collègues^44^, la proportion d’hommes variait de 75 % à 83 %, ce qui met en lumière la forte sous-représentation des femmes dans les essais sur les traitements hypolipidémiants et dans les données probantes ayant servi à formuler cette recommandation (annexe 4, tableau supplémentaire 2).

#### Vaccination et risque d’AVC, de décès d’origine cardiovasculaire, d’infarctus du myocarde ou de démence

*Nous suggérons d’offrir systématiquement à toutes les personnes, et en particulier à celles de plus de 65 ans, la vaccination contre la grippe afin de réduire le risque de décès d’origine cardiovasculaire et d’AVC (degré de certitude des données probantes 2A; force de la recommandation C), ainsi que, possiblement, le risque de démence (degré de certitude des données probantes 3, force de la recommandation C); la vaccination contre les infections à pneumocoque afin de réduire le risque d’infarctus du myocarde et d’AVC (degré de certitude des données probantes 2A, force de la recommandation B); et la vaccination contre le zona afin de réduire le risque d’infarctus du myocarde et d’AVC (degré de certitude des données probantes 2A, force de la recommandation B), ainsi que, possiblement, le risque de démence (degré de certitude des données probantes 3, force de la recommandation C).*

Les recommandations relatives à la vaccination contre la grippe et contre les infections à pneumocoque reposaient principalement sur des méta-analyses de données issues d’ECR; concernant le vaccin contre le zona et les résultats liés à la démence, seules des données observationnelles étaient disponibles, ce qui a entraîné un déclassement de ces recommandations.

Une méta-analyse de grande qualité portant sur 6 ECR^52^ et incluant au total 9001 personnes (âge moyen de 65,5 ans) a montré que la vaccination contre la grippe était associée à une réduction du risque d’événements cardiovasculaires composites (RR de 0,66, IC à 95 % de 0,53 à 0,83) et que, concernant le décès d’origine cardiovasculaire dans les 12 mois de suivi, on a observé une interaction liée au traitement selon que les personnes avaient connu un syndrome coronarien aigu récent (RR de 0,44, IC à 95 % de 0,23 à 0,85) ou non (RR de 1,45, IC à 95 % de 0,84 à 2,50). Dans une autre méta-analyse récente de 15 essais incluant 745 001 personnes au total^53^, on a observé de plus faibles taux de mortalité toutes causes confondues (RC de 0,74, IC à 95 % de 0,64 à 0,86), de mortalité cardiovasculaire (RC de 0,73, IC à 95 % de 0,59 à 0,92) et d’AVC (RC 0,71, IC à 95 % de 0,57 à 0,89) chez les personnes qui avaient reçu le vaccin contre la grippe plutôt que le placebo. Enfin, les données les plus récentes montrent que, chez les personnes exposées à un risque élevé d’événements cardiovasculaires et qui étaient vaccinées, le risque de décès d’origine cardiovasculaire diminuait significativement, soit de 23 % à 47 %; le risque d’événements cardiaques indésirables majeurs diminuait de 26 % à 37 %, le risque d’infarctus du myocarde, de 29 % à 34 % et le risque d’AVC, de 13 % à 19 %, par rapport à celles qui n’étaient pas vaccinées^54^.

Deux grandes méta-analyses (la plus récente, menée en 2020, portait sur 11 études incluant 332 367 personnes; l’autre, réalisée en 2015, portait sur 12 études incluant 716 108 personnes)^55^,^56^ ont fait état des bienfaits de la vaccination contre les infections à pneumocoque sur le risque d’infarctus du myocarde et d’AVC. Dans la méta-analyse de 2015, les RR des données groupées étaient de 0,86 (IC à 95 % de 0,76 à 0,97) pour les événements cardiovasculaires et de 0,92 (IC à 95 % de 0,86 à 0,98) pour la mortalité cardiovasculaire, cet effet protecteur s’atténuant dans les 2 cas après 1 an (RR de 1,03, IC à 95 % de 0,93 à 1,14)^56^. En ce qui concerne l’infarctus du myocarde et l’AVC précisément, le rôle protecteur de la vaccination contre les infections à pneumocoque était statistiquement significatif uniquement chez les adultes (8 des 11 études, âge moyen > 60 ans; RR de 0,90, IC à 95 % de 0,817 à 0,999 pour les événements cardiovasculaires; RR de 0,86, IC à 95 % de 0,75 à 0,99 pour la mortalité cardiovasculaire). Dans la méta-analyse de 2020, la vaccination par le vaccin polysaccharidique pneumococcique (PPSV23) réduisait le risque d’infarctus du myocarde (RR de 0,88, IC à 95 % de 0,79 à 0,98) et de mortalité toutes causes confondues (RR de 0,78, IC à 95 % de 0,68 à 0,88), avec des effets significatifs chez les personnes de 65 ans ou plus, mais ne réduisait pas le risque d’AVC de manière significative^55^. Allant dans le sens de ces méta-analyses de données d’essais, une vaste et récente étude cas–témoins menée à l’intérieur d’une cohorte populationnelle (incluant 383 781 personnes âgées de 65 ans ou plus)^57^ a aussi fait état d’une diminution des risques d’infarctus du myocarde aigu (RC de 0,70, IC à 95 % de 0,62 à 0,80) et d’AVC (RC de 0,81, IC à 95 % de 0,77 à 0,86).

Dans une méta-analyse de 12 études observationnelles, dont 6 sur l’évaluation du vaccin contre le zona, la vaccination contre le zona a été associée à une plus faible probabilité d’AVC (RC de 0,78, IC à 95 % de 0,68 à 0,90)^58^. Dans une vaste et récente étude rétrospective cas–témoins avec appariement incluant 27 093 personnes adultes atteintes de maladies concomitantes qui les exposaient à un risque, la vaccination contre le zona était associée à une diminution du risque d’AVC et d’infarctus du myocarde chez les personnes adultes atteintes de diabète (AVC : RC de 0,64, IC à 95 % de 0,58 à 0,71; infarctus du myocarde : RC de 0,63, IC à 95 % de 0,57 à 0,71), d’hypertension (AVC : RC de 0,75, IC à 95 % de 0,68 à 0,83; infarctus du myocarde : RC de 0,73, IC à 95 % de 0,65 à 0,81) ou de maladie pulmonaire obstructive chronique (AVC : RC de 0,75, IC à 95 % de 0,68 à 0,83; infarctus du myocarde : RC de 0,74, IC à 95 % de 0,66 à 0,83)^59^.

Lors de nos recherches initiales, les résultats relatifs à la démence associés à la vaccination reposaient principalement sur des données observationnelles issues du Clinical Practice Research Datalink au Royaume-Uni^60^. Dans cette étude observationnelle, on avait constitué 2 cohortes appariées, soit 854 745 personnes exposées au vaccin contre le zona appariées à 8,8 millions de comparateurs, et 742 487 personnes exposées au vaccin contre la grippe appariées à 7,12 millions de comparateurs. Les résultats ont montré que la vaccination contre le zona était associée à un risque plus faible de diagnostic de démence (HR corrigé de 0,78, IC à 95 % de 0,77 à 0,79) et de maladie d’Alzheimer (HR corrigé de 0,91, IC à 95 % de 0,89 à 0,92), et que la vaccination contre la grippe réduisait le risque de démence (HR de 0,96, IC à 95 % de 0,94 à 0,97). Des méta-analyses plus récentes confirment que la vaccination contre le zona et contre la grippe réduit le risque de démence^61^–^63^. Les études incluses dans ces méta-analyses étendent aussi cette observation à la vaccination contre les infections à pneumocoque.

La qualité des données probantes allait de modérée à élevée pour la vaccination contre la grippe et contre les infections à pneumocoque, en ce qui concerne la protection cardiovasculaire; elle était modérée pour la vaccination contre le zona, mais nous ne disposions d’aucune donnée d’essai pour cette dernière. Les données probantes quant aux résultats relatifs à la démence ont aussi été déclassées, parce qu’on ne disposait que de données observationnelles. Le sous-comité a souligné la robustesse et l’abondance croissante des données probantes à l’appui de la vaccination comme moyen de prévenir les complications cardiovasculaires et cérébrovasculaires. Compte tenu des rapports risques–bienfaits et coût–efficacité favorables et de la pertinence de cette intervention pour un vaste éventail de personnes, le comité de rédaction du guide a estimé qu’il était justifié d’offrir systématiquement ces vaccins aux personnes âgées et aux populations à risque élevé dans le cadre des stratégies de promotion de la santé cœur–cerveau. Nous n’avons pas trouvé suffisamment de données sur la vaccination contre le virus respiratoire syncytial et contre la COVID-19 pour émettre des recommandations sur ces sujets.

### Prise de décision partagée

*Nous recommandons que les cliniciennes et cliniciens encouragent les personnes atteintes d’affections du cœur et du cerveau, ou qui risquent d’en être atteintes, à participer activement à la prise de décision concernant leurs soins, et cela en utilisant avant et pendant les consultations des outils d’aide à la décision fondés sur des données probantes, afin de faciliter la prise de décision partagée (degré de certitude des données probantes 1A, force de la recommandation B).*

Les outils de transfert des connaissances fondés sur des données probantes sont conçus pour aider la patientèle à faire des choix précis et réfléchis entre diverses options de soins; ils complètent, sans les remplacer, les conseils des cliniciennes et cliniciens quant aux options thérapeutiques. Selon l’International Patient Decision Aid Standards Collaboration^64^, les outils d’aide à la décision destinés à la patientèle doivent au minimum répondre aux critères suivants : énoncer explicitement la décision à envisager pour la population ciblée; fournir une information équilibrée et fondée sur des données probantes sur le problème de santé, les options à considérer, de même que les bienfaits et les préjudices associés à chacune; et aider la patientèle à préciser implicitement ou explicitement la valeur qu’elle accorde aux bienfaits et aux préjudices de chaque option.

Les données probantes à l’appui de cette recommandation reposaient sur une revue systématique de grande qualité portant sur 209 ECR qui comparaient les soins usuels à des soins avec recours à des outils d’aide à la décision^65^. Les résultats ont montré que ces outils augmentaient les connaissances de la patientèle (degré de certitude des données probantes élevé), amélioraient l’exactitude de la perception des risques (données probantes de certitude élevée), augmentaient la participation de la patientèle à la prise de décision (données probantes d’un degré de certitude élevé), contribuaient à la clarification des valeurs (données probantes de certitude élevée) et amélioraient probablement la concordance entre les choix et les valeurs mises en évidence (données probantes de certitude modérée).

Le degré de certitude des données probantes à l’appui des bienfaits des outils d’aide à la décision était élevé. En tenant compte de la robustesse des données probantes selon lesquelles ces outils améliorent le processus décisionnel sans effets néfastes, le sous-comité a accordé la priorité à l’utilisation des outils d’aide à la décision. Étant donné les arbitrages complexes inhérents à la prise en charge de la santé cœur–cerveau (p. ex., quant aux stratégies de prévention de l’AVC, aux décisions relatives à la vaccination ou aux interventions liées au mode de vie), il est crucial de faciliter la prise de décisions éclairées et cohérentes avec les valeurs de la personne. L’intégration des outils d’aide à la décision dans les soins cliniques favorise l’autonomisation de la patientèle, une meilleure concordance avec ses préférences et, possiblement, une plus forte observance à long terme des traitements choisis. Des travaux sont en cours afin de recenser des outils d’aide à la décision accessibles au public.

## Méthodes

La méthodologie détaillée du présent guide de pratique clinique est décrite dans un article sur son protocole^18^. En bref, notre guide a été élaboré par un comité de rédaction réunissant un groupe diversifié d’experts et d’expertes de nombreuses disciplines, dont au moins la moitié étaient des prestataires de soins primaires pour les utilisatrices et utilisateurs finaux cibles (annexe 5, tableau supplémentaire 5, accessible en anglais au www.cmaj.ca/lookup/doi/10.1503/cmaj.251137/tab-related-content). L’élaboration du guide a suivi les principes de l’outil Appraisal of Guidelines for Research and Evaluation II (AGREE II), validé pour l’élaboration et la présentation des guides de pratique clinique^66^. L’évaluation des recommandations a été réalisée selon les critères de classification décrits par Shekelle et ses collègues^67^, critères qui conjuguent le consensus des personnes expertes et le contexte aux données probantes disponibles (tableau 2).

L’élaboration du guide a été financée par le programme Interconnectome Cœur–Cerveau (ICC) de l’Université d’Ottawa (https://www.uottawa.ca/recherche-innovation/icc), soutenu par le Fonds d’excellence en recherche Apogée Canada.

### Composition des groupes participants

Composé de membres de C-CHANGE (P.L., S.W.T., R.J., J.D.E., D.H-S.) auxquels s’est ajouté un expert en santé cœur–cerveau (J.D.E.), le comité directeur du présent guide de pratique clinique a supervisé la planification, l’utilisation des ressources, les échéanciers, la constitution des comités et la mise en œuvre, tout en assurant la transparence des intérêts concurrents durant l’élaboration des recommandations et les votes.

Dix sous-comités de personnes expertes (annexe 2, figure supplémentaire 1, et annexe 5, tableau supplémentaire 5) ont été constitués en tenant compte de l’expertise des membres dans l’élaboration de guides de pratique clinique et dans les domaines concernés, de même que de la représentation des utilisatrices et utilisateurs finaux cibles (annexe 5, tableau supplémentaire 5). Chacun des sous-comités comprenait 1 ou 2 personnes à la présidence et 4 ou 5 autres expertes et experts. Les sous-comités portaient respectivement sur les maladies cœur–cerveau et le diabète, les maladies cœur–cerveau et l’hypertension, les maladies cœur–cerveau et la dyslipidémie, les maladies cardiaques et la dépression, l’insuffisance cardiaque et la démence, la fibrillation auriculaire et la démence, la vaccination pour prévenir les événements cardiaques et les AVC, les interventions comportementales, la prise de décision partagée et le soutien à la décision, et la responsabilité sociale.

L’équipe d’analyse des données probantes comprenait 8 membres (2 coprésidentes [J.D.E., Z.L.] et 6 expertes et experts [M.H., D.H-S., M.R., C.A.G., M.L., S.M.] en épidémiologie, en biostatistique, en méthodes de recherche et en évaluation des données probantes) afin de maintenir la cohérence et la rigueur tout au long du processus d’élaboration du guide; une personne membre de cette équipe a été affectée à chacun des sous-comités. De plus, nous avons sollicité l’appui de la McMaster Evidence Review and Synthesis Team pour soutenir les recherches documentaires et l’évaluation critique.

Pour que les recommandations du présent guide de pratique clinique reposent sur une expérience concrète et soient applicables aux personnes directement concernées par le guide, le comité directeur a également invité des PEV (y compris des proches aidantes et aidants) à se joindre au groupe traitant de leur maladie afin de contribuer à ses travaux dès le départ. Les PEV ont été recrutées par la Fondation des maladies du cœur et de l’AVC — à même son propre groupe de PEV — et au sein de la patientèle des membres du comité directeur. Comme le voulait notre stratégie de collaboration du présent guide, le groupe des 12 PEV a fait part au comité directeur de ses commentaires et points de vue sur la sélection des thèmes abordés par les recommandations et d’autres questions à l’étude. Puis, lors de l’extraction et de l’évaluation critique des données probantes, le groupe de PEV a fourni ses réactions sur les questions soulevées par les sous-comités. Enfin, le groupe de PEV a été invité à assister et à participer à la conférence de consensus, à examiner et à commenter les projets de recommandations issus de cette conférence avant leur ratification, et à réviser le manuscrit préliminaire du guide avant sa soumission. Avec la permission des membres de ce groupe de PEV, nous les nommons et les remercions dans le présent manuscrit.

Le rôle du comité de mise en œuvre (K.K., N.H., P.H.J., S.V.P.) consistait à faciliter l’intégration des recommandations pour la pratique clinique dans les dossiers de santé électroniques en s’appuyant sur le cadre SMART, et à favoriser leur adoption par les cliniciennes et cliniciens (annexe 1, tableau supplémentaire 1)^18^.

Le comité de rédaction du guide (59 membres) englobait le comité directeur, les sous-comités, l’équipe d’analyse des données probantes et le comité de mise en œuvre.

### Sélection des thèmes prioritaires

Le comité directeur a élaboré une liste de thèmes potentiels relatifs à la multimorbidité cœur–cerveau et, avec la McMaster Evidence Review and Synthesis Team, a réalisé les recherches dans la documentation pour le guide ainsi qu’une première analyse documentaire afin de déterminer s’il existait des données probantes suffisantes pour émettre une recommandation sur chacun des thèmes. Avant la formation des sous-comités, les thèmes ont été examinés par les membres actuels de C-CHANGE, l’équipe d’analyse des données probantes, le comité directeur et le groupe de PEV. Les thèmes pertinents déjà abordés dans les guides de C-CHANGE^11^ et de la Société cardiovasculaire du Canada (https://ccs.ca/fr/) ont été exclus.

Par consensus, nous avons sélectionné les thèmes définitifs et constitué 10 sous-comités d’experts et d’expertes. Notons que les recommandations finalement retenues n’incluent pas celles qui provenaient des sous-comités « interventions comportementales » et « maladies cœur–cerveau et diabète »; toutefois, nous reconnaissons l’importance des changements comportementaux pour la santé cœur–cerveau, et nous soulignons l’existence d’autres guides, portant notamment sur le diabète de type 2, qui se concentrent sur les interventions comportementales^68^.

### Revue de la documentation et évaluation de la qualité

Chaque sous-comité s’est réuni virtuellement afin d’élaborer un cadre PICO (population, intervention, comparaison, résultat [outcome]) pour son thème. Nous avons demandé au McMaster Evidence Review and Synthesis Team de réaliser une recherche systématique de la documentation et une analyse documentaire, et de participer à l’évaluation critique pour chacun des cadres PICO (annexe 6, accessible en anglais au www.cmaj.ca/lookup/doi/10.1503/cmaj.251137/tab-related-content). Une bibliothécaire spécialisée en sciences de la santé formée sur Cochrane a réalisé une recherche documentaire exhaustive dans 3 bases de données (MEDLINE, Cochrane Library et Embase). Les recherches ont eu lieu de mai à août 2024. Chaque étude répertoriée a fait l’objet d’une évaluation critique par des membres de l’équipe d’analyse des données probantes à l’aide d’outils normalisés pour les études interventionnelles ou observationnelles afin de déterminer le degré de certitude des données probantes, et cela en tenant compte de leur pertinence par rapport à la question clinique, de la qualité méthodologique, du risque de biais et de la généralisabilité.

La méthode de classification des données probantes utilisée dans ce guide (tableau 2) est celle conçue et élaborée par Shekelle et ses collègues, et elle a déjà été décrite^18^,^67^. Nous avons choisi cette méthode précisément parce qu’elle ajoute aux données probantes disponibles le consensus de personnes expertes et le contexte, ce qui est particulièrement important en présence de données probantes limitées ou de plans d’étude hétérogènes. La méthode de Shekelle donne la priorité à la validité externe plutôt qu’à la validité interne, ce qui est utile dans le contexte de la multimorbidité, comme c’est le cas dans d’autres guides récents sur des affections multimorbides^69^. Ce système évalue la qualité des données probantes selon 4 degrés de certitude, le plus élevé étant réservé aux données probantes issues de revues systématiques, de méta-analyses d’ECR ou d’ECR adéquatement conçus et d’une puissance statistique satisfaisante.

Plus précisément, nous avons procédé à une évaluation critique de chaque étude selon son plan à l’aide d’outils méthodologiques validés et fiables (comme des outils d’évaluation du risque de biais). Toutefois, conformément à la méthode de classification Grading of Recommendations Assessment, Development and Evaluation (GRADE)^70^,^71^, nous avons abaissé le degré de certitude de certaines données probantes pour diverses raisons, notamment des limites liées au plan de l’étude, un risque de biais et le recours à des plans d’étude autres que celui des ECR (tableau 2). Comme le prévoit le processus de C-CHANGE, nous avons accordé la priorité à la qualité de l’ensemble des données probantes pour déterminer la force d’une recommandation, tout en reconnaissant que d’autres facteurs peuvent également contribuer à cette force, notamment les valeurs et les préférences de la patientèle^16^.

### Élaboration des recommandations

Le calendrier du processus d’extraction des données et d’élaboration des recommandations se trouve à l’annexe 2 (figure supplémentaire 2), laquelle montre la correspondance entre notre processus et les domaines pertinents ainsi que les recommandations de l’outil AGREE II^66^.

Une fois l’évaluation critique réalisée dans DistillerSR, l’équipe d’analyse des données probantes a fourni un rapport d’évaluation critique à chaque sous-comité en appui à la rédaction préliminaire des recommandations. La classification de chaque recommandation dépendait du degré de certitude et de l’ensemble des données probantes relatives à la recommandation, ainsi que de sa concordance avec les données probantes citées (tableau 1). L’annexe 6 (tableaux supplémentaires 6 à 13) présente le cadre PICO de chacun des sous-comités, ses stratégies de recherche et ses tableaux de données probantes.

En novembre 2024, nous avons tenu une conférence pour discuter des recommandations préliminaires de chaque souscomité. Nous avons adopté la méthodologie de consensus clinique de l’American College of Obstetricians and Gynecologists^72^ pour parvenir à un consensus. Les recommandations préliminaires rédigées par chaque sous-comité devaient être approuvées par l’ensemble du sous-comité et par l’équipe d’analyse des données probantes avant d’être discutées lors de la conférence.

La ou le membre de l’équipe d’analyse des données probantes qui avait travaillé étroitement avec un sous-comité présentait la recommandation préliminaire de ce dernier, ainsi que son cadre PICO, la documentation pertinente, le degré de certitude des données probantes, concernant notamment les bienfaits et les préjudices associés à la recommandation, les valeurs et préférences de la patientèle et, le cas échéant, les considérations relatives au sexe et au genre, et les implications en matière de ressources. Cette façon de procéder assurait la présentation cohérente et complète de chacune des recommandations.

Un quorum des deux tiers des membres présents du comité de rédaction devait être atteint pour qu’un vote ait lieu, et toute personne membre ayant un conflit d’intérêts l’empêchant de voter sur un thème donné était soustraite du nombre total de votantes et votants admissibles. Après une discussion approfondie de chaque recommandation et des données probantes qui l’appuyaient, nous avons tenu un vote pour approuver la recommandation parmi les personnes présentes; un seuil d’approbation de 70 % des voix était requis pour faire adopter la recommandation^73^. Les recommandations n’ayant pas atteint ce seuil ont été réexaminées lors de discussions supplémentaires, puis soumises à un second vote avec le même seuil d’approbation.

Durant le processus d’élaboration du guide, certaines données probantes présentées au moment de la conférence n’étaient pas suffisantes soit pour émettre une recommandation (dans le cas de l’intervention comportementale), soit pour atteindre le seuil d’approbation permettant l’inclusion dans les recommandations finales (maladies cœur–cerveau et diabète)^18^.

Toutes les recommandations proposées qui ont atteint le seuil d’approbation lors du premier ou du second vote ont été transmises à l’ensemble du comité de rédaction du guide pour un vote de ratification tenu 2 semaines après la conférence. Afin que les recommandations de notre guide de pratique clinique aident les prestataires de soins à offrir les meilleurs soins possibles, les recommandations préliminaires ont été soumises au groupe de PEV et au comité de mise en œuvre avant leur ratification par l’ensemble du groupe de rédaction. Le groupe de PEV a formulé explicitement les préférences et les valeurs des groupes de patients et patientes, et a apporté des précisions contextuelles supplémentaires pour affiner les recommandations et en établir la priorité. Pour sa part, le comité de mise en œuvre a fourni des conseils sur la formulation des recommandations afin de faciliter leur intégration dans les dossiers de santé électroniques et leur utilisation par les cliniciennes et cliniciens.

### Gestion des intérêts concurrents

Le présent guide s’appuie sur les principes du Guidelines International Network (GIN) relatifs à la divulgation des conflits d’intérêts^74^ afin que ceux-ci soient déclarés en toute transparence, que les membres en conflit d’intérêts soient minoritaires et qu’on les exclue du vote lorsque cela semblait approprié. Les mécanismes de soutien comprenaient un tableau de bord en ligne pour assurer une gestion active des conflits d’intérêts tout au long du processus, ainsi que des mesures pour empêcher tout vote accidentel. Chacun des sous-comités était présidé par une personne experte ayant peu ou pas d’intérêts concurrents liés au thème traité, avec la participation d’une personne de l’équipe d’analyse des données probantes qui n’avait aucun intérêt concurrent. Chaque membre du comité de rédaction du guide devait remplir le formulaire normalisé de l’International Committee of Medical Journal Editors au moment de la soumission des cadres PICO par les sous-comités, pour déclarer toutes les relations, activités et intérêts liés à l’élaboration du présent guide de pratique clinique, et devait mettre ce formulaire à jour avant la conférence de consensus ainsi qu’avant la publication du guide.

Le bailleur de fonds, soit le programme ICC de l’Université d’Ottawa, n’a exercé aucune influence sur le contenu, l’élaboration ou les recommandations du présent guide. Bien que plusieurs membres du comité de rédaction (P.L., Sm.P., M.H., T.V., K.L., D.S., C.G., M.L.) soient des chercheuses ou chercheurs et expertes ou experts cliniques affiliés à ce programme, ils et elles avaient été invités à participer en raison de leur expertise et ne représentaient pas le programme ICC.

## Mise en œuvre

La mise en œuvre de ce guide sera facilitée par le volet de transfert des connaissances de C-CHANGE, CHEP+, un organisme à but non lucratif regroupant des médecins, ainsi que par une publication en libre accès. Pour favoriser l’intégration des recommandations dans la pratique clinique, les processus de transfert des connaissances comprennent des programmes agréés de formation professionnelle continue proposant des activités d’apprentissage de groupe, tant en personne qu’en ligne. Le guide sera mis en vedette à la conférence nationale de mise à jour des guides cliniques C-CHANGE de 2026, lors de présentations en plénière et d’ateliers interactifs basés sur des cas — des activités agréées par le Collège des médecins de famille du Canada et le Collège royal des médecins et chirurgiens du Canada, conçues pour aider les praticiens et praticiennes à modifier leurs pratiques cliniques. Des activités d’apprentissage individuel à domicile, elles aussi agréées, sont accessibles en ligne sur le site de CHEP+ Learning Series @Home (www.chepplus.com, www.cchangeguidelines.com et www.learningstore.chepplus.com; tous en anglais). Nous travaillons avec le Groupe de recherche sur les outils d’aide à la décision pour les patients de l’Université d’Ottawa (https://decisionaid.ohri.ca/francais/index.html) pour améliorer la mise en œuvre par l’utilisation d’outils d’aide à la décision.

À l’échelle des provinces, C-CHANGE collabore étroitement avec les ministères de la Santé afin de promouvoir des programmes régionaux et de proposer des ateliers lors de rencontres nationales et provinciales en soins primaires — ce qui comprend des stratégies axées sur l’équité, notamment l’engagement auprès des collectivités des Premières Nations, des Métis et des Inuits du Canada.

L’élaboration du présent guide a été commanditée par le programme ICC, financé par le gouvernement fédéral, et ce programme prévoit des mises à jour bisannuelles. Nous mettrons ce guide à jour régulièrement, le prochain cycle devant débuter en 2027. Une surveillance et une évaluation continues permettront de mesurer l’adoption des recommandations du guide, de cerner les obstacles à leur mise en œuvre et d’orienter ces mises à jour.

## Autres guides

Aucun autre guide n’est directement comparable au présent guide, puisque celui-ci intègre des données issues à la fois de la documentation en neurologie et en cardiologie pour orienter la prise en charge conjointe de la multimorbidité cœur–cerveau. Cependant, d’autres guides sont pertinents, notamment les guides récents de l’American Heart Association/American College of Cardiology (ACC) et de la European Society of Cardiology sur la prise en charge de l’hypertension artérielle^75^,^76^, le consensus d’experts de l’ACC quant au processus décisionnel pour le traitement de l’insuffisance^77^, ainsi que le parcours clinique HEARTS 2.0 pour l’intégration de l’hypertension et de la multimorbidité cardiovasculaire-rénale-métabolique^78^.

## Lacunes des connaissances

Les principales lacunes dans la documentation relevées lors de l’élaboration du présent guide concernaient spécifiquement les analyses selon le sexe et le genre dans les études examinées. Comme nous l’avons souligné pour plusieurs recommandations, le recrutement de participantes était insuffisant dans un certain nombre d’essais, ce qui limite la généralisabilité des recommandations à la population féminine. On constate également dans les études un manque de renseignements relatifs au sexe et au genre, et nous invitons les équipes de recherche à intégrer des analyses fondées sur le sexe et le genre dans leurs publications. Autre lacune, la documentation utilisée pour élaborer le présent guide ne portait pas sur des évaluations directes et explicites de paramètres à la fois cérébraux et cardiaques, ce qui a entraîné un déclassement des données probantes pour la plupart des recommandations. Des recherches supplémentaires sont nécessaires pour élaborer des paramètres d’évaluation qui conviennent aux populations touchées par la multimorbidité.

## Limites

Le présent guide est limité par l’étendue de la documentation publiée sur la multimorbidité cœur–cerveau. Comme nous l’avons mentionné, une grande partie des études incluses n’étaient pas conçues pour évaluer directement des paramètres relatifs à la multimorbidité cœur–cerveau, ce qui a entraîné un déclassement des données probantes pour la plupart des recommandations. De plus, le champ d’application du présent guide de pratique clinique est restreint et, compte tenu du vaste spectre de la multimorbidité cœur–cerveau, ce guide ne présente pas un ensemble exhaustif de recommandations en la matière. Nous avons plutôt ciblé les maladies cœur–cerveau concomitantes sur lesquelles les données probantes étaient les plus robustes. D’autres affections cœur–cerveau pourraient être intégrées lors du prochain cycle d’élaboration de notre guide de pratique clinique.

## Conclusion

L’existence du présent guide de pratique clinique repose sur la compréhension croissante de l’importance des liens entre les maladies du cœur et celles du cerveau en tant que cause majeure de multimorbidité chez les populations vieillissantes. Les principales recommandations de ce guide de pratique clinique comprennent notamment le dépistage du déclin cognitif en cas de fibrillation auriculaire et de la dépression en cas de coronaropathie; le traitement de la dépression en cas de coronaropathie, des troubles cognitifs en cas d’hypertension, et de la dyslipidémie en cas d’AVC; et la vaccination pour prévenir l’AVC, l’infarctus du myocarde et la démence. Nous recommandons également la prise de décision partagée. Nous avons cherché à produire un guide concret et applicable, principalement destiné aux prestataires de soins primaires, mais qui peut également faciliter la prestation et l’individualisation des soins de surspécialité, et soutenir les équipes interprofessionnelles qui prennent en charge une patientèle aux prises avec la multimorbidité cœur–cerveau.

## Supplementary Information












