# Supplementary material for: Prise en charge de la multimorbidité cœur–cerveau : un guide de pratique clinique
Source: CMAJ. 2026 May 25;198(20):E784–801. [Article in French] doi: 10.1503/cmaj.251137-f (PMC13218600; doi:10.1503/cmaj.251137-f)
Supplement: Supplementary file 1 [file 251137-guide-1-at.pdf]

Appendix 1. Supplemental Table 1. **SMART Clinical Practice Guideline Recommendations**

	**Description**
**S**	Strength of recommendation.<br>e.g., Strong – 'We recommend'; Weak – 'We suggest'
**M**	Measurable at the individual level.<br>e.g., Blood pressure control of <130/80
**A**	Actionable. If the test or treatment is strongly recommended but not yet available, this recommendation should be seen as a new 'policy' from guideline developers with the expectation that the health care system will find a way to make it part of usual practice.
**R**	Record friendly. Recommendations are computable and trackable in electronic medical record systems using quantitative markers; actionable recommendations should be easily displayed. Timings should be computable.<br>E.g., Reassess in 3-months, as opposed to 'when stable' or 'when appropriate'
**T**	Time frame.<br>e.g., When titrating antihypertensives, assess the patient within 8 weeks.

Appendix 1, as supplied by the authors. Appendix to: Edwards JD, Li Z, McFarlane P, et al. Management of brain–heart multimorbidity: a clinical practice guideline. *CMAJ* 2026. doi: 10.1503/cmaj.251137. Copyright © 2026 The Author(s) or their employer(s). To receive this resource in an accessible format, please contact us at cmajgroup@cmaj.ca.
