# Supplementary material for: Prise en charge de la multimorbidité cœur–cerveau : un guide de pratique clinique
Source: CMAJ. 2026 May 25;198(20):E784–801. [Article in French] doi: 10.1503/cmaj.251137-f (PMC13218600; doi:10.1503/cmaj.251137-f)
Supplement: Supplementary file 2 [file 251137-guide-2-at.pdf]

Supplemental Figure 1

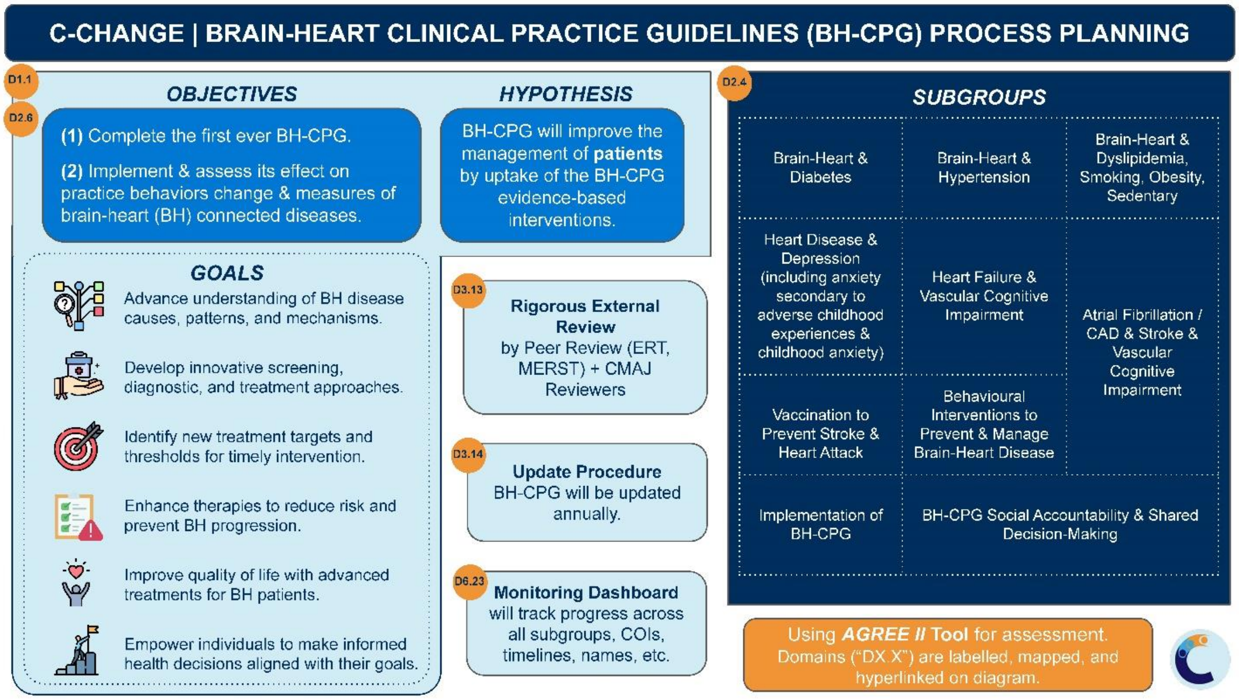

Figure 1: Webpage available to all guideline participants summarizing the main aspects of the Brain-Heart Clinical Practice Guideline and relationship to the Appraisal of Guidelines for Research and Evaluation II (AGREE II) instrument for the appraisal of guidelines.

Appendix 2, as supplied by the authors. Appendix to: Edwards JD, Li Z, McFarlane P, et al. Management of brain–heart multimorbidity: a clinical practice guideline. *CMAJ* 2026. doi: 10.1503/cmaj.251137. Copyright © 2026 The Author(s) or their employer(s). To receive this resource in an accessible format, please contact us at cmajgroup@cmaj.ca.

Supplemental Figure 2

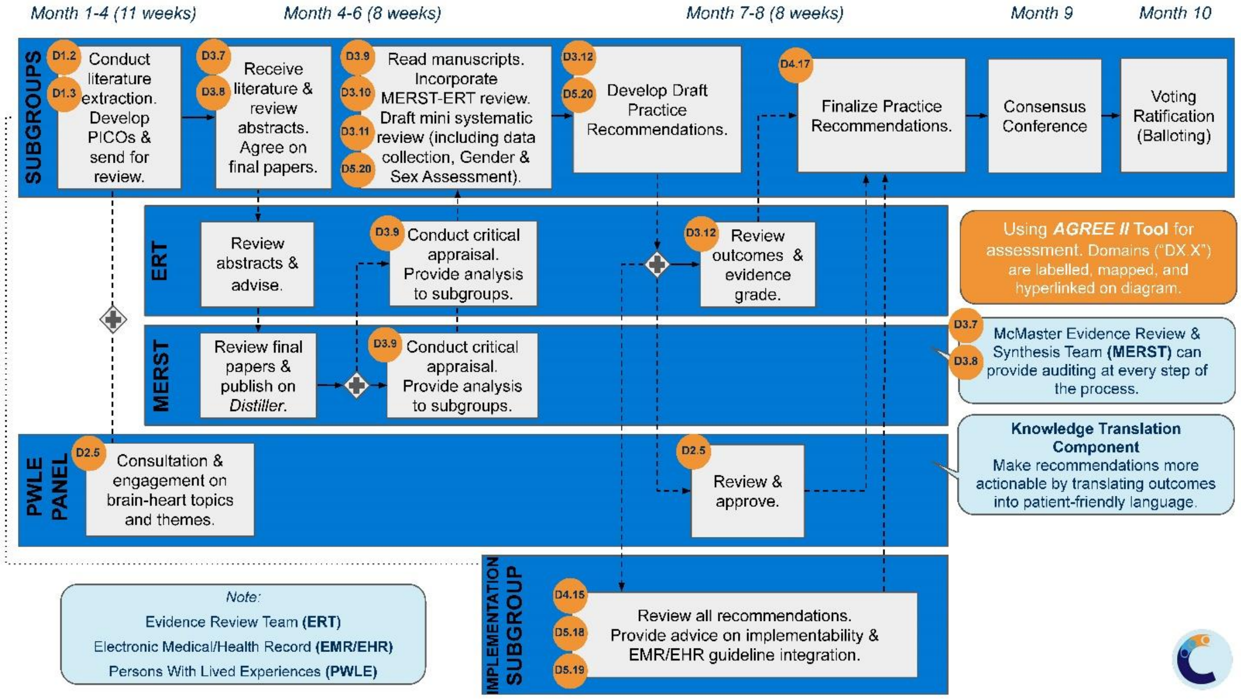

Figure 2: Webpage available to all guideline participants showing the relative timeline to demonstrate how the components of the Brain-Heart Clinical Practice Guideline worked together. The orange dots represent live links to take participants to the Appraisal of Guidelines for Research and Evaluation II (AGREE II) instrument for the appraisal of guidelines. MERST = McMaster Evidence Review and Synthesis Team.
