# Supplementary material for: Prise en charge de la multimorbidité cœur–cerveau : un guide de pratique clinique
Source: CMAJ. 2026 May 25;198(20):E784–801. [Article in French] doi: 10.1503/cmaj.251137-f (PMC13218600; doi:10.1503/cmaj.251137-f)
Supplement: Supplementary file 3 [file 251137-guide-3-at.pdf]

Appendix 3.

Screening and Treatment Recommendation Practice Pearls, and Clinical Considerations

SCREENING RECOMMENDATIONS

1. Atrial Fibrillation and Cognitive Impairment: We suggest that individuals with atrial fibrillation be screened using a validated screening tool to identify those at risk for cognitive impairment (Level of Evidence 2A; Strength of Recommendation B).

Practice Pearls

- Atrial fibrillation independently increases the risk for cognitive impairment.
- Early cognitive screening for individuals with atrial fibrillation allows for better management planning and patient/family support.
- Screening for cognitive impairment can be done either clinically, by history, by report of the individual or family with the understanding that findings may help clinical decision making regarding the need for a more comprehensive assessment.
- Screening is particularly valuable for implementation efforts focused on early lifestyle interventions and cardiovascular risk factor modification, but cognitive screening is valued differently by patients

2. Coronary Artery Disease and Depression: We suggest that individuals with coronary artery disease be screened for depression using a validated screening tool (Level of Evidence is 1B; Strength of Recommendation B).

Practice Pearls

- Screening for depression after coronary events is important for comprehensive care.
- BDI-II and PHQ-2 and PHQ-9 are brief, validated tools suitable for routine use with high sensitivity and specificity for depression.

Appendix 3, as supplied by the authors. Appendix to: Edwards JD, Li Z, McFarlane P, et al. Management of brain–heart multimorbidity: a clinical practice guideline. *CMAJ* 2026. doi: 10.1503/cmaj.251137. Copyright © 2026 The Author(s) or their employer(s). To receive this resource in an accessible format, please contact us at cmajgroup@cmaj.ca.

TREATMENT RECOMMENDATIONS

1. **<u>Coronary Artery Disease and Depression:</u> In individuals with coronary artery disease, if a depression diagnosis is confirmed, we recommend treatment be initiated with an SSRI (serotonin selective reuptake inhibitors) (Level of Evidence 1A Strength of Recommendation A) or we suggest treatment with other evidence-based therapies, such as cognitive behavioural therapy, based on disease severity (Level of Evidence 1B; Strength of Recommendation C).**

Practice Pearl

- Psychotherapy (CBT) and SSRIs can improve depressive symptoms and potentially improve health behaviors and recovery.

<u>2. Hypertension and Cognitive Impairment:</u> We recommend that individuals who have a systolic blood pressure between 130 mm Hg and 180 mm Hg and an increased cardiovascular risk (defined as having clinical or subclinical cardiovascular disease, chronic kidney disease, or a Framingham Risk Score of 15% or greater if patients ≥75 years) undergo intensive blood pressure lowering to less than 120 mm Hg to reduce the risk of cognitive impairment (Level of Evidence 1B; Strength of Recommendation A).

Practice Pearl

- Consider BP targets of <120 mmHg systolic in patients aged ≥75 years with high cardiovascular risk, to reduce the risk of dementia.

<u>3. Dyslipidemia and Stroke/CVD Risk:</u> We recommend that individuals with coronary artery disease with LDL levels above the threshold of 1.8 mmol/L undergo intensification of therapy to bring the LDL below target to prevent stroke and reduce cardiovascular risk (Level of Evidence 1A; Strength of Recommendation A) and we suggest that individuals with stroke with LDL levels above the threshold of 1.8 mmol/L undergo intensification of therapy to bring the LDL below target to reduce the risk of major cardiovascular events (Level of Evidence 1B; Strength of Recommendation B).

Appendix 3, as supplied by the authors. Appendix to: Edwards JD, Li Z, McFarlane P, et al. Management of brain–heart multimorbidity: a clinical practice guideline. *CMAJ* 2026. doi: 10.1503/cmaj.251137. Copyright © 2026 The Author(s) or their employer(s). To receive this resource in an accessible format, please contact us at cmajgroup@cmaj.ca.

Practice Pearls

- Emphasise the *dual cardiovascular and cerebrovascular protection* achieved through intensive lipid lowering.
- Practical messaging for clinicians: "Treat cholesterol intensively and equitably by sex after heart attack or stroke to protect the brain and heart."
- Especially relevant for integration into health behaviours and social accountability discussions.

4. Vaccination and Stroke/CVD Death/MI/Dementia Risk: We recommend to routinely offer all individuals, particularly those over 65, vaccination against influenza to reduce the risk of cardiovascular death and stroke (Level of Evidence 2A, Strength of Recommendation C) and possibly dementia (Level of Evidence 3, Strength of Recommendation C), pneumococcal vaccine to reduce the risk of MI and stroke (Level of Evidence 2A; Strength of Recommendation B), and vaccination against herpes zoster to reduce the risk of MI and stroke (Level of Evidence 2A Strength of Recommendation B), and possibly dementia (Level of Evidence 3; Strength of Recommendation C).

Practice Pearls

- Routine adult vaccination protects not just against infections but also against heart attack, stroke, and dementia.
- Vaccination should be considered part of comprehensive cardiovascular and brain health prevention strategies, especially for adults ≥65 years.

SHARED-DECISION MAKING RECOMMENDATION

1. Shared Decision Making and Heart Brain Health. We recommend clinicians support individuals with or at risk of brain and heart conditions to actively participate in decision-making about their care by using evidence-based decision aids in preparation for, and during consultations to facilitate the shared decision-making process (Level of Evidence 1A; Strength Recommendation B).

Appendix 3, as supplied by the authors. Appendix to: Edwards JD, Li Z, McFarlane P, et al. Management of brain–heart multimorbidity: a clinical practice guideline. *CMAJ* 2026. doi: 10.1503/cmaj.251137. Copyright © 2026 The Author(s) or their employer(s). To receive this resource in an accessible format, please contact us at cmajgroup@cmaj.ca.

Practice Pearls

- Patient decision aids are evidence-based knowledge translation tools designed to help patients make specific and deliberate choices among healthcare options
- Decision aids can significantly enhance patient engagement, knowledge, and alignment of choices with personal values, and supplement clinicians' counseling about options.
- They are particularly important when multiple management options exist, each with different trade-offs (e.g., stroke prevention options, depression treatment strategies, no active treatment).

Appendix 3, as supplied by the authors. Appendix to: Edwards JD, Li Z, McFarlane P, et al. Management of brain–heart multimorbidity: a clinical practice guideline. *CMAJ* 2026. doi: 10.1503/cmaj.251137. Copyright © 2026 The Author(s) or their employer(s). To receive this resource in an accessible format, please contact us at cmajgroup@cmaj.ca.

CLINICAL CONSIDERATIONS

1. Heart Failure and Cognitive Impairment. Consider that individuals with heart failure (HF) be screened for cognitive impairment using a validated screening tool, particularly after a HF hospitalisation, to identify those who will be at higher risk for readmission, poor self-care management, and mortality.

Evidence Summary and Quality. In patients with HF, cognitive impairment (CI) is common[1] and is associated with low medication adherence, lower-levels of self-care management, and impaired functional activities of daily living[2-4]. A meta-analysis of five observational studies (n=2342) showed that CI significantly increased the risk of 30-day rehospitalisation in participants with HF (pooled RR=1.63, 95%CI: 1.19-2.24, I2=64.2%, p=0.002)[5]. Observational data have shown that HF outpatients with Montreal Cognitive Assessment (MoCA) score <26 vs. ≥26 scored significantly lower on the self-care management subscale of the Self-Care in Heart Failure Index (SCHFI) (48.1 SD 24 vs. 59.3 SD 22 respectively, p=0.035)[3] and in a study of 720 consecutive HF patients, poor Mini-Cog performance was identified as the most important predictor among 55 variables by random survival forest analysis[6].

The working group determined that cognitive impairment is a major, under-recognised contributor to worse outcomes in HF. Screening is low risk, low cost, and identifies patients who need additional support for medication adherence and self-care. Even without randomised trial data showing outcome improvement from screening, the strong association between cognitive impairment and adverse outcomes justifies considering routine cognitive screening in HF management based on the individual's values and preferences.

Practice Pearls

- Consider screening for cognitive impairment after HF hospitalization or at the time of HF diagnosis to identify higher-risk patients for readmission.
- Consider using Montreal Cognitive Assessment (MoCA) or Mini-Cog — which are felt to be better suited for HF-related cognitive deficits than the MMSE.
- Screening for CI in people admitted to hospital with HF, supports the need for targeted education, medication adherence strategies, and follow-up care planning.

Appendix 3, as supplied by the authors. Appendix to: Edwards JD, Li Z, McFarlane P, et al. Management of brain–heart multimorbidity: a clinical practice guideline. *CMAJ* 2026. doi: 10.1503/cmaj.251137. Copyright © 2026 The Author(s) or their employer(s). To receive this resource in an accessible format, please contact us at cmajgroup@cmaj.ca.

2. Social Determinants of Heart and Brain Health. Consider screening individuals with cardiovascular risk factors (multiple comorbidities) for adverse social determinants of health with a validated screening tool to identify those at risk of major cardiovascular events..

Evidence Summary and Quality. Social Determinants of Health are non-medical factors that influence health, including but not limited to economic stability (e.g., poverty, unemployment); education access and quality; healthcare access and quality; neighborhood and built environment (e.g., housing, safety); social and community context (e.g., discrimination, social support).

Although no RCT data are available, multiple large cohort studies consistently link adverse social determinants to worse brain and heart outcomes. In a secondary analysis of 11,486 adults aged 70 years and over from the ASPirin in Reducing Events in the Elderly (ASPREE) trial[7], social isolation and support and loneliness were associated with increased hazards of cardiovascular events (HR 1.42, 95% CI: 1.07–1.88) and mortality (HR 2.00 (95% CI: 1.12–3.60). In analyses from the China Health and Retirement Longitudinal Study (CHARLS)[8], high social isolation was associated with incident cardiovascular disease (HR=1.45, 95% CI: 1.13–1.85) and loneliness was associated with an increased risk of stroke (HR=1.68, 95% CI: 1.18–2.40), while exposure to childhood interparental physical violence was associated with increased risks of adult-onset CVD (HR=1.36; 95% CI, 1.20-1.55), heart disease (HR, 1.36; 95% CI, 1.17-1.57), and stroke (HR, 1.28; 95% CI, 1.03-1.61)[9]. In a cross-sectional analysis of 528 community-dwelling older adults from the Korean Social Life, Health, and Aging Project database[10], group-level segregation reduced the odds of cardiovascular health (OR=0.64; 95% confidence interval [CI], 0.43 to 0.95). Data from the UK Biobank diabetes cohort[11] showed that high social isolation increased all-cause mortality (HR 1.33, 95% CI: 1.19–1.47), while loneliness increased the risk of MI and stroke (HR 1.37 (95% CI: 1.19–1.57). Further data from the NHANES study[12] showed that poverty combined with hypertension raised CVD risk across ethnicities (Hispanic population: OR 2.76; Black: OR 3.39; White: OR 2.44) and adverse childhood events have been reported to increased the risk of CVD in adulthood (HR 1.11 (95% CI: 1.08–1.14)[13].

The working group recognised that adverse social determinants of health (SDOH) amplify the risks for brain and heart disease, worsen management burden, and increase mortality. Screening is a crucial step to uncover hidden barriers to health, especially in populations with multimorbidity. Addressing social needs is essential not just for equity, but for effective clinical care. This clinical consideration emphasises the social accountability of healthcare providers and systems to recognise and respond to these broader determinants of health, noting particularly that prior work has highlighted the effectiveness of social interventions that can be implemented in primary care

Appendix 3, as supplied by the authors. Appendix to: Edwards JD, Li Z, McFarlane P, et al. Management of brain–heart multimorbidity: a clinical practice guideline. *CMAJ* 2026. doi: 10.1503/cmaj.251137. Copyright © 2026 The Author(s) or their employer(s). To receive this resource in an accessible format, please contact us at cmajgroup@cmaj.ca.

targeting individual-level determinants, connections with community resources, community-focused partnerships and structures within health teams that affect equity[14].

Practice Pearls

- Screening for social risk factors (e.g., poverty, isolation, food insecurity) requires minimal resources and can uncover barriers to adherence and outcomes.
- Structured tools for screening are available (e.g., social needs screening, Poverty Tool, loneliness scales)
- For patients not adherent to drug therapy, completion of diagnostic testing, and attendance at visits, recognizing "treatment burden" in people with multiple comorbidities and adverse social determinants of health is essential for compassionate and effective care.
- Interprofessional collaboration is key (social workers, case managers, community supports).

Appendix 3, as supplied by the authors. Appendix to: Edwards JD, Li Z, McFarlane P, et al. Management of brain–heart multimorbidity: a clinical practice guideline. *CMAJ* 2026. doi: 10.1503/cmaj.251137. Copyright © 2026 The Author(s) or their employer(s). To receive this resource in an accessible format, please contact us at cmajgroup@cmaj.ca.

References

1. Dodson JA, Truong TT, Towle VR, Kerins G, Chaudhry SI. Cognitive impairment in older adults with heart failure: prevalence, documentation, and impact on outcomes. *Am J Med*. Feb 2013;126(2):120–6. doi:10.1016/j.amjmed.2012.05.029
2. Alosco ML, Spitznagel MB, Raz N, et al. Executive dysfunction is independently associated with reduced functional independence in heart failure. *J Clin Nurs*. Mar 2014;23(5-6):829–36. doi:10.1111/jocn.12214
3. Harkness K, Heckman GA, Akhtar-Danesh N, Demers C, Gunn E, McKelvie RS. Cognitive function and self-care management in older patients with heart failure. *Eur J Cardiovasc Nurs*. Jun 2014;13(3):277–84. doi:10.1177/1474515113492603
4. Alkhuja S, Duffy K. Cognitive impairment and medication adherence in outpatients with heart failure. *Heart Lung*. Sep–Oct 2013;42(5):387. doi:10.1016/j.hrtlng.2012.11.009
5. Kewcharoen J, Trongtorsak A, Kanitsoraphan C, et al. Cognitive impairment and 30-day rehospitalization rate in patients with acute heart failure: A systematic review and meta-analysis. *Indian Heart J*. Jan–Feb 2019;71(1):52–59. doi:10.1016/j.ihj.2018.12.006
6. Patel A, Parikh R, Howell EH, Hsich E, Landers SH, Gorodeski EZ. Mini-cog performance: novel marker of post discharge risk among patients hospitalized for heart failure. *Circ Heart Fail*. Jan 2015;8(1):8–16. doi:10.1161/CIRCHEARTFAILURE.114.001438
7. Freak-Poli R, Ryan J, Neumann JT, et al. Social isolation, social support and loneliness as predictors of cardiovascular disease incidence and mortality. *BMC Geriatr*. Dec 13 2021;21(1):711. doi:10.1186/s12877-021-02602-2
8. Guo L, Wang W, Shi J, Zheng X, Hua Y, Lu C. Evaluation of Social Isolation Trajectories and Incident Cardiovascular Disease Among Middle-Aged and Older Adults in China: National Cohort Study. *JMIR Public Health Surveill*. Jun 30 2023;9:e45677. doi:10.2196/45677
9. Cui C, Liu L, Li H, et al. Childhood Exposure to Interparental Physical Violence and Adult Cardiovascular Disease. *JAMA Netw Open*. Dec 2 2024;7(12):e2451806. doi:10.1001/jamanetworkopen.2024.51806
10. Lee SH, Lee HH, Sung K, Youm Y, Kim HC. Association of group-level segregation with cardiovascular health in older adults: an analysis of data from the Korean Social Life, Health, and Aging Project. *Epidemiol Health*. 2023;45:e2023041. doi:10.4178/epih.e2023041
11. Liang YY, Chen Y, Feng H, et al. Social isolation, loneliness and subsequent risk of major adverse cardiovascular events among individuals with type 2 diabetes mellitus. *Gen Psychiatr*. 2023;36(6):e101153. doi:10.1136/gpsych-2023-101153
12. Sells ML, Blum E, Perry GS, Eke P, Presley-Cantrell L. Excess Burden of Poverty and Hypertension, by Race and Ethnicity, on the Prevalence of Cardiovascular Disease. *Prev Chronic Dis*. Nov 22 2023;20:E109. doi:10.5888/pcd20.230065
13. Wang W, Liu Y, Yang Y, et al. Adverse childhood and adulthood experiences and risk of new-onset cardiovascular disease with consideration of social support: a prospective cohort study. *BMC Med*. Aug 8 2023;21(1):297. doi:10.1186/s12916-023-03015-1

Appendix 3, as supplied by the authors. Appendix to: Edwards JD, Li Z, McFarlane P, et al. Management of brain–heart multimorbidity: a clinical practice guideline. *CMAJ* 2026. doi: 10.1503/cmaj.251137. Copyright © 2026 The Author(s) or their employer(s). To receive this resource in an accessible format, please contact us at cmajgroup@cmaj.ca.

14. Bloch G, Rozmovits L. Implementing social interventions in primary care. *CMAJ*. Nov 8 2021;193(44):E1696–E1701. doi:10.1503/cmaj.210229

Appendix 3, as supplied by the authors. Appendix to: Edwards JD, Li Z, McFarlane P, et al. Management of brain–heart multimorbidity: a clinical practice guideline. *CMAJ* 2026. doi: 10.1503/cmaj.251137. Copyright © 2026 The Author(s) or their employer(s). To receive this resource in an accessible format, please contact us at cmajgroup@cmaj.ca.
