# Supplementary material for: Prise en charge de la multimorbidité cœur–cerveau : un guide de pratique clinique
Source: CMAJ. 2026 May 25;198(20):E784–801. [Article in French] doi: 10.1503/cmaj.251137-f (PMC13218600; doi:10.1503/cmaj.251137-f)
Supplement: Supplementary file 4 [file 251137-guide-4-at.pdf]

Appendix 4

Supplemental Table 2. Sex and Gender Considerations for Recommendations

Recommendations and Clinical Considerations		
**Screening**	**Recommendation**	**Sex and Gender Considerations**
1. Atrial Fibrillation and Cognitive Impairment	We suggest that individuals with atrial fibrillation be screened using a validated screening tool to identify those at risk for cognitive impairment (Level of Evidence 2A; Strength of Recommendation B)	There are some data to suggest that sex differences exist between AF and both cognitive impairment and dementia with females having a stronger positive association compared to males(1, 2). The Canadian Women's Heart Health Alliance Atlas on the Epidemiology, Diagnosis, and Management of Cardiovascular Disease in Women provides a comprehensive review of female sex-specific risks (3).
2. Coronary Artery Disease and Depression:	We suggest that individuals with coronary artery disease be screened for depression using a validated screening tool (Level of Evidence is 1B; Strength of Recommendation B).	There is evidence that depressive symptoms in females are associated with increased risk of coronary artery disease and that depression is more prevalent in females post-ACS(4, 5). Further in those with suspected or established CAD, depressive symptoms are associated with increased risk of death, particularly in younger females(5). In addition to traditional and sex-specific risk factors for CVD (eg, hypertensive disorders of pregnancy), The Lancet Women and Cardiovascular Disease Commission includes depression and anxiety as "under-recognized" risk factors for CVD in females(6). The working group stressed the importance of ensuring that female patients are not underdiagnosed or undertreated.
**Treatment**	**Recommendation**	**Sex and Gender Considerations**
1. Coronary Artery Disease and Depression:	In individuals with coronary artery disease, if a depression diagnosis is confirmed, we recommend treatment be initiated with an SSRI (serotonin selective reuptake inhibitors) (Level of Evidence 1A Strength of Recommendation A) or we suggest treatment with other evidence-based therapies, such as cognitive behavioural or	Prior studies have reported sex differences in antidepressant efficacy in the general population after accounting for medication class, dose and compliance, with a better therapeutic response to tricyclic antidepressants (TCA) for males and a several studies suggesting a better response to SSRIs for females(7). However, due to substantial heterogeneity in trial designs there remains no clear consensus on

Appendix 4, as supplied by the authors. Appendix to: Edwards JD, Li Z, McFarlane P, et al. Management of brain–heart multimorbidity: a clinical practice guideline. *CMAJ* 2026. doi: 10.1503/cmaj.251137. Copyright © 2026 The Author(s) or their employer(s). To receive this resource in an accessible format, please contact us at cmajgroup@cmaj.ca.

	combination therapy, based on disease severity (Level of Evidence 1B; Strength of Recommendation C).	whether there are sex-related efficacy differences in antidepressant treatment(7).
2. Hypertension and Cognitive Impairment:	We suggest that individuals who have a systolic blood pressure between 130 mm Hg and 180 mm Hg and an increased cardiovascular risk (defined as having clinical or subclinical cardiovascular disease, chronic kidney disease, or a Framingham Risk Score of 15% or greater if patients >=75 years) undergo intensive blood pressure lowering to less than 120 mm Hg to reduce the risk of cognitive impairment (Level of Evidence 1B; Strength of Recommendation A).	No major differences in benefit or harm by sex were reported in SPRINT MIND or other related trials(8). The DIRECT Collaboration network meta-analysis reported no effect modification by age or sex(9).
3. Dyslipidemia and Stroke/CVD Risk:	We recommend that individuals with coronary artery disease with LDL levels above the threshold of 1.8 mmol/L undergo intensification of therapy to bring the LDL below target to prevent stroke and reduce cardiovascular risk (Level of Evidence 1A; Strength of Recommendation A) and we suggest that individuals with stroke with LDL levels above the threshold of 1.8 mmol/L undergo intensification of therapy to bring the LDL below target to reduce the risk of major cardiovascular events (Level of Evidence 1B; Strength of Recommendation B).	Benefits of lipid lowering therapy have been observed across both sexes; however, the historical under-treatment of dyslipidemia with both standard and intensive lipid therapy in females remains a major problem(10). Notably, across trials included in the Xie et al. meta-analysis(11), the percentage of males ranged from 75%-83%, highlighting a significant underrepresentation of women overall in lipid lowering trials and in the evidence used to form this recommendation.
4. Vaccination and Stroke/CVD Death/MI/Dementia Risk:	We suggest to routinely offer all individuals, particularly those over 65, vaccination against influenza to reduce the risk of cardiovascular death and stroke (Level of Evidence 2A, Strength of Recommendation C) and possibly dementia (Level of Evidence 3, Strength of Recommendation C), and pneumococcal vaccine to reduce the risk of MI and stroke (Level of Evidence 2A; Strength of	Some data suggest females derive slightly greater cardiovascular protection from vaccination but there is also meta-analytic data showing a higher risk of solicited reactions following influenza vaccines for females compared with males(12, 13).

 Copyright © 2026 The Author(s) or their employer(s). To receive this resource in an accessible format, please contact us at cmajgroup@cmaj.ca.

	Recommendation B), and vaccination against herpes zoster to reduce the risk of MI and stroke (Level of Evidence 2A Strength of Recommendation B), and possibly dementia (Level of Evidence 3; Strength of Recommendation C).	
**Shared-Decision Making**	**Recommendation**	**Sex and Gender Considerations**
1. Shared Decision Making and Heart Brain Health.	We recommend that for individuals with or at risk of brain and heart conditions, clinicians support individuals to actively participate in decision-making about their care by using evidence-based decision support tools in preparation for, and during consultations to facilitate the shared decision-making process (Level of Evidence 1A; Strength Recommendation B).	There is no evidence of sex differences or differential effects by sex; decision aids enhance equitable participation across populations.

 Copyright © 2026 The Author(s) or their employer(s). To receive this resource in an accessible format, please contact us at cmajgroup@cmaj.ca.

Supplemental Table 3. Patient Values and Preferences for Screening and Treatment Recommendations

Recommendations		
**Screening**	**Recommendation**	**Patient Values and Preferences**
1. Atrial Fibrillation and Cognitive Impairment	We suggest that individuals with atrial fibrillation be screened using a validated screening tool to identify those at risk for cognitive impairment (Level of Evidence 2A; Strength of Recommendation B)	Screening for cognitive impairment will be valued differently by patients(18, 19). This recommendation places high value on screening for cognitive impairment either clinically, by history, by report of the individual or family with the understanding that findings may help clinical decision making regarding the need for a more comprehensive assessment. Some patients may value the identification of early cognitive changes rather than those at later stages to allow for implementation of evidence-based management (e.g., healthy lifestyle behaviors, risk factor modification, rhythm control, stroke prophylaxis) that may slow progression, if a diagnosis of cognitive impairment is found. Other patients may be worried about the negative consequences of identification of early cognitive changes and the effect on their quality of life. Hence, it is important to involve patients in how these decisions are made (see Shared Decision Making recommendation).
2. Coronary Artery Disease and Depression:	We suggest that individuals with coronary artery disease be screened for depression using a validated screening tool (Level of Evidence is 1B; Strength of Recommendation B).	Some patients value improvements in mental health and overall quality of life post-ACS. The main rationale is to improve the identification of patients with depressive symptoms at the early stage to allow for implementation of evidence-based management (e.g., medications or behavioural therapy (see Treatment Recommendations). Other patients may have concerns about stigma of being diagnosed with depression and not want to be screened(20). Hence, it is important to involve them in how these decisions are made (see Shared Decision Making recommendation).

Appendix 4, as supplied by the authors. Appendix to: Edwards JD, Li Z, McFarlane P, et al. Management of brain–heart multimorbidity: a clinical practice guideline. *CMAJ* 2026. doi: 10.1503/cmaj.251137. Copyright © 2026 The Author(s) or their employer(s). To receive this resource in an accessible format, please contact us at cmajgroup@cmaj.ca.

Treatment	Recommendation	Patient Values and Preferences
1. Coronary Artery Disease and Depression:	In individuals with coronary artery disease, if a depression diagnosis is confirmed, we recommend treatment be initiated with an SSRI (serotonin selective reuptake inhibitors) (Level of Evidence 1A Strength of Recommendation A) or we suggest treatment with other evidence-based therapies, such as cognitive behavioural or combination therapy, based on disease severity (Level of Evidence 1B; Strength of Recommendation C).	Patients often differ in the value assigned to different types of treatment for depression(21). Among patients, the preference for behavioural based options (e.g., psychotherapy, exercise) is common. Other patients may value use of medication for depression. Hence, it is important to involve them in how these decisions are made (see Shared Decision Making recommendation).
2. Hypertension and Cognitive Impairment:	We suggest that individuals who have a systolic blood pressure between 130 mm Hg and 180 mm Hg and an increased cardiovascular risk (defined as having clinical or subclinical cardiovascular disease, chronic kidney disease, or a Framingham Risk Score of 15% or greater if patients >=75 years) undergo intensive blood pressure lowering to less than 120 mm Hg to reduce the risk of cognitive impairment (Level of Evidence 1B; Strength of Recommendation A).	Patients greatly value dementia prevention but value hypertension treatment differently(22, 23). Some individuals are willing to accept additional medical therapy for the potential of lowering blood pressure and enhancing cognitive preservation and potential to live independently for longer. Whereas, others may not want to add medications. Hence, it is important to involvement them in how these decisions are made (see Shared Decision Making recommendation).
3. Dyslipidemia and Stroke/CVD Risk:	We recommend that individuals with coronary artery disease with LDL levels above the threshold of 1.8 mmol/L undergo intensification of therapy to bring the LDL below target to prevent stroke and reduce cardiovascular risk (Level of Evidence 1A; Strength of Recommendation A) and we suggest that individuals with stroke with LDL levels above the threshold of 1.8 mmol/L undergo intensification of therapy to bring the LDL below target to reduce the risk of major cardiovascular events (Level of Evidence 1B; Strength of Recommendation B).	Patients place a strong priority on stroke and heart attack prevention. However, some patients may have concerns over the potential side effects of statin therapy including muscle aches and diabetes risk(24, 25). A shared decision-making approach is particularly relevant for this recommendation.

 Copyright © 2026 The Author(s) or their employer(s). To receive this resource in an accessible format, please contact us at cmajgroup@cmaj.ca.

4. Vaccination and Stroke/CVD Death/MI/Dementia Risk:	We suggest to routinely offer all individuals, particularly those over 65, vaccination against influenza to reduce the risk of cardiovascular death and stroke (Level of Evidence 2A, Strength of Recommendation C) and possibly dementia (Level of Evidence 3, Strength of Recommendation C), and pneumococcal vaccine to reduce the risk of MI and stroke (Level of Evidence 2A; Strength of Recommendation B), and vaccination against herpes zoster to reduce the risk of MI and stroke (Level of Evidence 2A Strength of Recommendation B), and possibly dementia (Level of Evidence 3; Strength of Recommendation C).	Vaccine hesitancy rates are rising and remain a challenge post-pandemic. Patients appreciate learning about and understanding the broader health benefits of vaccines beyond infection prevention in making decisions about vaccination(26). Some will value these benefits and others will continue to question the benefit of vaccinations. Hence, it is important to involve them in how these decisions are made (see Shared Decision Making recommendation).
**Shared-Decision Making**	**Recommendation**	**Patient Values and Preferences**
1. Shared Decision Making and Heart Brain Health.	We recommend that for individuals with or at risk of brain and heart conditions, clinicians support individuals to actively participate in decision-making about their care by using evidence-based decision support tools in preparation for, and during consultations to facilitate the shared decision-making process (Level of Evidence 1A; Strength Recommendation B).	The majority of Canadians want to have an active role in making healthcare decisions. Decision aids help patients clarify their values for features of options to reach informed values-based preferences. There is growing evidence that decision aids may improve value-congruent choices.

 Copyright © 2026 The Author(s) or their employer(s). To receive this resource in an accessible format, please contact us at cmajgroup@cmaj.ca.

Supplemental Table 4. Benefits and Harms and Implications for Resources, Costs and Implementation for Screening and Treatment Recommendations

**Recommendations**			
**Screening**	**Recommendation**	**Benefits and Harms**	**Implications for Resources, Costs and Implementation**
1. Atrial Fibrillation and Cognitive Impairment	We suggest that individuals with atrial fibrillation be screened using a validated screening tool to identify those at risk for cognitive impairment (Level of Evidence 2A; Strength of Recommendation B)	The evidence indicates that the major benefit of screening it that it identifies cognitive decline early, which may in turn enable earlier intervention on modifiable risk factors (healthy lifestyle behaviors, risk factor modification, rhythm control, stroke prophylaxis). The harms of screening are minimal, as cognitive screening is non-invasive; however, there may be risks of stigma and mis- or overdiagnosis with screening.	Screening is a low-cost intervention with potential downstream benefits for risk reduction if cognitive decline is detected early(27). Providers should consider use of a validated cognitive screening tool (e.g., MoCA, Mini-Cog)(28) during AF management visits and ensure pathways exist for more detailed cognitive assessments if initial screening is abnormal.
2. Coronary Artery Disease and Depression:	We suggest that individuals with coronary artery disease be screened for depression using a validated screening tool (Level of Evidence is 1B; Strength of Recommendation B).	Early identification and treatment of depression improves mental health outcomes and may reduce cardiovascular events. False positives on screening can lead to unnecessary treatment if not carefully evaluated.	Screening for depression in post-ACS patients is low cost(29). Screening should be done early post-ACS (e.g., during hospitalisation or first outpatient visit) and ensure pathways exist for more detailed cognitive assessments if initial screening is abnormal to maximise the potential benefits of intervention.
**Treatment**	**Recommendation**	**Benefits and Harms**	**Implications for Resources, Costs and Implementation**
1. Coronary Artery Disease and Depression:	In individuals with coronary artery disease, if a depression	SSRIs may increase bleeding risks(30), especially when combined	Psychotherapy and exercise programs may have higher costs, but

Appendix 4, as supplied by the authors. Appendix to: Edwards JD, Li Z, McFarlane P, et al. Management of brain–heart multimorbidity: a clinical practice guideline. *CMAJ* 2026. doi: 10.1503/cmaj.251137. Copyright © 2026 The Author(s) or their employer(s). To receive this resource in an accessible format, please contact us at cmajgroup@cmaj.ca.

	diagnosis is confirmed, we recommend treatment be initiated with an SSRI (serotonin selective reuptake inhibitors) (Level of Evidence 1A Strength of Recommendation A) or we suggest treatment with other evidence-based therapies, such as cognitive behavioural or combination therapy, based on disease severity (Level of Evidence 1B; Strength of Recommendation C).	with antiplatelets, and rarely prolong corrected QT interval.	reduce health care utilisation by improving overall health and potentially reducing hospitalisations(31). Access to psychotherapy should be facilitated; SSRIs such as sertraline are reasonable pharmacological options with the most evidence of benefit.
2. Hypertension and Cognitive Impairment:	We suggest that individuals who have a systolic blood pressure between 130 mm Hg and 180 mm Hg and an increased cardiovascular risk (defined as having clinical or subclinical cardiovascular disease, chronic kidney disease, or a Framingham Risk Score of 15% or greater if patients >=75 years) undergo intensive blood pressure lowering to less than 120 mm Hg to reduce the risk of cognitive impairment (Level of Evidence 1B; Strength of Recommendation A).	The benefits of antihypertensive treatment include reduction in cardiovascular events and moderate reductions in the risk of cognitive impairment over available follow-up times. However, across available data, harms may include higher rates of hypotension, syncope, electrolyte abnormalities, and acute kidney injury. Specifically, adverse events in SPRINT MIND included increased hypotension (HR 2.00, 95% CI: 1.59–2.52) and increased acute kidney injury (HR 1.66, 95% CI: 1.19–2.30)(8). No increase in serious fall-related injuries has been observed.	Indications for intensive BP lowering therapies involve the potential for increased resource use as closer monitoring, increased follow-ups and medication adjustments may be required. Hower despite these costs, the potential long-term healthcare savings possible by reducing the risk of or delaying the onset of dementia are substantial, given the high burden of dementia on health systems(32). This recommendation is best applied to those meeting SPRINT high-risk criteria, with baseline systolic BP 130 to 180 mmHg, any of the following: clinical or subclinical cardiovascular disease other than stroke; chronic kidney disease defined as proteinuria < 1 g/d, eGFR 20-59

Appendix 4, as supplied by the authors. Appendix to: Edwards JD, Li Z, McFarlane P, et al. Management of brain–heart multimorbidity: a clinical practice guideline. *CMAJ* 2026. doi: 10.1503/cmaj.251137. Copyright © 2026 The Author(s) or their employer(s). To receive this resource in an accessible format, please contact us at cmajgroup@cmaj.ca.

			mL/min/1.73 m^2), excluding polycystic kidney disease; estimated 10-year global cardiovascular risk ≥ 15%); or age ≥ 75 years(8). This recommendation requires the implementation of standardised BP measurements, medication adjustment and regular follow-up.
3. Dyslipidemia and Stroke/CVD Risk:	We recommend that individuals with coronary artery disease with LDL levels above the threshold of 1.8 mmol/L undergo intensification of therapy to bring the LDL below target to prevent stroke and reduce cardiovascular risk (Level of Evidence 1A; Strength of Recommendation A) and we suggest that individuals with stroke with LDL levels above the threshold of 1.8 mmol/L undergo intensification of therapy to bring the LDL below target to reduce the risk of major cardiovascular events (Level of Evidence 1B; Strength of Recommendation B).	There is clear evidence of benefit for lipid lowering therapies to reduce the risk of recurrent ischemic stroke and major cardiovascular events. The harms of these therapies are generally minimal, with a small risk of diabetes and rare muscle side effects. Statins and PCSK9 inhibitors are well tolerated.	The costs of generic statins are fairly low. While PCSK9 inhibitors are higher cost(33), the evidence indicates that they are justified in very high-risk patients. The cost of medication usage is outweighed by the potential for major savings in direct and indirect health system costs associated with the prevention of stroke and MI in this population(34). Implementation of treatment plans should incorporate LDL targets (<1.8 mmol/L) into routine post-MI and post-stroke care pathways and ensure regular lipid monitoring and therapy adjustment as needed.
4. Vaccination and Stroke/CVD Death/MI/Dementia Risk:	We suggest to routinely offer all individuals, particularly those over 65, vaccination against influenza to reduce the risk of cardiovascular death and stroke	Vaccination shows benefits for the reduction of risk of MI, stroke, hospitalisation, death, and possibly dementia both to the individual and at the broader population-level when sufficient rates of	Vaccination is highly cost-effective, with major downstream savings by preventing hospitalisations for MI, stroke, and infectious diseases(35). Providers are encouraged to offer

	(Level of Evidence 2A, Strength of Recommendation C) and possibly dementia (Level of Evidence 3, Strength of Recommendation C), and pneumococcal vaccine to reduce the risk of MI and stroke (Level of Evidence 2A; Strength of Recommendation B), and vaccination against herpes zoster to reduce the risk of MI and stroke (Level of Evidence 2A Strength of Recommendation B), and possibly dementia (Level of Evidence 3; Strength of Recommendation C).	vaccine are present in a community. Vaccination is association with only minor harms, which may include standard vaccine-associated reactions (e.g., soreness, low-grade fever) and, rarely, a risk of severe allergic reaction.	vaccines systematically at routine visits for adults ≥65 years and those at high cardiovascular risk. In response to hesitancy, it is suggested that clinicians provide information simply with a decision support tool, using the principles of motivational interviewing, with supplemental educational supports in the form of handouts, or links to websites in a timely manner.
**Shared-Decision Making**	**Recommendation**	**Benefits and Harms**	**Implications for Resources, Costs and Implementation**
1. Shared Decision Making and Heart Brain Health.	We recommend that for individuals with or at risk of brain and heart conditions, clinicians support individuals to actively participate in decision-making about their care by using evidence-based decision support tools in preparation for, and during consultations to facilitate the shared decision-making process (Level of Evidence 1A; Strength Recommendation B).	Reported benefits of the use of decision aids were improved knowledge, risk perception accuracy, participation, values clarity, and quality of decision-making. No harms were identified and across studies, decision aids did not cause adverse events, including emotional distress, decisional regret, or worsen clinical outcomes.	There are minimal costs associated with implementing decision aids, especially digital versions. While use of these aids may reduce unnecessary and unwanted interventions, and consultations, evidence for the cost-effectiveness is unclear(36). Care providers are encouraged to integrate decision aids ahead of or during consultations, especially for preference-sensitive decisions (e.g., AF management, lipid therapy intensification). It is important for providers to undergo appropriate training in

 Copyright © 2026 The Author(s) or their employer(s). To receive this resource in an accessible format, please contact us at cmajgroup@cmaj.ca.

			shared decision-making principles and effective use of aids prior to implementation

1. Wood KA, Han F, Ko YA, Wharton WW. Is the association between cognitive disease progression and atrial fibrillation modified by sex? Alzheimers Dement. 2023;19(9):4163–73.
2. Ott A, Breteler MM, de Bruyne MC, van Harskamp F, Grobbee DE, Hofman A. Atrial fibrillation and dementia in a population-based study. The Rotterdam Study. Stroke. 1997;28(2):316–21.
3. Pacheco C, Mullen KA, Coutinho T, Jaffer S, Parry M, Van Spall HGC, et al. The Canadian Women's Heart Health Alliance Atlas on the Epidemiology, Diagnosis, and Management of Cardiovascular Disease in Women - Chapter 5: Sex- and Gender-Unique Manifestations of Cardiovascular Disease. CJC Open. 2022;4(3):243–62.
4. Shanmugasegaram S, Russell KL, Kovacs AH, Stewart DE, Grace SL. Gender and sex differences in prevalence of major depression in coronary artery disease patients: a meta-analysis. Maturitas. 2012;73(4):305–11.
5. Shah AJ, Ghasemzadeh N, Zaragoza-Macias E, Patel R, Eapen DJ, Neeland IJ, et al. Sex and age differences in the association of depression with obstructive coronary artery disease and adverse cardiovascular events. J Am Heart Assoc. 2014;3(3):e000741.
6. Mehran R, Vogel B, Ortega R, Cooney R, Horton R. The Lancet Commission on women and cardiovascular disease: time for a shift in women's health. Lancet. 2019;393(10175):967–8.
7. Sramek JJ, Murphy MF, Cutler NR. Sex differences in the psychopharmacological treatment of depression. Dialogues Clin Neurosci. 2016;18(4):447–57.
8. Group SMIftSR, Williamson JD, Pajewski NM, Auchus AP, Bryan RN, Chelune G, et al. Effect of Intensive vs Standard Blood Pressure Control on Probable Dementia: A Randomized Clinical Trial. JAMA. 2019;321(6):553–61.
9. Peters R, Xu Y, Fitzgerald O, Aung HL, Beckett N, Bulpitt C, et al. Blood pressure lowering and prevention of dementia: an individual patient data meta-analysis. Eur Heart J. 2022;43(48):4980–90.
10. Rodriguez CP, Burka S, Michos ED. Impact of Sex Differences on Lipids and Statin Utilization. Curr Atheroscler Rep. 2025;27(1):38.
11. Xie C, Zhu M, Hu Y, Wang K. Effect of Intensive and Standard Lipid-Lowering Therapy on the Progression of Stroke in Patients With Coronary Artery Syndromes: A Meta-Analysis of Randomized Controlled Trials. J Cardiovasc Pharmacol. 2020;75(3):222–8.
12. Tadount F, Doyon-Plourde P, Rafferty E, MacDonald S, Sadarangani M, Quach C. Is there a difference in the immune response, efficacy, effectiveness and safety of seasonal

influenza vaccine in males and females? - A systematic review. Vaccine. 2020;38(3):444–59.
13. Kiely M, Tadount F, Lo E, Sadarangani M, Wei SQ, Rafferty E, et al. Sex differences in adverse events following seasonal influenza vaccines: a meta-analysis of randomised controlled trials. J Epidemiol Community Health. 2023;77(12):791–801.
14. Lam CSP, Arnott C, Beale AL, Chandramouli C, Hilfiker-Kleiner D, Kaye DM, et al. Sex differences in heart failure. Eur Heart J. 2019;40(47):3859–68c.
15. Solomon SD, McMurray JJV, Anand IS, Ge J, Lam CSP, Maggioni AP, et al. Angiotensin-Neprilysin Inhibition in Heart Failure with Preserved Ejection Fraction. N Engl J Med. 2019;381(17):1609–20.
16. Pitt B, Pfeffer MA, Assmann SF, Boineau R, Anand IS, Claggett B, et al. Spironolactone for heart failure with preserved ejection fraction. N Engl J Med. 2014;370(15):1383–92.
17. Norris CM, Mullen KA, Foulds HJA, Jaffer S, Nerenberg K, Gulati M, et al. The Canadian Women's Heart Health Alliance ATLAS on the Epidemiology, Diagnosis, and Management of Cardiovascular Disease in Women - Chapter 7: Sex, Gender, and the Social Determinants of Health. CJC Open. 2024;6(2Part B):205–19.
18. Martin S, Kelly S, Khan A, Cullum S, Dening T, Rait G, et al. Attitudes and preferences towards screening for dementia: a systematic review of the literature. BMC Geriatr. 2015;15:66.
19. Fowler NR, Perkins AJ, Turchan HA, Frame A, Monahan P, Gao S, et al. Older primary care patients' attitudes and willingness to screen for dementia. J Aging Res. 2015;2015:423265.
20. Mailloux LM, Haas MT, Kennedy SP, DeJongh BM. Implementation and evaluation of depression screening in patients with recently diagnosed coronary artery disease. Ment Health Clin. 2020;10(1):12–7.
21. Gelhorn HL, Sexton CC, Classi PM. Patient preferences for treatment of major depressive disorder and the impact on health outcomes: a systematic review. Prim Care Companion CNS Disord. 2011;13(5).
22. Kandzari DE, Weber MA, Poulos C, Coulter J, Cohen SA, DeBruin V, et al. Patient Preferences for Pharmaceutical and Device-Based Treatments for Uncontrolled Hypertension: Discrete Choice Experiment. Circ Cardiovasc Qual Outcomes. 2023;16(1):e008997.
23. Xu X, Guo T, Liu Z, Chen P, Zhang Y, Ji Q, et al. A Systematic Review of Patient Preferences, Expectations, and Values for the Management and Treatment of Hypertension. Patient Prefer Adherence. 2022;16:2867–76.
24. Tarn DM, Barrientos M, Pletcher MJ, Cox K, Turner J, Fernandez A, et al. Perceptions of Patients with Primary Nonadherence to Statin Medications. J Am Board Fam Med. 2021;34(1):123–31.
25. Ju A, Hanson CS, Banks E, Korda R, Craig JC, Usherwood T, et al. Patient beliefs and attitudes to taking statins: systematic review of qualitative studies. Br J Gen Pract. 2018;68(671):e408–e19.
26. Sanftenberg L, Kuehne F, Anraad C, Jung-Sievers C, Dreischulte T, Gensichen J. Assessing the impact of shared decision making processes on influenza vaccination rates

 Copyright © 2026 The Author(s) or their employer(s). To receive this resource in an accessible format, please contact us at cmajgroup@cmaj.ca.

in adult patients in outpatient care: A systematic review and meta-analysis. Vaccine. 2021;39(2):185–96.
27. Shore J, Kalafatis C, Stainthorpe A, Modarres MH, Khaligh-Razavi SM. Health economic analysis of the integrated cognitive assessment tool to aid dementia diagnosis in the United Kingdom. Front Public Health. 2023;11:1240901.
28. Nasreddine ZS, Phillips NA, Bedirian V, Charbonneau S, Whitehead V, Collin I, et al. The Montreal Cognitive Assessment, MoCA: a brief screening tool for mild cognitive impairment. J Am Geriatr Soc. 2005;53(4):695–9.
29. Yildirim M, Gaynes BN, Keskinocak P, Pence BW, Swann J. The cost-effectiveness of depression screening for the general adult population. J Affect Disord. 2022;303:306–14.
30. Laporte S, Chapelle C, Caillet P, Beyens MN, Bellet F, Delavenne X, et al. Bleeding risk under selective serotonin reuptake inhibitor (SSRI) antidepressants: A meta-analysis of observational studies. Pharmacol Res. 2017;118:19–32.
31. Altmann U, Zimmermann A, Kirchmann HA, Kramer D, Fembacher A, Bruckmayer E, et al. Outpatient Psychotherapy Reduces Health-Care Costs: A Study of 22,294 Insurants over 5 Years. Front Psychiatry. 2016;7:98.
32. Bress AP, Bellows BK, King JB, Hess R, Beddhu S, Zhang Z, et al. Cost-Effectiveness of Intensive versus Standard Blood-Pressure Control. N Engl J Med. 2017;377(8):745–55.
33. Arrieta A, Hong JC, Khera R, Virani SS, Krumholz HM, Nasir K. Updated Cost-effectiveness Assessments of PCSK9 Inhibitors From the Perspectives of the Health System and Private Payers: Insights Derived From the FOURIER Trial. JAMA Cardiol. 2017;2(12):1369–74.
34. Heller DJ, Coxson PG, Penko J, Pletcher MJ, Goldman L, Odden MC, et al. Evaluating the Impact and Cost-Effectiveness of Statin Use Guidelines for Primary Prevention of Coronary Heart Disease and Stroke. Circulation. 2017;136(12):1087–98.
35. Leidner AJ, Murthy N, Chesson HW, Biggerstaff M, Stoecker C, Harris AM, et al. Cost-effectiveness of adult vaccinations: A systematic review. Vaccine. 2019;37(2):226–34.
36. Scalia P, Barr PJ, O'Neill C, Crealey GE, Bagley PJ, Blunt HB, et al. Does the use of patient decision aids lead to cost savings? a systematic review. BMJ Open. 2020;10(11):e036834.
