# Supplementary material for: Prise en charge de la multimorbidité cœur–cerveau : un guide de pratique clinique
Source: CMAJ. 2026 May 25;198(20):E784–801. [Article in French] doi: 10.1503/cmaj.251137-f (PMC13218600; doi:10.1503/cmaj.251137-f)
Supplement: Supplementary file 5 [file 251137-guide-5-at.pdf]

Supplemental Table 5. Author List

Brain – Heart Guideline Panel – Author List and Specialties			
**Author**	**Degree(s)**	**Specialty / Discipline**	**Primary Affiliation**
**Evidence Review Team & McMaster Evidence Review and Synthesis Team(MERST)**			
Jodi D. Edwards	PhD	Epidemiology, Population Health	University of Ottawa Heart Institute, School of Epidemiology and Public Health, University of Ottawa, Ottawa
Zhe Li	PhD	Biostatistics, Epidemiology	University of Ottawa Heart Institute, Ottawa
Michael D. Hill	MD, MSc	Neurology, Stroke Medicine	Department of Clinical Neurosciences & Hotchkiss Brain Institute, Libin Cardiovascular Institute, Cumming School of Medicine, University of Calgary, Calgary, Calgary
Diana Sherifali	RN, PhD	Nursing, Diabetes Care	McMaster University, Hamilton
Megan Racey	PhD	Nutrition, Health Behaviour	McMaster University, Hamilton
Christopher A. Gravel	PhD	Epidemiology, Data Science	University of Ottawa, Ottawa
Meghan Lewis	PhD	Public Health, Epidemiology	University of Ottawa, Ottawa
Sumali Mehta	MSc	Health Research, Data Analysis	University of Ottawa, Ottawa
**Brain-Heart & Diabetes**			
Philip McFarlane	MD, PhD	Nephrology	Department of Medicine, Temerty Faculty of Medicine, University of Toronto
Doreen M. Rabi	MD, MSc	Endocrinology	Libin Cardiovascular Institute, University of Calgary
Jeremy Gilbert	MD	Endocrinology	Sunnybrook Health Sciences Centre, Department of Medicine, Temerty Faculty of Medicine, University of Toronto, Toronto
Diana Sherifali	RN, PhD	Nursing, Diabetes Care	McMaster University, Hamilton
Harpreet S. Bajaj	MD, MPH	Endocrinology	LMC Diabetes & Endocrinology, Brampton
Bradley J. MacIntosh	MSc, PhD	Medical Imaging, Neuroscience	Sunnybrook Health Sciences Centre, Toronto
Karen Tu	MD, MSc	Family Medicine	Department of Medicine, Temerty Faculty of Medicine, University of Toronto
**Brain-Heart & Hypertension**			

Appendix 5, as supplied by the authors. Appendix to: Edwards JD, Li Z, McFarlane P, et al. Management of brain–heart multimorbidity: a clinical practice guideline. *CMAJ* 2026. doi: 10.1503/cmaj.251137. Copyright © 2026 The Author(s) or their employer(s). To receive this resource in an accessible format, please contact us at cmajgroup@cmaj.ca.

Brain – Heart Guideline Panel – Author List and Specialties			
**Author**	**Degree(s)**	**Specialty / Discipline**	**Primary Affiliation**
Jesse Bittman	MD	Internal Medicine	Department of Medicine, University of British Columbia, Vancouver, British Columbia
Ross D. Feldman	MD	Vascular Medicine, Internal Medicine	Western University, London, Ontario
George Dresser	MD	Clinical Pharmacology	Department of Medicine, Western University, London, Ontario
Kristin Terenzi	MD	Family Medicine	Humber River Hospital, Toronto, Ontario
Rick Swartz	MD, PhD	Neurology	Sunnybrook Health Sciences Centre, Toronto
Thuy Pham	NP, MN, MScCH	Nursing, Primary Care, Diabetes Educator	Sunnybrook Health Sciences Centre, Toronto
**Brain-Heart & Dysplipidemia, Smoking, Obesity and Sedentary**			
Jonathan Gabor	MD	Cardiometabolic and Cardiovascular Disease	University of Manitoba, Winnipeg
Glen J. Pearson	PharmD	Clinical Pharmacy	University of Alberta; Faculty of Medicine & Dentistry; Department of Medicine, Division of Cardiology
Peter Selby	MBBS, MHSc	Psychiatry, Addiction Medicine	Department of Medicine, University of Toronto
Sean Wharton	MD, PharmD	Obesity Medicine, Internal Medicine	McMaster University, Hamilton, Ontario
Darren E. R. Warburton	PhD, MSc	Exercise Physiology	University of British Columbia, Vancouver, British Columbia
Smita Pakhalé	MD, MSc	Respirology, Population Health	School of Epidemiology and Public Health, University of Ottawa, Ottawa
**Heart Disease & Depression**			
Rima Styra	MD, MEd	Psychiatry	Department of Psychiatry, University of Toronto
Brian Baker	MBCbB	Psychiatry	Department of Psychiatry, University of Toronto
Michael Hawkins	MD, MSc QIPS	Psychiatry	Department of Psychiatry, University of Toronto

 Copyright © 2026 The Author(s) or their employer(s). To receive this resource in an accessible format, please contact us at cmajgroup@cmaj.ca.

Brain – Heart Guideline Panel – Author List and Specialties			
**Author**	**Degree(s)**	**Specialty / Discipline**	**Primary Affiliation**
James A. Stone	MD, PhD	Cardiology	Libin Cardiovascular Institute, University of Calgary
Tracy Vaillancourt	PhD	Psychology, Education	University of Ottawa Heart Institute, Ottawa
**Heart Disease & Vascular Cognitive Impairment**			
Stephanie Poon	MD, MSc	Cardiology	Sunnybrook Health Sciences Centre, Department of Medicine, University of Toronto, Toronto
Sean A. Virani	MD, MSc, MPH	Cardiology	Department of Medicine, University of British Columbia, Vancouver, British Columbia
Rahul Jain	MD, MScCH	Family Medicine	Sunnybrook Health Sciences Centre, Department of Family and Community Medicine, Temerty Faculty of Medicine, University of Toronto, Toronto
Peter Liu	MD	Cardiology	University of Ottawa Heart Institute, Ottawa
Michael D. Hill	MD, MSc	Neurology, Stroke Medicine	Department of Clinical Neurosciences & Hotchkiss Brain Institute, Libin Cardiovascular Institute, Cumming School of Medicine, University of Calgary, Calgary, Calgary
**Atrial Fibrillation / CAD & Stroke, Vascular Cognitive Impairment**			
Roopinder K. Sandhu	MD	Cardiology, Cardiac Electrophysiology	Libin Cardiovascular Institute, University of Calgary
Aravind Ganesh	MD, DPhil	Neurology, Stroke Medicine	Libin Cardiovascular Institute, University of Calgary
Jason G. Andrade	MD	Cardiac Electrophysiology	Vancouver General Hospital, Vancouver BC
Sol Stern	MD	Family Medicine	Medical Director CME AWAY by Sea Courses, Group Management Lead Argus Medical Centre FHO
Jeffrey Habert	MD	Family Medicine	Department of Family and Community Medicine, Temerty Faculty of Medicine, University of Toronto, Toronto, Canada

Appendix 5, as supplied by the authors. Appendix to: Edwards JD, Li Z, McFarlane P, et al. Management of brain–heart multimorbidity: a clinical practice guideline. *CMAJ* 2026. doi: 10.1503/cmaj.251137. Copyright © 2026 The Author(s) or their employer(s). To receive this resource in an accessible format, please contact us at cmajgroup@cmaj.ca.

Brain – Heart Guideline Panel – Author List and Specialties			
**Author**	**Degree(s)**	**Specialty / Discipline**	**Primary Affiliation**
Léna Rivard	MD, MSc	Cardiology	Department of Medicine, and Research Centre, Montreal Heart Institute, Université de Montréal, Montreal, Canada
Patrice Lindsay	PhD	Stroke, Health Systems, Implementation	MarcLind Health Systems and Engagement Consulting, Toronto
**Vaccination to Prevent Stroke & Heart Attack**			
Paul Roumeliotis	MD, MPH	Pediatrics, Public Health	School of Epidemiology and Public Health, University of Ottawa
Jacob A. Udell	MD, MPH	Cardiology	Peter Munk Cardiac Centre, University Health Network, Cardiovascular Division, Women's College Hospital, Department of Medicine, Temerty Faculty of Medicine, University of Toronto, Toronto
**Decision Aids and Shared Decision Making**			
Krystina B. Lewis	RN, PhD	Nursing, Knowledge Translation	University of Ottawa Heart Institute, School of Nursing, Faculty of Health Sciences, University of Ottawa, Ottawa
Marion Maar	PhD	Indigenous Health, Community Medicine	Northern Ontario School of Medicine University, Sudbury
Dawn Stacey	RN, PhD	Nursing, Decision Science	Faculty of Health Sciences, University of Ottawa, Centre for Implementation Research, Ottawa Hospital Research Institute, Ottawa
Brian Oldenburg	MPsychol, PhD	Public Health, Implementation Science	La Trobe University and Baker Heart and Diabetes Institute, Melbourne, Australia
Karim Keshavjee	MSc, MD	Family Medicine, Health Informatics	Institute of Health Policy, Management and Evaluation, Dalla Lana School of Public Health, University of Toronto, Toronto, Canada
**Social Determinants of Brain-Heart Health**			
Sheldon W. Tobe	MD, MScCH	Nephrology	Sunnybrook Health Sciences Centre / University of Toronto
Marion Maar	PhD	Indigenous Health, Community Medicine	Northern Ontario School of Medicine University, Sudbury

 Copyright © 2026 The Author(s) or their employer(s). To receive this resource in an accessible format, please contact us at cmajgroup@cmaj.ca.

Brain – Heart Guideline Panel – Author List and Specialties			
**Author**	**Degree(s)**	**Specialty / Discipline**	**Primary Affiliation**
Abida R. Dhukai	NP, PhD	Nursing, Primary Care	Sunnybrook Health Sciences Centre / University of Toronto
**Behavioural Interventions to Prevent & Manage Brain-Heart Disease**			
Tavis Campbell	PhD	Psychology, Behavioural Medicine	Libin Cardiovascular Institute, Department of Psychology, University of Calgary
Simon L. Bacon	PhD	Health Psychology	Concordia University, Montreal, Quebec
Luc Trudeau	MD	Internal Medicine	Clinique de la Cite Vaudreuil, Quebec
Gemma Cheng	MD	Family Medicine	McGill University, Montreal, Quebec
Michael D. Hill	MD, MSc	Neurology, Stroke Medicine	Department of Clinical Neurosciences & Hotchkiss Brain Institute, Libin Cardiovascular Institute, Cumming School of Medicine, University of Calgary, Calgary, Calgary
**Electronic Medical Records & Implementation**			
Karim Keshavjee	MSc, MD	Family Medicine, Health Informatics	Institute of Health Policy, Management and Evaluation, Dalla Lana School of Public Health, University of Toronto, Toronto
Nazia Haider	MHI	Health Informatics	University of Toronto, Toronto, Ontario
Peter Hayward Jones	PhD	Health Design, Knowledge Translation	Ontario College of Art & Design University (OCAD), Toronto, Ontario
Sachin V. Pasricha	MD, MPH(c)	Internal Medicine	University of Toronto, Toronto, Ontario
**Executive Committee**			
Peter Liu	MD	Cardiology	University of Ottawa Heart Institute, Ottawa
Sheldon W. Tobe	MD, MScCH	Nephrology	Sunnybrook Health Sciences Centre, Department of Medicine, Temerty Faculty of Medicine, University of Toronto, Toronto
Rahul Jain	MD, MScCH	Family Medicine	Sunnybrook Health Science Centre, Department of Family and Community Medicine, Temerty Faculty of Medicine, University of Toronto, Toronto, Canada

Brain – Heart Guideline Panel – Author List and Specialties			
**Author**	**Degree(s)**	**Specialty / Discipline**	**Primary Affiliation**
Jodi D. Edwards	PhD	Epidemiology, Population Health	University of Ottawa Heart Institute, School of Epidemiology and Public Health, University of Ottawa, Ottawa
Diane Hua-Stewart	MPH, MACP	Public Health, Psychotherapy	Sunnybrook Health Sciences Centre

Appendix 5, as supplied by the authors. Appendix to: Edwards JD, Li Z, McFarlane P, et al. Management of brain–heart multimorbidity: a clinical practice guideline. *CMAJ* 2026. doi: 10.1503/cmaj.251137. Copyright © 2026 The Author(s) or their employer(s). To receive this resource in an accessible format, please contact us at cmajgroup@cmaj.ca.
