# Supplementary material for: Prise en charge de la multimorbidité cœur–cerveau : un guide de pratique clinique
Source: CMAJ. 2026 May 25;198(20):E784–801. [Article in French] doi: 10.1503/cmaj.251137-f (PMC13218600; doi:10.1503/cmaj.251137-f)
Supplement: Supplementary file 6 [file 251137-guide-6-at.pdf]

Appendix 6. Subgroup PICOs, Search Strategies and Evidence Tables

SUBGROUP 1. ATRIAL FIBRILLATION AND STROKE/VASCULAR COGNITIVE IMPAIRMENT

a. **PICO**

In patients presenting with atrial fibrillation, compared to those without atrial fibrillation, should screening for vascular cognitive impairment be performed?

1. **Population**: Adult patient with atrial fibrillation in a) general population and b) post-stroke
2. **Intervention:** Screening
3. **Comparator:** No screening
4. **Outcome:** cognitive impairment

b. **SEARCH STRATEGY**: June/July 2024

Database: OVID Medline Epub Ahead of Print, In-Process & Other Non-Indexed Citations, Ovid MEDLINE(R) Daily and Ovid MEDLINE(R) 1946 to Present
Search Strategy:

----

1 exp Dementia, Vascular/ (7753)
2 (vascular adj3 (cognitive impair* or cognitive dysfunction or cognitive decline or cognitive disorder?)).ti,ab,kf. (2828)
3 vascular dementia.ti,ab,kf. (8471)
4 or/1-3 (13876)
5 (cerebrovascular disorders/ or cerebral small vessel disease/) and (cognitive dysfunction/ or cognitive dysfunction.ti,ab,kf. or cognitive impairment.ti,ab,kf. or dementia.ti,ab,kf.) (2948)
6 4 or 5 (15821)
7 atrial fibrillation/ or atrial fibrillation.ti,ab,kf. (112595)
8 6 and 7 (220)
9 screen*.ti,ab,kf. or mass screening/ (1077151)
10 8 and 9 (8)

Database: EBM Reviews - Cochrane Central Register of Controlled Trials <May 2024>, EBM Reviews - Cochrane Database of Systematic Reviews <2005 to June 19, 2024>
Search Strategy:

----

1 exp Dementia, Vascular/ (438)

Appendix 6, as supplied by the authors. Appendix to: Edwards JD, Li Z, McFarlane P, et al. Management of brain–heart multimorbidity: a clinical practice guideline. *CMAJ* 2026. doi: 10.1503/cmaj.251137. Copyright © 2026 The Author(s) or their employer(s). To receive this resource in an accessible format, please contact us at cmajgroup@cmaj.ca.

2 (vascular adj3 (cognitive impair* or cognitive dysfunction or cognitive decline or cognitive disorder?)).ti,ab,kf. (390)
3 vascular dementia.ti,ab,kf. (922)
4 or/1-3 (1429)
5 (cerebrovascular disorders/ or cerebral small vessel disease/) and (cognitive dysfunction/ or cognitive dysfunction.ti,ab,kf. or cognitive impairment.ti,ab,kf. or dementia.ti,ab,kf.) (134)
6 4 or 5 (1507)
7 atrial fibrillation/ or atrial fibrillation.ti,ab,kf. (15393)
8 6 and 7 (15)
9 screen*.ti,ab,kf. or mass screening/ (98877)
10 8 and 9 (7)

Database: Embase <1974 to 2024 June 21>
Search Strategy:
----
1 multiinfarct dementia/ (15276)
2 (vascular adj3 (cognitive impair* or cognitive dysfunction or cognitive decline or cognitive disorder?)).ti,ab,kf. (4484)
3 vascular dementia.ti,ab,kf. (12556)
4 or/1-3 (21320)
5 cerebrovascular disease.mp. and (cognitive defect/ or dementia/ or cognitive dysfunction.ti,ab,kf. or cognitive impairment.ti,ab,kf.) [mp=title, abstract, heading word, drug trade name, original title, device manufacturer, drug manufacturer, device trade name, keyword heading word, floating subheading word, candidate term word] (11319)
6 4 or 5 (30283)
7 exp atrial fibrillation/ (140782)
8 atrial fibrillation.ti,kw,ab. (177210)
9 7 or 8 (217869)
10 6 and 9 (875)
11 exp screening/ (852336)
12 screen*.ti,ab,kf. (1470239)
13 11 or 12 (1824142)
14 10 and 13 (65)

c. Evidence Table. Atrial Fibrillation and Cognitive Impairment Screening

Lead author, year	Country	Study design	Population (sex/gender,	Intervention/Exposure	Control/Usual care	Outcome (definition and measurement)	No. of participants — Intervention/Exposure	No. of participants —	Treatment/exposure effect on outcome	Sex or gender

Appendix 6, as supplied by the authors. Appendix to: Edwards JD, Li Z, McFarlane P, et al. Management of brain–heart multimorbidity: a clinical practice guideline. *CMAJ* 2026. doi: 10.1503/cmaj.251137. Copyright © 2026 The Author(s) or their employer(s). To receive this resource in an accessible format, please contact us at cmajgroup@cmaj.ca.

			comorbidities, age, ethnicity)					Control/Usual care		analysis reported?
Koh, 2022	International	Systematic review and meta-analysis of prospective cohort studies	General population and post-stroke patients	Atrial fibrillation	No atrial fibrillation	Cognitive impairment or dementia	2 822 974 (general population, 15 studies) Post-stroke cohort (number not totalled)	—	General population: HR 1.39 (95% CI 1.25–1.53; I²=90.3%) Post-stroke: aOR 2.70 (95% CI 1.66–3.74; I²=0.0%)	Not reported
Zhai, 2024	China (UK Biobank)	Prospective cohort study	Adults from UK Biobank, mean follow-up 13.45 years	Atrial fibrillation	No atrial fibrillation	All-cause dementia (ACD), Alzheimer's disease (AD), vascular dementia (VD)	Not separately reported	Not separately reported	ACD: HR 1.79 (95% CI 1.67–1.91) AD: HR 1.48 (95% CI 1.32–1.65) VD: HR 2.46 (95% CI 2.17–2.80) Associations remained significant in competing-risk models	Not reported
Zhang, 2023	United Kingdom (UK Biobank)	Prospective cohort study with propensity-score matching	Adults from UK Biobank (30 601 with AF)	Atrial fibrillation	No atrial fibrillation	All-cause dementia, Alzheimer's disease, vascular dementia	30 601	~472 000 (total cohort)	All-cause dementia: aHR 1.42 (95% CI 1.32–1.52) Vascular dementia: aHR 2.06 (95% CI 1.80–2.36) Alzheimer's disease: aHR 1.08 (95% CI 0.96–1.21) Highest risk if AF diagnosed <65 y (all-cause dementia aHR 1.82; 95% CI 1.54–2.15)	Subgroup analysis by age at AF diagnosis (<65, 65–74, ≥75 years); no sex-stratified results reported
Wood, 2023	United States (NACC database)	Longitudinal secondary analysis of prospective cohort	43 746 participants from 38 US Alzheimer's Disease Research Centres (46%	Atrial fibrillation	No atrial fibrillation	Progression from normal cognition to MCI and from MCI to vascular dementia; incident	~4375 (≈10% with AF at follow-up)	~39 371	Women with AF vs men with AF: Incident MCI OR 3.43 (95% CI 1.55–7.55) Incident dementia OR 3.00 (95% CI 1.22–7.37) Progression normal→MCI HR 1.26 (95% CI 1.06–1.50)	Yes – stronger associations in women than in men

 Copyright © 2026 The Author(s) or their employer(s). To receive this resource in an accessible format, please contact us at cmajgroup@cmaj.ca.

			female, 19% non-White)			dementia and MCI			Progression MCI→vascular dementia HR 3.27 (95% CI 1.89–5.65)		
Ott, 1997	Netherlands (Rotterdam Study)	Prospective population-based cohort	Community-dwelling adults aged ≥55 years	Atrial fibrillation	No atrial fibrillation	Cognitive impairment and dementia	Not reported in excerpt	Not reported in excerpt	Association between AF and cognitive impairment/dementia stronger in women than in men	Yes stronger association in women	–

SUBGROUP 2. HEART DISEASE AND DEPRESSION

a. **PICO**

In individuals with coronary artery disease and depressive symptoms does screening for depression improve depression diagnosis and what are the benefits of subsequent treatment initiation, both pharmacological and non-pharmacological for the reduction of depressive symptoms?

1. **Population**: Individuals with coronary artery disease and depressive symptoms
2. **Intervention:** Screening for depression (confirmatory screening test)
3. **Comparator:** No screening or usual care (without routine screening)
4. **Outcome:** Identification of depression, treatment initiation, improvement in depressive symptoms

b. **SEARCH STRATEGY:** June/July 2024

Database: OVID Medline Epub Ahead of Print, In-Process & Other Non-Indexed Citations, Ovid MEDLINE(R) Daily and Ovid MEDLINE(R) 1946 to Present
Search Strategy:
----
1 myocardial ischemia/ or acute coronary syndrome/ or exp angina pectoris/ or coronary artery disease/ or myocardial infarction/ (330933)
2 (coronary artery disease or myocardial infarction or CABG or coronary artery bypass or myocardial ischemia or Coronary Atherosclerosis or Coronary Arteriosclerosis).ti,ab,kf. (364616)
3 1 or 2 (505002)
4 exp depressive disorder/ (125202)
5 Depression/ (159025)
6 (depression or depressive symptom?).ti,ab,kf. (477021)
7 or/4-6 (540645)

 Copyright © 2026 The Author(s) or their employer(s). To receive this resource in an accessible format, please contact us at cmajgroup@cmaj.ca.

8 3 and 7 (12770)
9 (systematic review or meta-analysis).pt. (351489)
10 "systematic review"/ (264054)
11 meta-analysis/ (202841)
12 (meta analy* or metaanaly* or met analy* or metanaly*).tw. (308870)
13 (systematic* adj3 (review* or overview*)).tw. (361392)
14 randomized controlled trial.pt. (615817)
15 controlled clinical trial.pt. (95562)
16 placebo.ab. (249341)
17 random*.ti,ab,kf. (1531788)
18 or/9-17 (2128675)
19 8 and 18 (2630)
20 animals/ not (animals/ and human/) (5199510)
21 19 not 20 (2585)

Database: EBM Reviews - Cochrane Central Register of Controlled Trials <May 2024>, EBM Reviews - Cochrane Database of Systematic Reviews <2005 to June 19, 2024>
Search Strategy:
----
1 myocardial ischemia/ or acute coronary syndrome/ or exp angina pectoris/ or coronary artery disease/ or myocardial infarction/ (30573)
2 (coronary artery disease or myocardial infarction or CABG or coronary artery bypass or myocardial ischemia or Coronary Atherosclerosis or Coronary Arteriosclerosis).ti,ab,kf. (54039)
3 1 or 2 (64107)
4 exp depressive disorder/ (16712)
5 Depression/ (18245)
6 (depression or depressive symptom?).ti,ab,kf. (96115)
7 or/4-6 (100810)
8 3 and 7 (3111)

Database: Embase <1974 to 2024 June 21>
Search Strategy:
----
1 coronary artery disease/ or exp acute coronary syndrome/ or coronary atherosclerosis/ (311393)
2 exp heart infarction/ (464933)
3 (coronary artery disease or myocardial infarction or CABG or coronary artery bypass or myocardial ischemia or Coronary Atherosclerosis or Coronary Arteriosclerosis).ti,ab,kf. (555008)
4 or/1-3 (832356)
5 exp depression/ or exp dysthymia/ or vascular depression/ (662069)

 Copyright © 2026 The Author(s) or their employer(s). To receive this resource in an accessible format, please contact us at cmajgroup@cmaj.ca.

6 (depression or depressive symptom?).ti,ab,kf. (654915)
7 5 or 6 (907807)
8 4 and 7 (26298)
9 "systematic review"/ (472672)
10 exp meta analysis/ (321249)
11 (meta analy* or metaanaly* or met analy* or metanaly*).tw. (391051)
12 (systematic* adj3 (review* or overview*)).tw. (437279)
13 exp randomized controlled trial/ (830143)
14 placebo.ab. (369273)
15 random*.ti,ab,kf. (2090627)
16 or/9-15 (2819212)
17 8 and 16 (4219)
18 limit 17 to human (4000)

Evidence Table: Coronary Artery Disease and Depression Screening and Treatment

**Author, year**	**Country**	**Study Design**	**Details of PICO/PECO Components**				**Number of participants**		**Treatment/ Exposure effect on outcome *(e.g., difference between the 2 groups)* or Pooled treatment effect (if meta-analysis)**	**Was analysis by Sex reported? Gender? (Y/N checkbox)**
			**Population**<br><br>**Sex/gender**<br>**Comorbidities**<br>**Age**<br>**Ethnicity**	**Intervention/ Exposure *(if a screening tool, or diagnostic test please describe)***	**Control/Usual Care**	**Outcome* (defined and measured)**	**Int/Exp group**	**Con/UC group**		
Tully 2021	Australia (where analysis performed)	Random Effects model meta-analysis	M+F patients with CAD and depression	Antidepressants	Placebo	Depression symptoms reduction,<br><br>**remission,**<br><br>response	384<br><br>**324**	366<br><br>**322**	SMD(std mean diff) -.83 lower[95% CI −1.33 to −0.32] OR 2.06 [95% CI 1.47to2.89]Moderate certainty Low	N

Appendix 6, as supplied by the authors. Appendix to: Edwards JD, Li Z, McFarlane P, et al. Management of brain–heart multimorbidity: a clinical practice guideline. *CMAJ* 2026. doi: 10.1503/cmaj.251137. Copyright © 2026 The Author(s) or their employer(s). To receive this resource in an accessible format, please contact us at cmajgroup@cmaj.ca.

						(50% reduction in depression scores)	447	444	heterogeneity<br><br>OR 2.73[95% CI 1.65 to 4.54]	
Sweda et al 2020	Switzerland(for analysis)	Bayesian meta analysis	Both M+F within 1 year post Acute coronary syndrome (ST, non ST MI or unstable angina)	Antidepressant treatment (SSRI, mirtazapine, buproprion)	RCTs Placebo or usual care in 8/10 trials	All Cause Mort(Primaary)<br>Secondary outcomes MI and Rehospitalization<br><br>Rec MI<br>Rec MI in depression diagnosis	785<br>346<br>779<br>392	687<br>336<br>757<br>361	Median follow-up 12 months with 171 events. Primary outcome, no differences. Low heterogeneity. Secondary analysis also was no difference for MI, but hospitalization was reduced 0.62 (0.40-0.94).<br><br>Restricting to trials of ACS with concomitant depression: Recurrent MI reduced OR 0.45 (0.25-0.81).<br>Repeat hospitalizations from 5 trials 682 people with 220 events.	Analysis by sex not reported.

 Copyright © 2026 The Author(s) or their employer(s). To receive this resource in an accessible format, please contact us at cmajgroup@cmaj.ca.

									Reduced hospitalization vs placebo/usual care by -.62 (0.40-0.94).<br><br>OR .97[95% credible interval (CrI) 0.66–1.42]<br>OR .62[95% CrI 0.40–0.94]<br>OR.64[95% CrI 0.40–1.02]<br>OR .45[CrI 0.25–0.81]	
Nieuwsma, et al. 2017	United States, Canada, UK, Europe	Meta-analysis or systematic review of high-quality RCT	Both M and F (M=77%, F=23%) Post ACS patients adults within 3 months of an identifying ACS event. Mean age range - 57–63 years.	Screening tools such as Prime, BDI-II, HADS, PHQ	DSM validated criterion based clinical interview - PRIME-MD, SCID-I/NP, Structured MINI, DISH	Sensitivity, specificity, negative predictive value [NPV], and positive predictive value [PPV]	6 studies - 1755	Two studies - 50 patients with an MDD diagnosis; the remaining 4 studies had MDD diagnoses in 27 or fewer patients.	BDI-lI - sensitivity of 90% (95% CI, 86% to 92%) and specificity of 80% (CI, 68% to 88%)PPV ranging from 28% to 46% and NPV from 98% to 99%.	N
Marin TS,et al. 2020	21 countries: Australia, Brazil, China,	Meta-analysis or	ACS patients in acute	Use of Screening Tools		Use of any screening tool for	21,790 participa		A total of 17 instruments were reported	N

	Georgia, Germany, Greece,India, Iran, Ireland, Israel, Italy, Jordan, Korea, Norway, Pakistan, Poland, Taiwan, Netherlands,Turkey, and the United States and there was one multi-center trial including USA, Spain, and Australia.	systematic review of high-quality RCT	coronary care - participants (18 years and over) with ACS who may also have had procedures such as coronary artery bypass grafting (CABG), chronic total occlusion, percutaneous coronary intervention (PCI), and percutaneous transluminal coronary angioplasty (PTCA).Forty-four (93.6%) studies reported the balance between sexes (38.1% female).			depression at various time points - All studies screened participants for depressive disorders either at or within a few hours of admission (n = 7), during hospitalization (n = 34), or at, or just prior to, discharge (n = 6). Twenty studies screened participants only once without follow-up. The remaining studies screened at least twice with follow-ups ranging from a few days to six years.	nts		to screen for depression. The most commonly used tools for depression screening were the Beck Depression Inventory (BDI) (16 studies; n = 7063); the HADS (14 studies; n = 2774), and versions of the PHQ (eight studies; n =8753).	

 Copyright © 2026 The Author(s) or their employer(s). To receive this resource in an accessible format, please contact us at cmajgroup@cmaj.ca.

Reavell, 2018	UK, Denmark	Systematic review and metanalysis	patients with CVD (CHD, ACS, AF, or post-MI) and depression or anxiety. Anxiety or depression was defined as either a clinical diagnosis (International Classification of Diseases, DSM or similar) or the presence of anxious and/or depressive symptoms and / or depressive symptoms (≥ a predefined cut-off on a validated questionnaire). Studies of ICD and heart failure	Interventions described as CBT or based on CBT principles were eligible for inclusion. This could have been referred to as CBT, cognitive therapy, or behavioral therapy. However, interventions that solely used the principles of cognitive therapy or behavioral therapy alone were excluded.<br><br>Other psychotherapies (e.g., cognitive analytical therapy,	Eligible comparators were medications, usual care (including other psychological therapies), waiting list control, or no treatment.	The primary outcome was a reduction in anxiety and/or depression in patients with CVD after CBT.<br><br>Patients still classified as depressed and/or anxious according to a validated questionnaire and those no longer meeting the clinical diagnosis at follow-up were also reported. Secondary outcomes included cardiovascular events, cardiovascular mortality, patient satisfaction, and QoL.	Dep = 1144	Dep = 1110	The pooled depression follow-up scores from all 12 studies included 2254 participants<br><br>Depression follow-up scores were significantly lower in CBT patients than in controls (SMD = −0.35, 95% CI = −0.52 to −0.17, p < .001, I^2 = 59%).<br><br>Compared with controls, fewer CBT participants remained depressed after the intervention (odds ratio [OR] = 0.29, 95% CI = 0.12 to 0.69, p = .005, I^2 = 62%).<br><br>One	N

Appendix 6, as supplied by the authors. Appendix to: Edwards JD, Li Z, McFarlane P, et al. Management of brain–heart multimorbidity: a clinical practice guideline. *CMAJ* 2026. doi: 10.1503/cmaj.251137. Copyright © 2026 The Author(s) or their employer(s). To receive this resource in an accessible format, please contact us at cmajgroup@cmaj.ca.

			patients were excluded because recent previous systematic reviews have assessed the effectivene ss of CBT for depression and/or anxiety in these patients	psychodyna mic psychother apy, and interperson al psychother apy) were excluded. Studies that investigated CBT as one element within a mixed intervention package were not included, although CBT with an adjunctive antidepress ant as recommend ed by NICE was accepted		Prespecified subgroup analyses were conducted for each CVD (e.g., CHD, AF, etc) where data were available, the mode of CBT delivery, and different lengths of follow-up. Study data on postcoronar y artery bypass grafting (CABG) participants were included within the CHD subgroup analysis (a CHD history was assumed). Subgroup analyses for the mode of delivery			study reporting the proportion of participants no longer meeting a clinical diagnosis of depression found that the CBT group had significantly fewer participants (31%) no longer meeting the diagnosis for depression compared with the controls (83%) (OR = 0.10, 95% CI = 0.03 to 0.36, p < .001)<br><br>No studies published cardiovascular mortality.<br><br>Two studies including 260 participants reported cardiovascular events. The pooled scores	

 Copyright © 2026 The Author(s) or their employer(s). To receive this resource in an accessible format, please contact us at cmajgroup@cmaj.ca.

						(face-to-face, telephone, and mixed-method sessions), length of CBT course (short: 0–300 minutes, medium: 301–600 minutes, and long: >600 minutes), and length of follow-up (short-term: baseline to ≤3 months, medium-term: >3 months but ≤6 months, and long-term: >6 months) were completed.			showed fewer cardiovascular events in CBT patients compared with the controls; however, this was not statistically significant (OR = 0.80, 95% CI = 0.33 to 1.93, p = .62, I2 = 0%).<br><br>Those receiving CBT were still classified as depressed at follow-up, compared with controls (OR = 0.14, 95%CI = 0.06 to 0.30, p < .001, I2 = 0%); however, anxiety and depression follow-up scores demonstrated considerable heterogeneity (I2 ≥ 75%), so pooling was deemed inappropriate.	

 Copyright © 2026 The Author(s) or their employer(s). To receive this resource in an accessible format, please contact us at cmajgroup@cmaj.ca.

Doyle, 2021	US, netherlands, England, Ireland, denmark	Hybrid Systematic Review and Network Meta-Analysis of Randomized Controlled Trials	18 years or older. At least 70% with a CAD diagnosis (including acute coronary syndrome, angina, and angiographically confirmed coronary disease, treated with percutaneous coronary intervention	psychotherapy (as delivered by trained therapists: e.g., cognitive-behavioral therapy, interpersonal psychotherapy, and counseling) ,	other interventions or a range of comparator groupings were included	Primary outcome: Efficacy (effectiveness) response: **between-group differences in depressive symptoms** Change, standardized mean difference [SMD]) at week 8 after intervention ((or closest measure to 8 weeks that is available, between 4 and 16 weeks)<br><br>Secondary outcomes:<br>- effectiveness at 26 weeks<br><br>-mortality	15 studies = 2446 total participants. Intervention group = 1120.	15 studies = 2446 total participants. control group = 1326.	Results were presented using SMD (standardized mean difference).<br><br>psychotherapy was superior to usual care (-0.44 (-0.68,-0.20). I2 = 78.8%. p=0.000)<br><br>combination was superior to antidepressants (-1.79; -146, -0.12) or psychotherapy alone (-1.16; -1.96, -0.37).<br><br>only one study showed low mortality.	N

 Copyright © 2026 The Author(s) or their employer(s). To receive this resource in an accessible format, please contact us at cmajgroup@cmaj.ca.

Subgroup 3. *HYPERTENSION AND COGNITIVE IMPAIRMENT*

a. PICO

Among hypertensive individuals with mild cognitive impairment or dementia what is the optimal blood pressure target for the prevention of progression of cognitive decline?

- **1. Population**: patients with hypertension with dementia, mild cognitive impairment or at risk for dementia
- **2. Intervention:** Tight blood pressure control
- **3. Comparator:** Standard blood pressure control
- **4. Outcome:** Worsening dementia/cognition

b. SEARCH STRATEGY: June/July 2024

Database: OVID Medline Epub Ahead of Print, In-Process & Other Non-Indexed Citations, Ovid MEDLINE(R) Daily and Ovid MEDLINE(R) 1946 to Present
Search Strategy:
----
1 exp dementia/ or cognitive dysfunction/ (241984)
2 (mild cognitive impairment or dementia).ti,ab,kf. (164255)
3 1 or 2 (300141)
4 (blood pressure adj3 (control or target?)).ti,ab,kf. (24456)
5 ((hypertens* or antihypertensive*).ti,kw,ab. or exp antihypertensive agent/ or dt.fs. or placebo.mp.) and (tight control*.ti,ab,kw. or tightly control*.ti,ab,kf. or intensive control*.ti,ab,kf. or intensive treatment.ti,ab,kf. or tight target?.ti,ab,kf.) (3394)
6 4 or 5 (27579)
7 3 and 6 (295)
8 animals/ not (animals/ and human/) (5199510)
9 7 not 8 (291)

Database: EBM Reviews - Cochrane Central Register of Controlled Trials <May 2024>, EBM Reviews - Cochrane Database of Systematic Reviews <2005 to June 19, 2024>
Search Strategy:
----
1 exp dementia/ or cognitive dysfunction/ (12415)
2 (mild cognitive impairment or dementia).ti,ab,kf. (18778)
3 1 or 2 (24352)

Appendix 6, as supplied by the authors. Appendix to: Edwards JD, Li Z, McFarlane P, et al. Management of brain–heart multimorbidity: a clinical practice guideline. *CMAJ* 2026. doi: 10.1503/cmaj.251137. Copyright © 2026 The Author(s) or their employer(s). To receive this resource in an accessible format, please contact us at cmajgroup@cmaj.ca.

4 (blood pressure adj3 (control or target?)).ti,ab,kf. (7575)
5 ((hypertens* or antihypertensive*).ti,kw,ab. or exp antihypertensive agent/ or dt.fs. or placebo.mp.) and (tight control*.ti,ab,kw. or tightly control*.ti,ab,kf. or intensive control*.ti,ab,kf. or intensive treatment.ti,ab,kf. or tight target?.ti,ab,kf.) (1037)
6 4 or 5 (8410)
7 3 and 6 (116)
8 animals/ not (animals/ and human/) (19587)
9 7 not 8 (116)

Database: Embase <1974 to 2024 June 21>
Search Strategy:
----
1 exp dementia/ (468138)
2 (mild cognitive impairment or dementia).ti,ab,kf. (237534)
3 1 or 2 (513039)
4 (blood pressure adj3 (control or target?)).ti,ab,kf. (36535)
5 ((hypertens* or antihypertensive*).ti,kw,ab. or exp antihypertensive agent/ or dt.fs. or placebo.mp.) and (tight control*.ti,ab,kw. or tightly control*.ti,ab,kf. or intensive control*.ti,ab,kf. or intensive treatment.ti,ab,kf. or tight target?.ti,ab,kf.) (5716)
6 4 or 5 (41839)
7 systematic review/ or meta analysis/ (607062)
8 (meta analy* or metaanaly* or met analy* or metanaly*).tw. (391051)
9 (systematic* adj3 (review* or overview*)).tw. (437279)
10 randomized controlled trial/ (828008)
11 placebo.ab. (369273)
12 (randomi#ed or randomly).ti,ab,kf. (1662811)
13 or/7-12 (2470317)
14 6 and 13 (9066)
15 3 and 14 (149)
16 limit 15 to human (145)

Evidence Table: Hypertension and Cognitive Impairment Treatment

Appendix 6, as supplied by the authors. Appendix to: Edwards JD, Li Z, McFarlane P, et al. Management of brain–heart multimorbidity: a clinical practice guideline. *CMAJ* 2026. doi: 10.1503/cmaj.251137. Copyright © 2026 The Author(s) or their employer(s). To receive this resource in an accessible format, please contact us at cmajgroup@cmaj.ca.

Lead Author (last name), year (published)	Country	Study design	Sex/gender	Comorbidities	Age	Ethnicity	Intervention/ Exposure Describe the intervention, screening tool, or diagnostic test	Control/ Usual Care Describe the comparison group	Outcome *see note* (how its defined and measured)	Number of participants Intervention/ Exposure Group	Number of participants Control/ Usual Care Group	Results Report the Treatment/ Exposure effect on outcome	SGA Was there an analysis by Sex or Gender?
Williamson, J. D., et al	United States and Puerto Rico	RCT	35.6% female, 64.4% male	Hypertension and increased risk of cardiovascular disease	67.9yo	57.7% white, 30.0% black, and 10.5% Hispanic	systolic blood pressure goal of less than 120 mm Hg	systolic blood pressure goal of less than 140 mm Hg	primary cognitive outcome was occurrence of probable dementia Secondary cognitive outcomes included occurrence of MCI	4678	4683	Probable Dementia: -1.4 Cases/1000pt years p=0.1. Mild cognitive impairement -3.7 Cases/1000pts yrs p=0.007 Mild cognitive impairement or probabable dementia -3/9 cases/1000 patient years p=0.01	Sex analysis: Yes , Gener analysis No

Appendix 6, as supplied by the authors. Appendix to: Edwards JD, Li Z, McFarlane P, et al. Management of brain–heart multimorbidity: a clinical practice guideline. *CMAJ* 2026. doi: 10.1503/cmaj.251137. Copyright © 2026 The Author(s) or their employer(s). To receive this resource in an accessible format, please contact us at cmajgroup@cmaj.ca.

									and a composite outcome of occurrence of probable dementia or MCI				
Peters, R., Xu, Y., Fitzgerald, O., Aung, H. L., Beckett, N., Bulpitt, C., Chalmers, J., Forette, F., Gong, J., Harris, K., Humburg, P., Matthews, F. E., Staesse	International	Meta-analysis or systematic review of high-quality RCT	46.8% female	Diabetes 46%	69.1		Treatment of blood pressure	placebo	All Cause dementia:	14040	13968	Odds Ratio 0.8795% CI: 0.756, 0.996) in favour of BP-lowering treatment lowering	Sex analysis: Yes , Male 0.87, Female 0.86 Gener analysis No

Appendix 6, as supplied by the authors. Appendix to: Edwards JD, Li Z, McFarlane P, et al. Management of brain–heart multimorbidity: a clinical practice guideline. *CMAJ* 2026. doi: 10.1503/cmaj.251137. Copyright © 2026 The Author(s) or their employer(s). To receive this resource in an accessible format, please contact us at cmajgroup@cmaj.ca.

n, J. A., Thijs, L., Tzourio, C., Warwick, J., Woodward, M., Anderson, C. S., Dementia rIsk, REduCTion collaboration													
Hughes, D., Judge, C., Murphy, R., Loughlin, E., Costello, M., Whiteley, W., Bosch, J., O'Donnell, M. J., Canavan, M.	International	Meta-analysis or systematic review of high-quality RCT	42% women		69		Blood pressure lowering with antihypertensive agents	Placebo, alternative antihypertensive agent, or higher blood pressure target	dementia or cognitive impairment			Reduced risk of dementia or cognitive impairment: -0.39% [95% CI, 0.09%-0.68%]; I^2 = 0.0%)	Both: No

Appendix 6, as supplied by the authors. Appendix to: Edwards JD, Li Z, McFarlane P, et al. Management of brain–heart multimorbidity: a clinical practice guideline. *CMAJ* 2026. doi: 10.1503/cmaj.251137. Copyright © 2026 The Author(s) or their employer(s). To receive this resource in an accessible format, please contact us at cmajgroup@cmaj.ca.

SUBGROUP 4. DYSLIPIDEMIA and STROKE

a. PICO

In patients with coronary artery disease, does optimal control of dyslipidemia reduce the risk of cerebrovascular events?

1. **Population**: patients with CAD and dyslipidemia (and at least one other risk factor for cerebrovascular events, such as current smoking, obesity as defined by internationally-recognised classifications for BMI in use by the WHO and NIH for White, Hispanic, and Black individuals, or sedentary behaviour)
2. **Intervention:** Control to target lipid levels
3. **Comparator:** Standard lipid control
4. **Outcome:** Ischemic stroke

b. **SEARCH STRATEGY:** June/July 2024

Database: OVID Medline Epub Ahead of Print, In-Process & Other Non-Indexed Citations, Ovid MEDLINE(R) Daily and Ovid MEDLINE(R) 1946 to Present
Search Strategy:

----

1 myocardial ischemia/ or acute coronary syndrome/ or exp angina pectoris/ or coronary artery disease/ or exp myocardial infarction/ (344926)
2 (coronary artery disease or acute coronary syndrome? or myocardial infarction? or angina or myocardial isch#emia).ti,ab,kf. (362337)
3 1 or 2 (494044)
4 exp Dyslipidemias/ (88805)
5 (dyslipid#emia? or hypercholesterol#emia or hyperlipid#emia or hypertriglycerid#emia or (((elevated or high or increased) adj2 lipid?) or cholesterol or triglyceride?)).ti,ab,kf. (371185)
6 4 or 5 (410944)
7 3 and 6 (31740)
8 exp Stroke/ (183089)
9 (stroke or cerebrovascular event? or cerebrovascular accident? or brain h#emorrhage or brain infarct*).ti,ab,kf. (345133)
10 8 or 9 (384550)
11 7 and 10 (4799)
12 (systematic review or meta-analysis).pt. (353423)
13 "systematic review"/ (265775)
14 meta-analysis/ (203863)
15 (meta analy* or metaanaly* or met analy* or metanaly*).tw. (311185)
16 (systematic* adj3 (review* or overview*)).tw. (364387)
17 randomized controlled trial.pt. (617265)
18 controlled clinical trial.pt. (95571)
19 random*.ti,ab,kf. (1538062)

 Copyright © 2026 The Author(s) or their employer(s). To receive this resource in an accessible format, please contact us at cmajgroup@cmaj.ca.

20 or/12-19 (2094014)
21 11 and 20 (1238)
22 animals/ not (animals/ and human/) (5204908)
23 21 not 22 (1235)

Database: EBM Reviews - Cochrane Central Register of Controlled Trials <June 2024>, EBM Reviews - Cochrane Database of Systematic Reviews <2005 to July 10, 2024>
Search Strategy:
----
1 myocardial ischemia/ or acute coronary syndrome/ or exp angina pectoris/ or coronary artery disease/ or exp myocardial infarction/ (31540)
2 (coronary artery disease or acute coronary syndrome? or myocardial infarction? or angina or myocardial isch#emia).ti,ab,kf. (55149)
3 1 or 2 (62441)
4 exp Dyslipidemias/ (9659)
5 (dyslipid#emia? or hypercholesterol#emia or hyperlipid#emia or hypertriglycerid#emia or (((elevated or high or increased) adj2 lipid?) or cholesterol or triglyceride?)).ti,ab,kf. (48092)
6 4 or 5 (50771)
7 3 and 6 (5203)
8 exp Stroke/ (17398)
9 (stroke or cerebrovascular event? or cerebrovascular accident? or brain h#emorrhage or brain infarct*).ti,ab,kf. (68644)
10 8 or 9 (70176)
11 7 and 10 (1357)

Database: Embase <1974 to 2024 July 12>
Search Strategy:
----
1 coronary artery disease/ or exp acute coronary syndrome/ or coronary atherosclerosis/ (313238)
2 exp heart infarction/ or ischemic heart disease/ (590413)
3 (coronary artery disease or acute coronary syndrome? or myocardial infarction? or angina or myocardial isch#emia).ti,ab,kf. (555027)
4 or/1-3 (910400)
5 exp dyslipidemia/ (110576)
6 (dyslipid#emia? or hypercholesterol#emia or hyperlipid#emia or hypertriglycerid#emia or (((elevated or high or increased) adj2 lipid?) or cholesterol or triglyceride?)).ti,ab,kf. (497726)
7 5 or 6 (574477)
8 4 and 7 (86237)
9 exp cerebrovascular accident/ (344922)
10 (stroke or cerebrovascular event? or cerebrovascular accident? or brain h#emorrhage or brain infarct*).ti,ab,kf. (555023)
11 9 or 10 (639940)
12 8 and 11 (17595)

 Copyright © 2026 The Author(s) or their employer(s). To receive this resource in an accessible format, please contact us at cmajgroup@cmaj.ca.

13 "systematic review"/ (476093)
14 exp meta analysis/ (323654)
15 (systematic* adj3 (review* or overview*)).tw. (440613)
16 (meta analy* or metaanaly* or met analy* or metanaly*).tw. (393783)
17 exp randomized controlled trial/ (833602)
18 random*.ti,ab,kf. (2098991)
19 or/13-18 (2735805)
20 12 and 19 (3322)
21 limit 20 to human (3180)

Evidence Table. Dyslipidemia and Stroke

Lead Author (last name), year (published)	Country	Study design	Sex/gender	Comorbidities	Age	Ethnicity	Intervention/ Exposure Describe the intervention, screening tool, or diagnostic test	Control/ Usual Care Describe the comparison group	Outcome *see note* (how its defined and measured)	Number of participants Intervention/ Exposure Group	Number of participants Control/ Usual Care Group	Results Report the Treatment/ Exposure effect on outcome	SGA Was there an analysis by Sex or Gender?
de Caterina et al, 2016	Italy	meta-regression	both M and F	adults from cardiovascular disease prevention trials, HTN, DM2, smoking, prior MI,	40s to 60s	n/a	anti-lipid Rx	placebo, less intensive treatment	stroke, MACE	not totalled	not totalled	Yes	Not stated

Appendix 6, as supplied by the authors. Appendix to: Edwards JD, Li Z, McFarlane P, et al. Management of brain–heart multimorbidity: a clinical practice guideline. *CMAJ* 2026. doi: 10.1503/cmaj.251137. Copyright © 2026 The Author(s) or their employer(s). To receive this resource in an accessible format, please contact us at cmajgroup@cmaj.ca.

				prior stoke									
Dykun et al, 2020	Germany	meta-analysis	both M and F	adults with CVD and comorbidities	50s to 60s	n/a	non-statin anti-lipid Rx	placebo, add on to statin	stroke, MACE	not stated	not stated	Yes	not stated
Atia et al, 2024	Egypt	meta-analysis	both M and F	adults post-MI	adults	n/a	PCSK9-inhibitors	placebo, add on to statin	stroke, MACE, lipids	not totalled	not totalled	Yes	not stated
Xie et al, 2020	China	meta-analysis	both M and F	adults post-MI	adults	n/a	intensive vs standard Rx	less intensive statin Rx	stroke, MACE	not totalled	not totalled	Yes	Yes - Sex analysis
Khan et al, 2021	USA	meta-analysis	both M and F	high-risk 2ary prev	adults	n/a	intensive Rx to target	higher LDL	stroke, MACE	not totalled	not totalled	Yes	not stated
Corvol et al, 2003	France	meta-analysis	both M and F	adults with CAD and comorbidities	adults	n/a	lipid-lowering Rx	placebo, less intensive treatment	stroke	not totalled	not totalled	Yes	Not stated

Appendix 6, as supplied by the authors. Appendix to: Edwards JD, Li Z, McFarlane P, et al. Management of brain–heart multimorbidity: a clinical practice guideline. *CMAJ* 2026. doi: 10.1503/cmaj.251137. Copyright © 2026 The Author(s) or their employer(s). To receive this resource in an accessible format, please contact us at cmajgroup@cmaj.ca.

Koskinas et al, 2018	Europe	meta-analysis	both M and F	ASCVD	adults	n/a	statin and non-statin Rx	less intensive statin Rx	Outcomes from each trial were selected to most closely approximate the composite endpoint of major vascular events (MVEs), which consisted of cardiovascular death, myocardial infarction or other acute coronary syndrome, coronary revascularization, and stroke when available	not totalled	not totalled	Yes	Not stated explicitly
Fulcher 2015	Australia	meta-analysis	both M and F	at-risk for CAD	adults	n/a	statin, intensive statin	placebo, less intensive treatment	Effects on major vascular events, major coronary events,	not totalled	not totalled	Yes	Yes - Sex analysis

Appendix 6, as supplied by the authors. Appendix to: Edwards JD, Li Z, McFarlane P, et al. Management of brain–heart multimorbidity: a clinical practice guideline. *CMAJ* 2026. doi: 10.1503/cmaj.251137. Copyright © 2026 The Author(s) or their employer(s). To receive this resource in an accessible format, please contact us at cmajgroup@cmaj.ca.

									stroke, coronary revascularisation and mortality				
HPS Collaborative Group, 2015	United Kingdom	RCT	both M and F	cerebrovascular disease (stroke, TIA), MI, coronary disease	adults	n/a	statin	placebo	stroke, MACE, lipids	10269	10267	Yes	Yes - Sex analysis
Amarenco, 2006	Europe and USA	RCT	both M and F	recent stroke/TIA	adults	n/a	high-dose (80 mg) atorvastatin	placebo	fatal or non-fatal stroke, MACE	2365	2366	YES	Yes - prespecified adjustment for sex
Amarenco, 2007	Europe and USA	post-hoc analysis of RCT	both M and F	recent stroke/TIA	adults	n/a	high-dose (80 mg) atorvastatin with achieving < 50% LDL reduction	placebo	fatal or non-fatal stroke, MACE	1645	3086	Yes	Yes - prespecified adjustment for sex
Amarenco, 2020	France	RCT	both M and F	recent stroke/TIA	adults	n/a	statin +/- ezetimibe to reach LDL < 1.8	statin to achieve LDL 2.3-2.8	composite primary endpoint of major cardiovascular events	1430	1430	Yes	Yes - adjusted covariate

 Copyright © 2026 The Author(s) or their employer(s). To receive this resource in an accessible format, please contact us at cmajgroup@cmaj.ca.

SUBGROUP 5. VACCINATION FOR PREVENTION OF STROKE/HEART ATTACK/DEMENTIA

a. **PICO**

In adults with an indication for influenza, herpes zoster (shingles), pneumococcal, COVID-19, or RSV vaccination , is there evidence that vaccination reduces the risk of cardiovascular diseases and cerebrovascular diseases compared to those with indications that remain unvaccinated?

1. Population: adults with indications for vaccination against bacterial and viral pathogens (pneumococcus, COVID-19, influenza, herpes zoster or RSV), with no previous history of heart attack, stroke, or cerebrovascular events
2. Intervention: vaccination against bacterial and viral pathogens (e.g. pneumococcus, influenza, COVID-19, RSV, herpes zoster)
3. Comparator: No vaccination
4. Outcome: : Hospitalization for heart attack (MI), stroke, heart failure or death (all-cause or CV death)

b. **SEARCH STRATEGY**: June/July 2024

(((vaccine) AND (adults)) AND (Covid)) AND (cardiovascular)

(((vaccine) AND (adults)) AND (influenza)) AND (cardiovascular)

(((vaccine) AND (adults)) AND (herpes zoster)) AND (cardiovascular)

(((vaccine) AND (adults)) AND (pneumococcal)) AND (cardiovascular)

(((vaccine) AND (adults)) AND (RSV)) AND (cardiovascular)

Evidence Table. Vaccine and Stroke/Heart Attack/Dementia

Author, year	Count ry	Study Design	Details of PICO/PECO Components	Number of participants	Treatment/ Exposure	

Appendix 6, as supplied by the authors. Appendix to: Edwards JD, Li Z, McFarlane P, et al. Management of brain–heart multimorbidity: a clinical practice guideline. *CMAJ* 2026. doi: 10.1503/cmaj.251137. Copyright © 2026 The Author(s) or their employer(s). To receive this resource in an accessible format, please contact us at cmajgroup@cmaj.ca.

			Population<br><br>Sex/gender<br>Comorbidities<br>Age<br>Ethnicity	Intervention/<br>Exposure<br>*(if a screening tool, or diagnostic test please describe)*	Control/Usual Care	Outcome* (defined and measured)	Int/Exp group	Con/UC group	effect on outcome *(e.g., difference between the 2 groups)* or Pooled treatment effect (if meta-analysis)	Was analysis by Sex reported? Gender?<br><br>(Y/N checkbox)
Lophatananon 2023	England	Retrospective population-based chort with electronic health records Clinical Practice Research Datalink (CPRD)	Age 70+, no recorded dementia Hx.<br>Matched to 10 comparators without zoster vaccine. (Matched with age sex etc.)<br>White 80%.	Received Zoster vaccine from Jan 2023 to 2020 (not Shingrix)<br>Received influenza vaccine	HZV 854,745<br>Influenza 742,487.	Dementia	Zoster<br>28,012 (9.9/1000 person years)<br><br>Influenza<br>21,931 (17.2/1000 person-yr)	197,618 (12.1/1000 person-yr)<br><br>120,297 (15.8/1000 person-yr)	Zoster<br>Model3<br>All dementia<br>HR 0.78(95% CI, 0.77-0.79)<br><br>Influenza<br>HR 0.96(95%CI, 0.84-0.97)	Zoster<br>52.3% female<br>Male: HR 0.83 (95%CI, 0.81–0.85)<br>Female: HR 0.74 (95%CI,0.73-0.75)<br><br>Influenza<br>53.6% female<br>Male HR 0.97(95%CI,0.94-0.99)<br>Female HR 0.95(95%CI, 0.93-0.97)
Narii 2023	Japan	Nested case-control of VENUS study. Lined claims to the	Age 65+ .<br>Excluded Hx of MI, matched to 5 comparators at BL	Pneumococcal vaccine. PPSV23 (23 valent PPV), not the PCV 13	No vaccine	Cases/controls<br>AMI, 5,356/26,753<br>Stroke 25,730/128,397	Median 2.5 years<br>AMI 5,356 (1.4%)<br><br>Stroke	AMI 26,753	AMI<br>OR 0.70 (95%CI, 0.62-0.80),<br>Stroke<br>OR 0.81 (95%CI 0.77-0.86)	AMI<br>Male 0.73 (0.60-0.88)<br>Female 0.68(0.57-.81)<br><br>Stroke<br>Male

 Copyright © 2026 The Author(s) or their employer(s). To receive this resource in an accessible format, please contact us at cmajgroup@cmaj.ca.

		national health insurance system and long term care insurance system, with data on 1.4 million		Total in study 383,781						0.83(0.76-0.92) Female 0.80-(0.75-0.86)
Helm 2024	US	Retrospective population-based cohort study	Age 50+. Data frm 2005-2018. Age and sex matched.	Live attenuated zoster vaccine (Zostavax)	No Vaccine	MI and Stroke	27,093<br><br>Total MI 349 (1.29%) Total Stroke 436 (1.61%)	135,465 (38.77% male) Total MI 2,466 (1.82%) Total Stroke 2,984 (2.20%)	MI 0.66 (0.58-0.74) Stroke 0.67(0.60-0.75)	No analysis by sex.
Gupta	Meta-analysis	Controlled studies. 6RCT, 7 retrospective cohorts, 2 case control	Individuals with CVD, mean age 70 (58-77) years, mean duration of follow-up 6 months to 2 years	Influenza vaccine	No vaccine	All cause mortality, cardiovascular death, stroke, MI			All cause mortality OR 0.74 (95%CI, 0.64-0.86) Cardiovascular death OR 0.73 (95%CI, 0.59-0.92) Stroke)R 0.71, (95%CI, 0.57-0.89).	49.9% of vaccinated female, 40.86 unvaccinated female. No analysis by sex or gender.

 Copyright © 2026 The Author(s) or their employer(s). To receive this resource in an accessible format, please contact us at cmajgroup@cmaj.ca.

									MI and HF not significant	

SUBGROUP 6. DECISION AIDS AND SHARED DECISION MAKING

a. PICO

In individuals with brain and/or heart-related conditions do decision aids improve the quality of the decision-making process and decision quality compared to informed consent practices and other health information/education without decision aids?

1. **Population**: individuals with brain and or heart conditions that are addressed across the BHI
2. **Intervention/Exposure:** decision aids
3. **Comparator:** informed consent practices and other health information/education without decision aids
4. **Outcome: Primary:** Decision quality (e.g., informed values-based choice congruence), decision-making process (e.g., knowledge, accurate risk perceptions, decisional conflict, participation in decision-making)

- **Secondary:** System-level measures (e.g., costs, consultation length, resource use)

Evidence Table. Shared Decision Making

Author, year	Country	Study Design	Details of PICO/PECO Components	Number of participants	Treatment/ Exposure effect on outcome *(e.g., difference between the 2 groups)* or Pooled treatment effect (if meta-analysis)	Was analysis by Sex reported? Gender?<br><br>(Y/N checkbox)
Stacey 2024	Canada	Systematic Review with Meta-analyses (209 studies)	**Sex/Gender:**<br>29,227 Male/Men<br>65,113 Female/Women<br>Notes:<br>4/209 studies did not report on sex and/or gender	**Exposure / Intervention Group:**<br>51,063<br><br>Notes:<br>One study (n=279 participants) did not	Informed values-choice congruence: RR 1.75 (95%CI 1.44 to 2.13; 21 studies) favouring decision aid (Moderate certainty of evidence)<br>Knowledge: MD 11.9 (95%CI 10.60 to 13.19; 107 studies) favouring decision aid (High certainty of evidence)	Yes, a manuscript is in preparation.

 Copyright © 2026 The Author(s) or their employer(s). To receive this resource in an accessible format, please contact us at cmajgroup@cmaj.ca.

			Some studies reported on sex and/or gender only for the subset analyzed vs number randomized<br><br>**Comorbidities:**<br>Too heterogeneous (209 studies)<br><br>**Age:** Age was reported in 202/209 studies using a variety of reporting methods (i.e. mean, median, range, categorically)<br><br>**Ethnicity:**<br>Ethnicity and/or Race was reported in 107/209 trials using a variety of categorizations<br><br>**Intervention:**<br>Patient decision aid<br><br>**Control/Usual Care Describe the comparison group:**<br>Usual care (defined as general information, risk assessment, clinical practice guideline summaries for health consumers, placebo intervention (e.g. information on another topic), or no intervention)	report numbers by study arm.<br>Nineteen studies had additional study arms that were not included in analysis (n=3203 participants).<br><br>**Control /Usual Care:**<br>53153	Accurate risk perceptions: RR 1.94 (95%CI 1.61 to 2.34; 25 studies) favouring decision aid (High certainty of evidence)<br>Decisional conflict - Uninformed subscale: MD 10.02 lower (95%CI 12.31 to 7.74 lower; 58 studies) favouring decision aid (High certainty of evidence)<br>Decisional conflict - Unclear values subscale: MD 7.86 lower (95%CI 9.69 to 6.02 lower; 55 studes) favouring decision aid (High certainty of evidence)<br>Participation in decision making (passive role): RR 0.72 (95%CI 0.59 to 0.88; 21 studies) favouring decision aid (High certainty of evidence)<br>Decision regret: MD -1.23 (95% CI -3.05 to 0.59; 22 studies) no difference between decision aid and usual care (High certainty of evidence)<br>Proportion undecided: RR 0.68 (95% CI 0.58 to 0.80; 42 studies) favouring decision aid<br>Patient-clinician communication (measured using OPTION-12): MD 12.14 (95% CI 8.12 to 16.16; 8 studies) favouring decision aid. No difference for other measurement tools (OPTION-5, CollaboRATE, SDM-Q-9). Decision topic discussed with clinician RR 1.42 (95% CI 1.19 to 1.70, 11 studies) favouring decision aid.<br>Satisfaction with the decision-making process: MD 3.33 (95% CI 1.18 to 5.48; 12 studies) favouring decision aid.<br>Preparation for decision-making: MD 9.24 (95% CI 4.78 to 13.71; 7 studies) favouring decision aid when 1 study at high risk of bias removed.	

 Copyright © 2026 The Author(s) or their employer(s). To receive this resource in an accessible format, please contact us at cmajgroup@cmaj.ca.

SUBGROUP 7. HEART FAILURE AND VASCULAR COGNITIVE IMPAIRMENT

a. PICO

In individuals with heart failure (HF), does cognitive screening improve mortality, self-care behaviours/self-management, medication adherence or reduce hospital admissions for HF?

- **1. Population:** : patients with HF across entire LVEF spectrum (HFrEF and HFpEF), in both ambulatory care and inpatient settings
- **2. Intervention:** cognitive screening test (MoCA, MMSE, Mini-Cog, CAMCOG, European Consortium on Alzheimer's disease criteria [ECC])
- **3. Comparator:** no cognitive screening/standard of care/subjective categorization (normal cognitive function vs. mild or severe cognitive impairment)
- **4. Outcome:** HF hospitalization, mortality (all-cause, cardiovascular), self-care behaviors/self-management (European Heart Failure Self-care Behavior Scale), medication adherence

b. SEARCH STRATEGY: Heart Failure and Cognitive Screening

June/July 2024

Database: OVID Medline Epub Ahead of Print, In-Process & Other Non-Indexed Citations, Ovid MEDLINE(R) Daily and Ovid MEDLINE(R) 1946 to Present
Search Strategy:
----
1 exp Heart Failure/ (154498)
2 (heart failure or HFrEF or HFpEF).ti,ab,kf. (232760)
3 1 or 2 (270685)
4 (cognitive dysfunction/ or exp dementia/) and ((screen or screening).ti,ab,kf. or mass screening/) (10441)
5 (screen* adj2 (cognitive or dementia or alzheimer*)).ti,ab,kf. (5687)
6 (MoCA or Montreal Cognitive Assessment or "mini mental state examination" or MMSE or Mini-Cog or CAMCOG or "Cambridge Cognition" or "European Consortium on Alzheimer's disease criteria").ti,ab,kf. and (mass screening/ or screen*.ti,ab,kf. or early detect*.ti,ab,kf.) (5170)
7 or/4-6 (15342)
8 3 and 7 (121)
9 animals/ not (animals/ and human/) (5200524)
10 8 not 9 (121)

Database: EBM Reviews - Cochrane Central Register of Controlled Trials <May 2024>, EBM Reviews - Cochrane Database of Systematic Reviews <2005 to June 26, 2024>
Search Strategy:
----
1 exp Heart Failure/ (14561)
2 (heart failure or HFrEF or HFpEF).ti,ab,kf. (33342)

Appendix 6, as supplied by the authors. Appendix to: Edwards JD, Li Z, McFarlane P, et al. Management of brain–heart multimorbidity: a clinical practice guideline. *CMAJ* 2026. doi: 10.1503/cmaj.251137. Copyright © 2026 The Author(s) or their employer(s). To receive this resource in an accessible format, please contact us at cmajgroup@cmaj.ca.

3 1 or 2 (34890)
4 (cognitive dysfunction/ or exp dementia/) and ((screen or screening).ti,ab,kf. or mass screening/) (680)
5 (screen* adj2 (cognitive or dementia or alzheimer*)).ti,ab,kf. (667)
6 (MoCA or Montreal Cognitive Assessment or "mini mental state examination" or MMSE or Mini-Cog or CAMCOG or "Cambridge Cognition" or "European Consortium on Alzheimer's disease criteria").ti,ab,kf. and (mass screening/ or screen*.ti,ab,kf. or early detect*.ti,ab,kf.) (1654)
7 or/4-6 (2489)
8 3 and 7 (65)
9 animals/ not (animals/ and human/) (19587)
10 8 not 9 (65)

Database: Embase <1974 to 2024 June 25>
Search Strategy:
----
1 exp heart failure/ (680147)
2 (heart failure or HFrEF or HFpEF).ti,ab,kf. (387331)
3 1 or 2 (743417)
4 (exp dementia/ or cognitive defect/) and (screening/ or screening test/) (9918)
5 (screen* adj2 (cognitive or dementia or alzheimer*)).ti,ab,kf. (8712)
6 (MoCA or Montreal Cognitive Assessment or "mini mental state examination" or MMSE or Mini-Cog or CAMCOG or "Cambridge Cognition" or "European Consortium on Alzheimer's disease criteria").ti,ab,kf. and (screening/ or screening test/ or screen*.ti,ab,kf. or early detect*.ti,ab,kf.) (9257)
7 or/4-6 (20790)
8 3 and 7 (471)
9 limit 8 to human (453)

Evidence Table. Heart Failure and Cognitive Impairment

**Author**	**Title**	**What is the target disorder of interest?**	**What risk factor(s) is the study evaluating?**	**What are the outcome(s) in question?**	**Does the study include a group of patients with the**	**Does the paper give a detailed description of inclusion and exclusi**	**Does the paper report follow-up of at least 80% of the subjects**	**Does the paper report statistical adjustment of extraneous prognostic factors**	**Does the paper included detailed description of how the**	**Evidence result**	**Conclusion drawn from the paper**	**Comments from ERT**	**Suggested GRADE**

					target disorder of interest but free of the outcome of interest?	on criteria?	enrolled?	(confounders)?	outcome in question is measured?				
Stepien, K., Furczynska, P., Zalewska, M., Nowak, K., Wlodarczyk, A., Owsianka, I., Skorek, P., Zalewski, J., Nessler, J.	Dementia screening in elderly high-risk patients following heart failure decompensation may predict unfavorable long-term clinical outcomes	Heart failure	Cognitive impairment	Compliance was assessed with the Morisky Medication Adherence Scale. Any incidents of myocardial infarction, coronary revascularization, stroke or transient ischemic attack (TIA), revasculari	Yes	No	No	No	Yes	Level III	Screening diagnosis of dementia (SDD) was established in 37 patients (26%) based on the result of an ALFI-MMSE score of <17 points. During the follow-up, patients with SDD were more often rehospitali	This was only an abstract presented at ESC in 2020, so lower level of evidence	C

 Copyright © 2026 The Author(s) or their employer(s). To receive this resource in an accessible format, please contact us at cmajgroup@cmaj.ca.

				zation, HF hospitalization and bleedings during follow-up were collected.							zed following HF decompensation (48.7% vs. 28.6%, P=0.042) than patients without SDD, despite a similar level of compliance (P=0.25). Irrespective of stroke/TIA history, SDD independently increased the risk of rehospitalization due to HF decompensation (HR 2.22, 95% CI: 1.23-4.01, P=0.007).		

Appendix 6, as supplied by the authors. Appendix to: Edwards JD, Li Z, McFarlane P, et al. Management of brain–heart multimorbidity: a clinical practice guideline. *CMAJ* 2026. doi: 10.1503/cmaj.251137. Copyright © 2026 The Author(s) or their employer(s). To receive this resource in an accessible format, please contact us at cmajgroup@cmaj.ca.

Patel, A., Parikh, R., Howell, E. H., Hsich, E., Landers, S. H., Gorodeski, E. Z.	Mini-cog performance: novel marker of post discharge risk among patients hospitalized for heart failure	Heart failure	Cognitive impairment	Primary outcome was time between hospital discharge and first occurrence of readmission or mortality.	Yes	Yes	Not in actual paper, but perhaps data could have been extracted from supplemental tables	Yes	Yes	Level II	There was a high prevalence of CI as quantified by Mini-Cog performance (23% of cohort). During a mean follow-up time of 6 months, 342 (48%) patients were readmitted, and 24 (3%) died. Poor Mini-Cog performance was an independent predictor of composite outcome (adjusted hazard ratio, 1.90; 95% confidence interval, 1.47-2.44;	Prospective observational cohort study	B

 Copyright © 2026 The Author(s) or their employer(s). To receive this resource in an accessible format, please contact us at cmajgroup@cmaj.ca.

											P<0.0001) and was identified as the most important predictor among 55 variables by random survival forest analysis. Inclusion of Mini-Cog performance in risk models improved accuracy (bootstrapped c-index, 0.602 versus 0.624) and risk reclassification (category-free net reclassification improvement, 27%; 95% confidence		

 Copyright © 2026 The Author(s) or their employer(s). To receive this resource in an accessible format, please contact us at cmajgroup@cmaj.ca.

											interval, 14%-40%; P<0.001). Secondary analysis of initial 30 days post discharge showed effect modification by venue of discharge, whereby patients with CI discharged to a facility had longer time to outcome as compared with those discharged home.		

Appendix 6, as supplied by the authors. Appendix to: Edwards JD, Li Z, McFarlane P, et al. Management of brain–heart multimorbidity: a clinical practice guideline. *CMAJ* 2026. doi: 10.1503/cmaj.251137. Copyright © 2026 The Author(s) or their employer(s). To receive this resource in an accessible format, please contact us at cmajgroup@cmaj.ca.

Harkness, K., Heckman, G. A., Akhtar-Danesh, N., Demers, C., Gunn, E., McKelvie, R. S.	Cognitive function and self-care management in older patients with heart failure	Heart failure	Cognitive impairment	Self-care management (Self-Care Heart Failure Index)	Yes	Yes	Not explicitly stated in text	Yes	Yes	Level III	The presence of MCI, as defined by a MoCA score <26, was present in 73% patients; 21% had an adequate self-care management SCHFI score; and 12% reported symptoms of depression. Participants with a MoCA score <26 vs. ≥ 26 scored significantly lower on the self-care management subscale of the SCHFI (48.1 SD 24 vs.	Observational, cross-sectional study	C

Appendix 6, as supplied by the authors. Appendix to: Edwards JD, Li Z, McFarlane P, et al. Management of brain–heart multimorbidity: a clinical practice guideline. *CMAJ* 2026. doi: 10.1503/cmaj.251137. Copyright © 2026 The Author(s) or their employer(s). To receive this resource in an accessible format, please contact us at cmajgroup@cmaj.ca.

											59.3 SD 22 respectively, p=0.035). Using backward regression, the final model was fitted to self-care management while controlling for age and sex and was significant, with (F= 7.04 df (3, 96), and p<0.001), accounting for 18% of the total variance in self-care management (R2 = 18.03%). The MoCA score was the only variable which remained in the model		

 Copyright © 2026 The Author(s) or their employer(s). To receive this resource in an accessible format, please contact us at cmajgroup@cmaj.ca.

											significantly with p= 0.001.Conclusion: Findings from this study highlight the difficulty older heart failure patients have with self-care management and the need to include formal screening for MCI when exploring variables contributing to self-care management in heart failure patients.		

 Copyright © 2026 The Author(s) or their employer(s). To receive this resource in an accessible format, please contact us at cmajgroup@cmaj.ca.

Kewcharoen, J., Trongtorsak, A., Kanitsoraphan, C., Prasitlumkum, N., Mekritthikrai, R., Techorueangwiwat, C., Limpruttidham, N., Rattanawong, P.	Cognitive impairment and 30-day rehospitalization rate in patients with acute heart failure: A systematic review and meta-analysis	Heart failure	Cognitive impairment	Risk of 30-day rehospitalization in HF patients	Yes	Yes	NA	Yes	Yes	Level I	Five studies with a total of 2,342 participants (1,004 participants had cognitive impairment) were included for meta-analysis. In random-effect model, cognitive impairment significantly increased the risk of 30-day rehospitalization in HF participants (pooled RR=1.63, 95%CI: 1.19-2.24], I2=64.2%, p=0.002). Subgroup analysis was performed	meta-analysis showing presence of cognitive impairment results in higher 30 day readmission rates for patients with HF	NA

 Copyright © 2026 The Author(s) or their employer(s). To receive this resource in an accessible format, please contact us at cmajgroup@cmaj.ca.

											on the studies that excluded patients with dementia. The results also showed that cognitive impairment significantly increased the risk of 30-day rehospitalization in participants with HF (pooled RR=1.29, 95%CI: 1.05-1.59, I2=0.0%, p=0.016), which was consistent with our overall analysis. Conclusion(s): Our meta-analysis demonstra		

 Copyright © 2026 The Author(s) or their employer(s). To receive this resource in an accessible format, please contact us at cmajgroup@cmaj.ca.

											ted that the presence of cognitive impairment is associated with 30-day rehospitalization in patients with HF.		

SUBGROUP 8. SOCIAL DETERMINANTS OF HEART BRAIN HEALTH

a. **PICO**

In people with brain and heart comorbidities does history of multiple adverse social determinants of health increase the risk and incidence of major cardiovascular risk factors and events compared to those without or with few adverse social determinants?

1. **Population**: Patients with both brain and heart comorbidities, like heart attack and stroke, or heart attack and dementia, or heart failure and dementia, atherosclerotic CV disease and depression or addictions, heart disease and adverse childhood events or anxiety, or sexual/physical abuse

2. **Intervention/Exposure:** *adverse social determinants of health:* poverty, low education, food insecurity, low access to health care (no primary care), water insecurity, poor social support (loss of spouse/caregiver), isolation, loneliness, homeless (unhoused), grief, gender roles (risky behaviours)
3. **Comparator:** No adverse SDH
4. **Outcome:** MI, stroke, all cause death, CV death, hospitalized heart failure, hypertension, diabetes, dyslipidemia, sedentary lifestyle, adherence to diagnostic, screening tests and medical treatment and procedures, CABG, cardiac rehab.

b. SEARCH STRATEGY: June/July 2024

Database: OVID Medline Epub Ahead of Print, In-Process & Other Non-Indexed Citations, Ovid MEDLINE(R) Daily and Ovid MEDLINE(R) 1946 to Present
Search Strategy:
----
1 exp heart failure/ or exp myocardial ischemia/ (614896)
2 (myocardial infarction or heart failure or hfref or hfpEF or atherosclerosis or cardiovascular disease?).ti,ab,kf. (744207)
3 1 or 2 (1060517)
4 exp Stroke/ (182685)
5 exp dementia/ (216324)
6 depression/ or exp depressive disorder/ (267093)
7 anxiety/ or exp anxiety disorders/ (192358)
8 exp Substance-Related Disorders/ (319809)
9 (stroke or dementia or alzheimer* or depression or depressive symptom* or anxiety or anxiety symptom* or addiction* or substance-use disorder? or alcoholism or excessive alcohol or binge drink* or sexual abuse or sexual assault or physical abuse or drug abuse or substance abuse or substance dependence or alcohol dependence or drug dependence).ti,ab,kf. (1374813)
10 exp Sex O\enses/ (28505)
11 or/4-10 (1779346)
12 3 and 11 (112807)
13 social problems/ or adverse childhood experiences/ or exp poverty/ (62916)
14 exp Food Insecurity/ or water insecurity/ (1949)
15 social environment/ or exp social isolation/ or exp social marginalization/ or social vulnerability/ (71036)
16 exp Ill-Housed Persons/ (11542)
17 Loneliness/ (7072)
18 exp bereavement/ (15644)
19 healthcare disparities/ or socioeconomic disparities in health/ or "social determinants

 Copyright © 2026 The Author(s) or their employer(s). To receive this resource in an accessible format, please contact us at cmajgroup@cmaj.ca.

of health"/ (30609)
20 Access to Primary Care/ (43)
21 (adverse childhood experience? or social marginali#* or social vulnerability or homeless* or unhoused or social* isolat* or (access adj2 (primary care or health services)) or food insecurity or water insecurity or "lack of food" or food dessert? or poverty or loneliness or grief or living condition? or health inequity).ti,ab,kf. (117396)
22 or/13-21 (257875)
23 12 and 22 (1180)
24 animals/ not (animals/ and human/) (5200252)
25 23 not 24 (1171)
Database: Embase <1974 to 2024 June 26>Search Strategy:
----
1 heart disease/ or cardiovascular disease/ or exp heart failure/ or exp ischemic heart disease/ (1648886)
2 (myocardial infarction or heart failure or hfref or hfpEF or atherosclerosis or cardiovascular disease?).ti,ab,kf. (1125823)
3 1 or 2 (1979464)
4 exp cerebrovascular accident/ (342973)
5 exp dementia/ (468473)
6 exp depression/ (662581)
7 anxiety/ or exp anxiety disorder/ (591190)
8 substance abuse/ or addiction/ or exp drug dependence/ (370100)
9 (stroke or dementia or alzheimer* or depression or depressive symptom* or anxiety or anxiety symptom* or addiction* or substance-use disorder? or alcoholism or excessive alcohol or binge drink* or sexual abuse or sexual assault or physical abuse or drug abuse or substance abuse or substance dependence or alcohol dependence or drug dependence).ti,ab,kf. (1951772)
10 exp sexual abuse/ or exp sexual assault/ (43547)
11 or/4-10 (2717513)
12 3 and 11 (295216)
13 childhood adversity/ or exp childhood trauma/ (24441)
14 exp poverty/ (57998)
15 exp food insecurity/ (10041)
16 water insecurity/ (945)
17 social exclusion/ (2886)
18 social vulnerability/ or socioeconomic vulnerability/ (1358)
19 social isolation/ or emotional deprivation/ or social environment/ (73202)
20 homelessness/ (14225)

Appendix 6, as supplied by the authors. Appendix to: Edwards JD, Li Z, McFarlane P, et al. Management of brain–heart multimorbidity: a clinical practice guideline. *CMAJ* 2026. doi: 10.1503/cmaj.251137. Copyright © 2026 The Author(s) or their employer(s). To receive this resource in an accessible format, please contact us at cmajgroup@cmaj.ca.

21 housing instability/ or exp social inequality/ or exp social stress/ or unmet medical need/ (69208)
22 loneliness/ (16219)
23 health care access/ or primary care access/ (95636)
24 "social determinants of health"/ (24245)
25 exp grief/ (16752)
26 (adverse childhood experience? or social marginali#* or social vulnerability or homeless* or unhoused or social* isolat* or (access adj2 (primary care or health services)) or food insecurity or water insecurity or "lack of food" or food dessert? or poverty or loneliness or grief or living condition? or health inequity).ti,ab,kf. (142580)
27 or/13-26 (422241)
28 12 and 27 (5988)
29 limit 28 to human (5790)
Database: EBM Reviews - Cochrane Central Register of Controlled Trials <May 2024>, EBM Reviews - Cochrane Database of Systematic Reviews <2005 to June 26, 2024>Search Strategy:
----
1 exp heart failure/ or exp myocardial ischemia/ (51560)
2 (myocardial infarction or heart failure or hfref or hfpEF or atherosclerosis or cardiovascular disease?).ti,ab,kf. (90368)
3 1 or 2 (110594)
4 exp Stroke/ (17301)
5 exp dementia/ (9335)
6 depression/ or exp depressive disorder/ (30010)
7 anxiety/ or exp anxiety disorders/ (20521)
8 exp Substance-Related Disorders/ (21025)
9 (stroke or dementia or alzheimer* or depression or depressive symptom* or anxiety or anxiety symptom* or addiction* or substance-use disorder? or alcoholism or excessive alcohol or binge drink* or sexual abuse or sexual assault or physical abuse or drug abuse or substance abuse or substance dependence or alcohol dependence or drug dependence).ti,ab,kf. (226084)
10 exp Sex O\enses/ (699)
11 or/4-10 (248480)
12 3 and 11 (20106)
13 social problems/ or adverse childhood experiences/ or exp poverty/ (2558)
14 exp Food Insecurity/ or water insecurity/ (42)
15 social environment/ or exp social isolation/ or exp social marginalization/ or social vulnerability/ (1591)
16 exp Ill-Housed Persons/ (0)

 Copyright © 2026 The Author(s) or their employer(s). To receive this resource in an accessible format, please contact us at cmajgroup@cmaj.ca.

17 Loneliness/ (254)
18 exp bereavement/ (348)
19 healthcare disparities/ or socioeconomic disparities in health/ or "social determinants of health"/ (413)
20 Access to Primary Care/ (0)
21 (adverse childhood experience? or social marginali#* or social vulnerability or homeless* or unhoused or social* isolat* or (access adj2 (primary care or health services)) or food insecurity or water insecurity or "lack of food" or food dessert? or poverty or loneliness or grief or living condition? or health inequity).ti,ab,kf. (7222)
22 or/13-21 (11022)
23 12 and 22 (119)
24 animals/ not (animals/ and human/) (19587)
25 23 not 24 (119)

 Copyright © 2026 The Author(s) or their employer(s). To receive this resource in an accessible format, please contact us at cmajgroup@cmaj.ca.

Author, year	Country	Study Design	Details of PICO/PECO Components				Number of participants		Treatment/ Exposure effect on outcome *(e.g., difference between the 2 groups)* or Pooled treatment effect (if meta-analysis)	Was analysis by Sex reported? Gender? (Y/N checkbox)
			Population<br><br>Sex/gender<br>Comorbidities<br>Age<br>Ethnicity	Intervention/ Exposure *(if a screening tool, or diagnostic test please describe)*	Control/Usual Care	Outcome* (defined and measured)	Int/Exp group	Con/UC group		
Liang YY, 2023	China, but using the UK biobank	Longitudinal study	19,360, 61.1% male.(no gender info) Ethnicity 87.8% white, Smokers 11.5%, Depression 1.5%, dyslipidemia 68.5%, diabetes type 2100%, CKD 1.7%, BP 145/83, type 2 diabetes duration 4 years. Exclude IHD including (MI, stroke, cancer at BL)	Exposure: Social isolation by validated questionnaire with a social isolation index score indicating little contact with family/friends,or living alone. Loneliness by short-term UCLA Loneliness Scale indicating feeling lonely and not being able to confide in anyone.	Not socially isolated or lonely	MACE: all cause mortality, CVD mortality, first MI or stroke, death from MI or stroke	<u>Social Isolation</u><br>Moderate 8147, most 3804<br><br>Few contacts 9601<br><br>Living alone 4602<br><br><u>Loneliness</u><br>Loneliness 1487	Least 7409<br><br>More contacts 9759<br><br>Not living alone 14758<br><br>No loneliness 17873	<u>Social Isolation</u><br><br><u>Mortality All cause</u><br>moderate vs least 1.15 (1.06-1.27), most vs least 1.33 (1.19-1.47)<br>More vs few contacts 1.10 (1.02-1.19)<br>Living alone no vs yes 1.31 (1.20-1.42)<br><br><u>CVD Mortality</u><br>moderate vs least 1.22(1.08-1.39), most	Yes:<br>No diff by sex for social isolation or loneliness subgroups

Appendix 6, as supplied by the authors. Appendix to: Edwards JD, Li Z, McFarlane P, et al. Management of brain–heart multimorbidity: a clinical practice guideline. *CMAJ* 2026. doi: 10.1503/cmaj.251137. Copyright © 2026 The Author(s) or their employer(s). To receive this resource in an accessible format, please contact us at cmajgroup@cmaj.ca.

									vs least<br>1.36 (1.17-1.59)<br>More vs few contacts<br>1.13 (1.01-1.26)<br>Living alone no vs yes 1.37 (1.22-1.55)<br><br>Fatal MI or stroke<br>Most vs Least 1.89 (1.24-2.87)<br>Living alone no vs yes 1.60 (1.14-2.24)<br><br>Loneliness<br><br>Mortality<br>All cause<br>NS<br>CVD Mortality<br>moderate vs least<br>NS<br><br>Composite nonfatal MI or Stroke<br>Not lonely vs lonely<br>1.37 (1.19-1.57)	

Appendix 6, as supplied by the authors. Appendix to: Edwards JD, Li Z, McFarlane P, et al. Management of brain–heart multimorbidity: a clinical practice guideline. *CMAJ* 2026. doi: 10.1503/cmaj.251137. Copyright © 2026 The Author(s) or their employer(s). To receive this resource in an accessible format, please contact us at cmajgroup@cmaj.ca.
